# The Australasian dingo archetype: *de novo* chromosome-length genome assembly, DNA methylome, and cranial morphology

**DOI:** 10.1093/gigascience/giad018

**Published:** 2023-03-28

**Authors:** J William O Ballard, Matt A Field, Richard J Edwards, Laura A B Wilson, Loukas G Koungoulos, Benjamin D Rosen, Barry Chernoff, Olga Dudchenko, Arina Omer, Jens Keilwagen, Ksenia Skvortsova, Ozren Bogdanovic, Eva Chan, Robert Zammit, Vanessa Hayes, Erez Lieberman Aiden

**Affiliations:** School of Biosciences, University of Melbourne, Royal Parade, Parkville, Victoria 3052, Australia; Department of Environment and Genetics, SABE, La Trobe University, Melbourne, Victoria 3086, Australia; Centre for Tropical Bioinformatics and Molecular Biology, College of Public Health, Medical and Veterinary Science, James Cook University, Cairns, Queensland 4870, Australia; Immunogenomics Lab, Garvan Institute of Medical Research, Darlinghurst, NSW 2010, Australia; School of Biotechnology and Biomolecular Sciences, University of New South Wales, Sydney, NSW 2052, Australia; School of Archaeology and Anthropology, The Australian National University, Acton, ACT 2600, Australia; School of Biological, Earth and Environmental Sciences, University of New South Wales, Sydney, NSW 2052, Australia; Department of Archaeology, School of Philosophical and Historical Inquiry, the University of Sydney, Sydney, NSW 2006, Australia; Animal Genomics and Improvement Laboratory, Agricultural Research Service USDA, Beltsville, MD 20705, USA; College of the Environment, Departments of Biology, and Earth & Environmental Sciences, Wesleyan University, Middletown, CT 06459, USA; The Center for Genome Architecture, Department of Molecular and Human Genetics, Baylor College of Medicine, Houston, TX 77030, USA; Center for Theoretical and Biological Physics, Rice University, Houston, TX 77005, USA; Center for Theoretical and Biological Physics, Rice University, Houston, TX 77005, USA; Institute for Biosafety in Plant Biotechnology, Julius Kühn-Institut, Quedlinburg 06484, Germany; Developmental Epigenomics Lab, Garvan Institute of Medical Research, Darlinghurst, NSW, Australia; Developmental Epigenomics Lab, Garvan Institute of Medical Research, Darlinghurst, NSW, Australia; Developmental Epigenomics Lab, Garvan Institute of Medical Research, Darlinghurst, NSW, Australia; Statewide Genomics, New South Wales Health Pathology, Newcastle, NSW 2300, Australia; Vineyard Veterinary Hospital,Vineyard, NSW 2765, Australia; Developmental Epigenomics Lab, Garvan Institute of Medical Research, Darlinghurst, NSW, Australia; Charles Perkins Centre, Faculty of Medical Sciences, University of Sydney, Camperdown, NSW 2006, Australia; The Center for Genome Architecture, Department of Molecular and Human Genetics, Baylor College of Medicine, Houston, TX 77030, USA; Center for Theoretical and Biological Physics, Rice University, Houston, TX 77005, USA; UWA School of Agriculture and Environment, The University of Western Australia, Perth, WA 6009, Australia; Broad Institute of MIT and Harvard, Cambridge, MA 02142, USA

**Keywords:** type specimen, cranium, long-read sequencing, *de novo* genome assembly, biogeography

## Abstract

**Background:**

One difficulty in testing the hypothesis that the Australasian dingo is a functional intermediate between wild wolves and domesticated breed dogs is that there is no reference specimen. Here we link a high-quality *de novo* long-read chromosomal assembly with epigenetic footprints and morphology to describe the Alpine dingo female named Cooinda. It was critical to establish an Alpine dingo reference because this ecotype occurs throughout coastal eastern Australia where the first drawings and descriptions were completed.

**Findings:**

We generated a high-quality chromosome-level reference genome assembly (Canfam_ADS) using a combination of Pacific Bioscience, Oxford Nanopore, 10X Genomics, Bionano, and Hi-C technologies. Compared to the previously published Desert dingo assembly, there are large structural rearrangements on chromosomes 11, 16, 25, and 26. Phylogenetic analyses of chromosomal data from Cooinda the Alpine dingo and 9 previously published *de novo* canine assemblies show dingoes are monophyletic and basal to domestic dogs. Network analyses show that the mitochondrial DNA genome clusters within the southeastern lineage, as expected for an Alpine dingo. Comparison of regulatory regions identified 2 differentially methylated regions within glucagon receptor GCGR and histone deacetylase HDAC4 genes that are unmethylated in the Alpine dingo genome but hypermethylated in the Desert dingo. Morphologic data, comprising geometric morphometric assessment of cranial morphology, place dingo Cooinda within population-level variation for Alpine dingoes. Magnetic resonance imaging of brain tissue shows she had a larger cranial capacity than a similar-sized domestic dog.

**Conclusions:**

These combined data support the hypothesis that the dingo Cooinda fits the spectrum of genetic and morphologic characteristics typical of the Alpine ecotype. We propose that she be considered the archetype specimen for future research investigating the evolutionary history, morphology, physiology, and ecology of dingoes. The female has been taxidermically prepared and is now at the Australian Museum, Sydney.

## Introduction

The most influential book on evolution, Darwin's 1859 *On the Origin of Species* [1], starts with a chapter on domestication to reverse engineer natural selection. Some 9 years later, Darwin [2] expanded his initial thinking into the book *The Variation of Animals and Plants under Domestication*. He hypothesized that the process of domestication proceeded in a stepwise manner first by unconscious selection (wild → tamed), followed by what we now call artificial selection (tamed → domesticated), with the key distinction between these processes being the involvement of humans on mating and reproduction. A gap in our ability to test Darwin's hypothesis has been the identification of a model system with an extant plant or animal that is intermediate between the wild ancestor and the domesticate. Here we explore the overarching hypothesis that the Australasian dingo (*Canis (familiaris) dingo*) is evolutionarily intermediate between the wild wolf (*Canis lupus*) and domestic dogs (*Canis familiaris*) [3]. One alternate hypothesis is that the process of domestication is continual and does not proceed in a stepwise manner [4], instead representing a series of phases reflecting an intensification of the relationship between a wild animal (or plant) and human societies [5].

The taxonomic name of the dingo remains unstable, but it is now clear the Australasian dingo is a distinct evolutionary lineage closely related to domestic dogs [6]. The first European drawing of an animal referred to as a “dingo” appears in White 1790 [7], with a more complete anatomic description appearing in Meyer 1793 [8]. A “large dog” from coastal eastern Australia near Sydney was earlier illustrated by George Stubbs in 1772, based on a recorded description by Joseph Banks from 1770; it is now clear that this animal was a dingo, but the name had not yet been learned from the local Aboriginal people. We follow the precedent that when zoologists disagree over whether a certain population is a subspecies or a full species, the species name may be written in parentheses. Scientists advocating a general lineage species concept consider dingoes to be a distinct species (*Canis dingo*) or a subspecies of domestic dog (*Canis familiaris dingo*) [9–11]. Others advocating a biological species concept [12] consider the dingo to be a breed of dog (*Canis familiaris* breed dingo) due to the interfertility between dingo and domestic dog [11, 13, 14].

Corbett [15] mentioned the possibility of 3 different dingo ecotypes existing in north, central, and southeastern Australia. These are now referred to Tropical, Desert, and Alpine dingoes [16]. Subsequently, Corbett [17] noted that dingo skulls from southeastern Australia (Alpine dingoes) were genuinely different from those of the rest of the country but posited the differences may be due to hybridization with domestic dogs rather than independent lineages. Jones [18] agreed that the southeastern dingoes were distinct and suggested a revaluation of ecotype morphologies to resolve the conundrum.

Analyses of mitochondrial variation in canids from Southeast Asia support the hypothesis that there are distinct dingo lineages [19–22]. Zhang et al. [19] found a strong Bayesian posterior value supporting the separation of Australian dingoes into 2 groups. One is a northwestern group, whereas the other is a southeastern group that clusters with New Guinea Singing dogs (*Canis (familiaris) hallstromi*). Support for 2, or perhaps 3, distinct lineages of dingoes has also come from Y-chromosome and single-nucelotide polymorphism (SNP) chip data [23, 24].

The dog is the first species to be domesticated [25]. They are likely the most frequently kept domestic animal and exhibit exceptional levels of morphologic variation, and many breeds have been developed by strong artificial selection in the past 200 years [26–28]. The Australasian dingo has been proposed to be a functional [29] and evolutionary [6] intermediate between wild wolves and domesticated dogs. Unfortunately, the absence of a dingo holotype reference specimen impedes our ability to definitively determine whether dingoes are a tamed intermediate or a feral canid because we do not have a single reference point that links the scientific name to a specific specimen [30].

**Figure 1:**
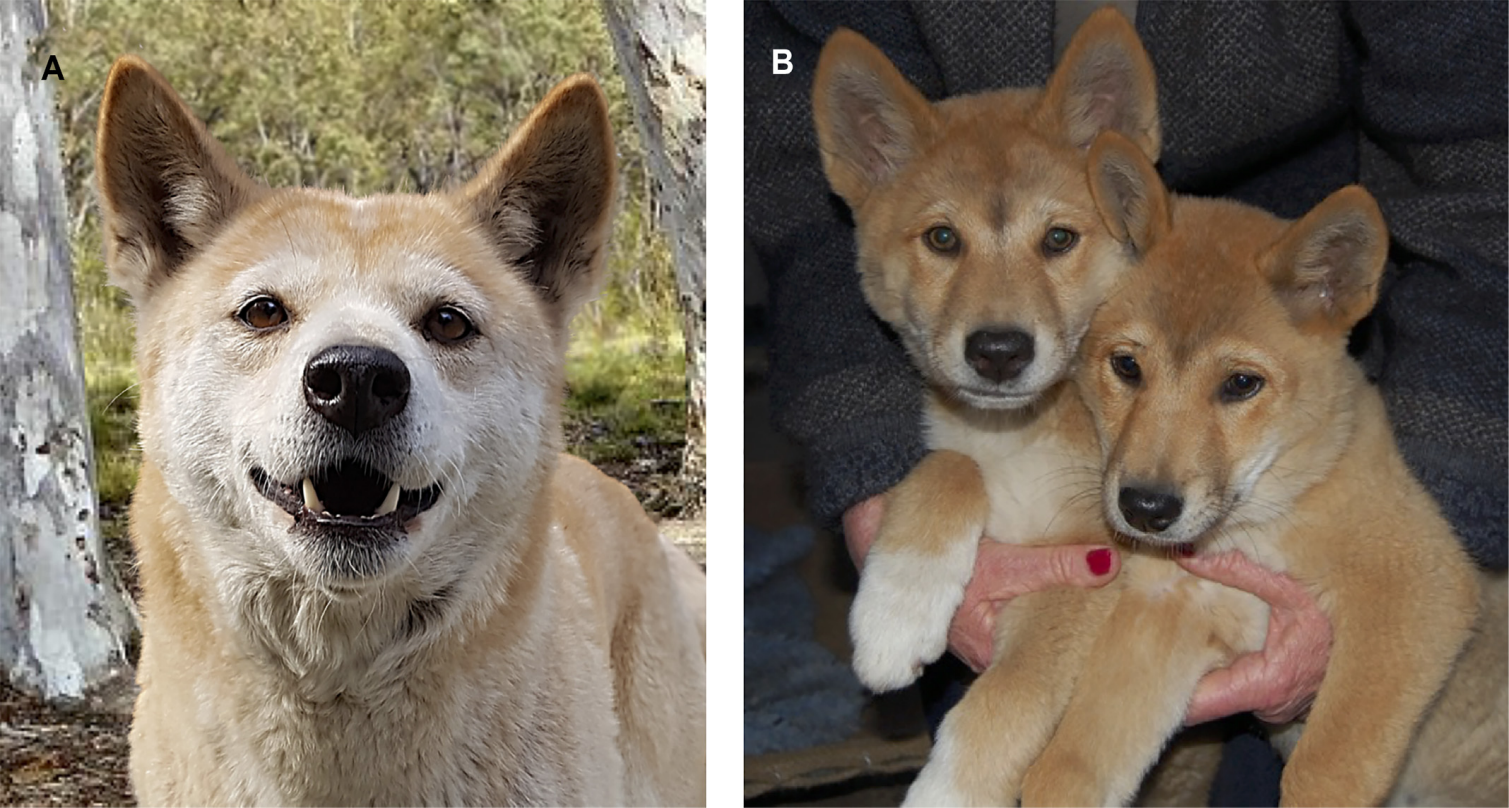
Cooinda the dingo. The genomic and morphological data in this study are based upon a single individual named Cooinda from Dingo Sanctuary Bargo in the southern highland region of New South Wales. Based on her parentage, broad skull, and stocky appearance, the Sanctuary considers her an Alpine dingo. We compare her with other dingoes found in southeastern Australia and with those found in the center and northwest of the continent, including Desert dingo Sandy [6]. (A) Dingo Cooinda as an adult female. (B) Brother Typia (RHS) and Cooinda (LHS) as 8-week-old puppies.

This study aims to link high-resolution long-read *de novo* chromosomal assembly plus mitochondrial DNA sequence and the DNA methylome with morphologic descriptions of head shape and computed tomography of brain data to describe the “archetype” dingo (Fig. 1). This designation will support future comparisons with a reference enabling further characterization of the evolutionary history of the dingo. In this case, we do not propose any formal taxonomic name for the specimen as it is a regional morphotype that is being characterized, but we suggest the principle of having a “type” specimen makes biological sense and will enable the focusing of future research.

## Results

### Chromosome-level genome assembly

#### Workflow

The genome was assembled following a similar pipeline to Field et al. [28] (Supplementary Fig. S1). Briefly, 1,722 contigs were assembled from Pacific Biosciences (PacBio) CLR and Oxford Nanopore (ONT) PromethION sequence data with a total length of 2.38 Gb and N50 length of 12.4 Mb [31]. The contig assembly was then polished for 2 rounds with PacBio reads, correcting ∼5 million bases in the first round and ∼15,000 in the second [32, 33]. The assembled sequence contigs were scaffolded sequentially using 10× linked reads and polished with 10× linked reads [33]. The scaffolded assembly was then super-scaffolded with Bionano and Hi-C proximity ligation. Supplementary Fig. S2 shows the contact matrices generated by aligning the Hi-C data set to the genome assembly after Hi-C scaffolding [34, 35]. To increase the contiguity of the assembly, we used the PacBio and ONT reads to fill gaps, which was then followed by a final round of PacBio read polishing. The gap filling successfully closed 282 gaps, increasing contig N50 to the final figure of 23.1 Mb. A final round of polishing was performed with 10× linked reads. The resulting chromosome-length genome assembly and its gene annotation were deposited to NCBI with accession number GCA_012295265.2.

#### Assembly statistics and completeness

The final assembly had a total length of 2,398,209,015 bp in 477 scaffolds with a scaffold and contig N50 of 64.8 Mb and 23.1 Mb, respectively (Table 1). Chromosome-level scaffolds accounted for 98.4% of the assembly with only 0.9% (21.1 Mb) of all sequences not aligning to a CanFam4.1 chromosome [36].

**Table 1:** Genome assembly and annotation statistics for Alpine dingo (Cooinda) vs. Desert dingo assembly (Sandy)

Statistic	Alpine dingo	Desert dingo
Total sequence length	2,398,209,015	2,349,862,946
Total ungapped length	2,390,794,485	2,349,829,267
Number of contigs	802	228
Contig N50	23,108,747	40,716,615
Contig L50	36	20
Number of scaffolds	477	159
Scaffold N50	64,752,584	64,250,934
Scaffold L50	15	14
Number of gaps	325	69
BUSCO complete (single/duplicate copy)	95.1% (S: 92.7% D:2.4%)	95.3% (S: 92.9% D:2.5%)
BUSCO fragmented	0.8%	0.8%
BUSCO missing	4.1%	3.8%

Evaluation by BUSCO (v5.2.2 [37]) against the Carnivora_odb10 dataset (*n* = 14,502) indicated that 95.1% of the conserved single-copy genes were complete (Table 1, Supplementary Fig. S3A). Only 3 of 13,791 complete (single-copy or duplicated) BUSCO genes were not on the 39 nuclear chromosome scaffolds.

Next, we compared single-copy “Complete” BUSCO genes in Alpine dingo Cooinda and 9 canid genomes [6, 27, 28, 36, 38–41]. Of the 13,722 genes, 13,711 were found in the assembly using BUSCOMP v1.0.1. Only Sandy the Desert Dingo v2.2 (13,715 genes) and China the Basenji v1.2 (13,712 genes) had more.

Additional *k*-mer analysis of the final assembly [42] yielded 97.32% (97.2% in chromosomes) and an overall Q-score estimate of 37.5 (38.4 for chromosomes). No sign of retained haplotigs was evident (Supplementary Fig. S3B).

### Comparison of dingo genomes

**Figure 2:**
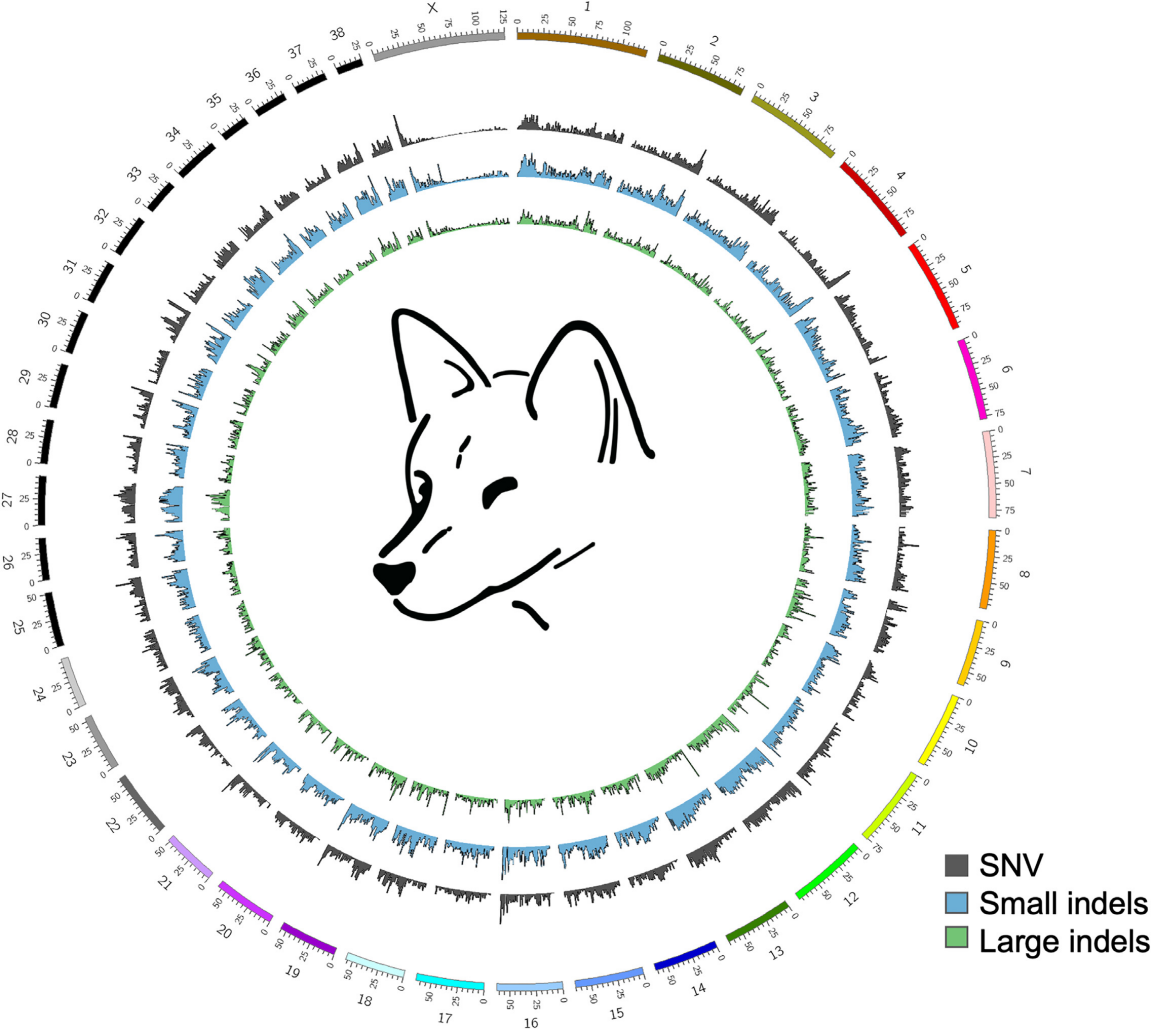
Circos plot comparing Alpine and Desert dingo genomes. Plot compares the 38 autosomes and X chromosome of the Alpine and Desert dingo. The plot shows the low variation on the X chromosome compared to the autosomes.

We generated a Circos plot [43] to represent the single-nucleotide variants (SNVs) and small indel variation between the Alpine and Desert dingo (Fig. 2) using MUMmer4 [44] and sniffles v1.0.11 [45]. In comparison to the autosomes, these plots show low variation on the X chromosome (Fig. 2). To further investigate the low variation, we compared each of the dingoes to CanFam4 (Supplementary Fig. S4, Supplementary Table S1). We then generated a conservative consensus set of structural variants (SVs) by merging PacBio and Nanopore SV calls generated with sniffles [45, 46]. Overall, we found around half the number of SV and small variants calls relative to Desert dingo than to CanFam4 (32,798 vs. 62,524 and 1,729,790 vs. 3,839,712, respectively).

We generated synteny plots using MUMmer plot and GenomeSym [47]. Synteny plots between the dingo genomes show several large-scale chromosomal events. On chromosome 16, there is a 3.45-Mb inverted region and a 0.9-Mb complex rearrangement (Supplementary Fig. S5). This 3.45-Mb inversion does not appear in the wolf or domestic dogs, so we speculate it is unique to the Desert dingo assembly [6]. The inversion overlaps 60 unique ENSEMBL transcripts and was enriched for gene ontology terms of cellular metabolic processes, including glycolysis and glucose metabolism [6]. Also, on chromosome 16, the 0.9-Mb complex rearrangement occurs between 55 and 57 Mb downstream (Supplementary Fig. S5). Additional structural events include small inversions on chromosome 11 and chromosome 25 (Supplementary Fig. S5). On the X chromosome, there appear to be multiple small nonsyntenic regions (Supplementary Fig. S5); however, further examination of these apparent differences is required to establish whether they are true biological differences or assembly artifacts.

In parallel, we used GeMoMa gene predictions [48] to investigate chromosomal-level events. Like the synteny analyses, this approach revealed a large inversion and a disordered region on chromosome 16 as well as smaller inversions on chromosomes 11 and 25. We also found 2 structural events on chromosome 26 (Supplementary Fig. S6) containing mostly short genes that are not perfectly conserved (Supplementary Fig. S5F). A MUMmer4 nucmer alignment plot [44] for chromosome 26 corroborated these events (Supplementary Fig. S6).

The Alpine and Desert dingo both have a single-copy pancreatic amylase gene (AMY2B) on chromosome 6. The Alpine dingo assembly does not include a 6.4-kb-long Long interspersed nuclear element (LINE) that was previously reported in the Desert dingo [6].

### Phylogenetic analyses

**Figure 3:**
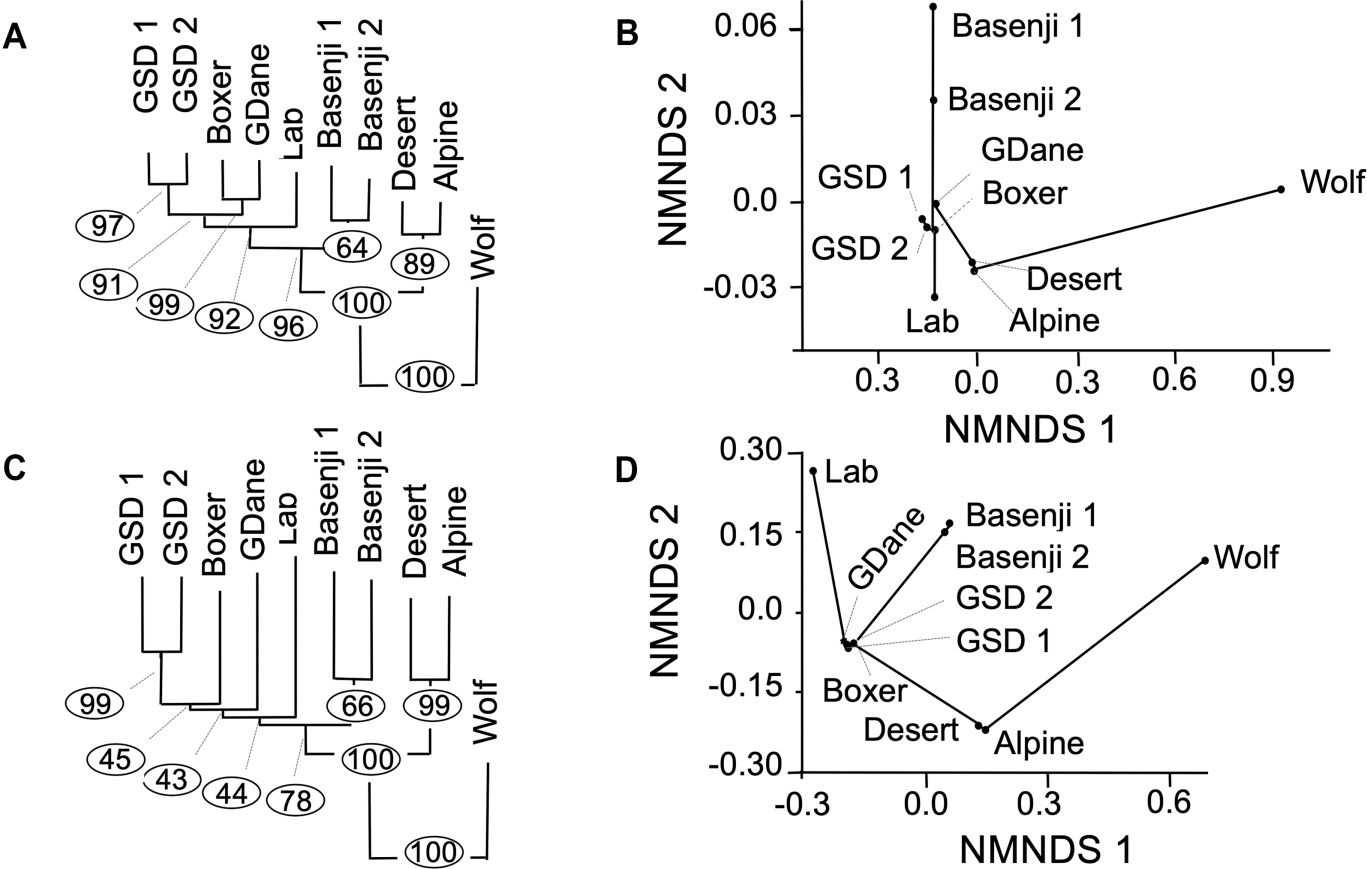
Phylogenetic and ordination analyses of nuclear DNA from SNVs and indels from 10 canines. (A) Phylogenetic tree from SNVs. Branch length proportional to the number of changes and bootstrapping percentage in circles. (B) Ordination analyses from SNVs showing first 2 axes from nonmetric multidimensional scaling (NMDS). (C) Phylogenetic tree from indels. Branch length proportional to the number of changes and bootstrapping percentage in circles. (D) Ordination analyses from indels showing the first 2 axes from nonmetric multidimensional scaling (NMDS). Lab, Labrador; GSD, German Shepherd Dog; GDane, Great Dane; Wolf, Greenland wolf.

All 39 full-length chromosomes in the final assembly were aligned to the corresponding chromosomes in 9 published canine *de novo* genome assemblies [6, 27, 28, 36, 38–41]. SNVs and small indels (deletions and insertions <50 bp) were called using the MUMmer4 call-SNPs module for all possible pairings (Supplementary Table S2). Distance matrices were generated from the intercanid differences in SNVs and indels and then transformed to WA distance [6, 49]. Fig. 3A, C shows the phylogenetic tree from SNVs and indels, respectively. Both figures show strong support for monophyly of dingoes and dogs relative to the wolf. These figures also strongly support the hypothesis that dingoes are the sister group to domestic dogs. Fig. 3B, D shows the ordination analyses from SNVs and indels, respectively. Scores for the taxa calculated from the largest 2 axes (axis 1 and axis 2) describe 75.6% of the variance in SNVs and 73.2% of the variance in indels (Fig. 3B, D).

### Mitochondrial genome

#### Genome assembly workflow

A 46,192-bp contig from the assembly mapped onto the CanFam reference mitochondrial DNA (mtDNA). It constituted a repeat of approximately 2.76 copies of the mtDNA. Following additional polishing and circularization, a final 16,719-bp mtDNA genome was extracted and has been uploaded to GenBank (OP476512).

### Comparison of dingo mtDNA genomes

When the mtDNA genome of Alpine dingo Cooinda is compared with that of Desert dingo, there is a single 10-bp SV in the control region that highlights the repeat number difference. In the former, there are 28 repeats (RCGTACACGT) ACGTACGCGCGT, while in the latter, there are 29. Potentially, the R(G or A) could represent heteroplasmy [50] that may be further studied with single-cell sequencing approaches [51]. Folding this region [52] shows that increasing repeat number increases stem length and overall stability (Supplementary Fig. S7).

**Figure 4:**
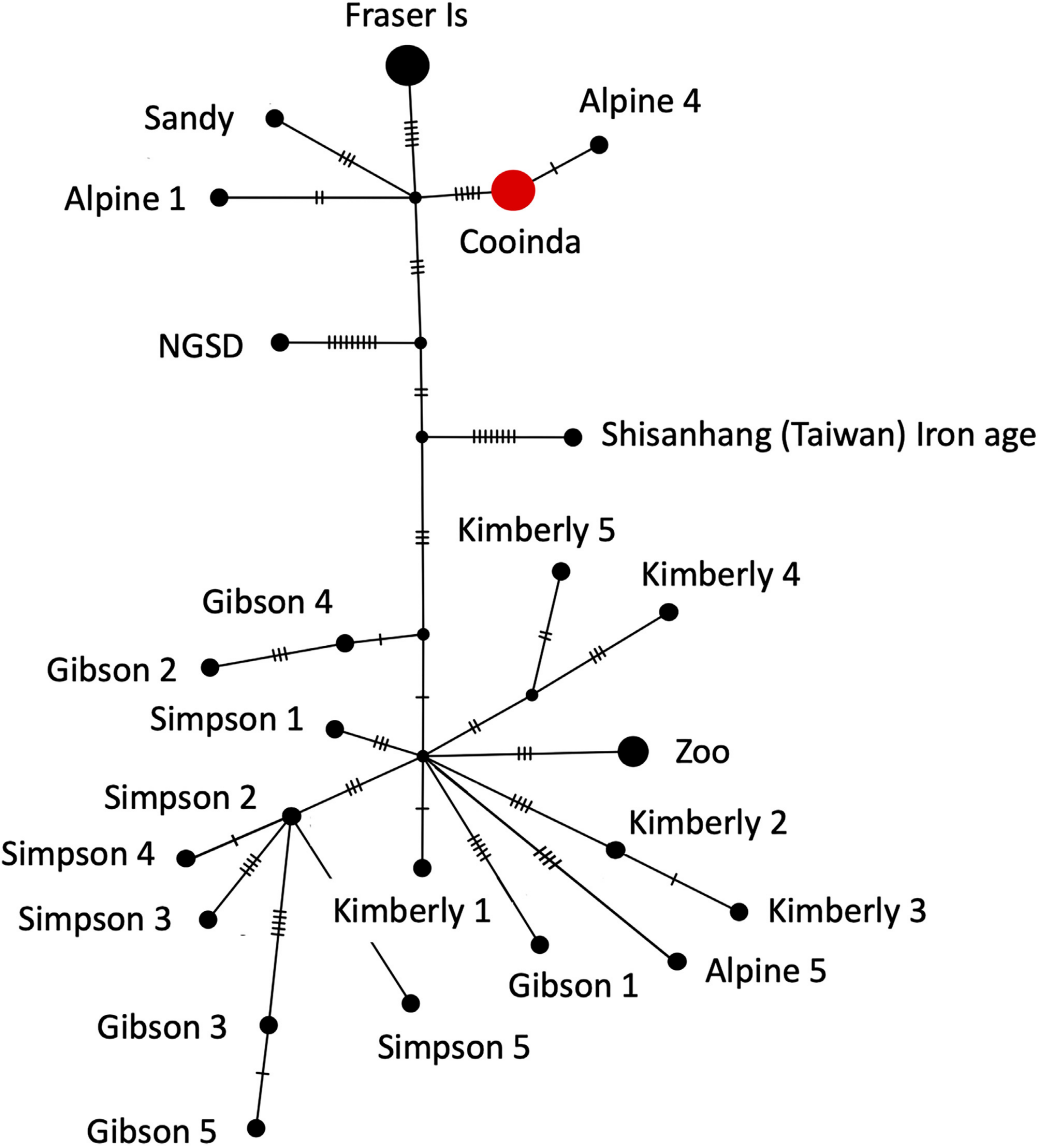
Neighbor-joining network analysis from mtDNA. The size of the circle represents the number of identical sequences and small cross lines the number of SNPs on each branch. The analyses show that dingo Cooinda is in the southeastern clade. Cooinda represents Alpine dingo Cooinda sequenced here, as well as Alpine 2, Alpine 3 [22], MH035670 [55], and Typia [57]. Fraser Is represents the Fraser Island 1–5 samples [22]. Zoo represents 3 dingoes from the New Zealand Zoo [55]. Shisanhang (Taiwan) is one of 2 samples from the region and is considered the root of the network [19].

Next, we conducted a network analysis in Popart [53] to determine whether the mtDNA of dingo Cooinda fell within the previously described dingo southeastern or northwest clade (Fig. 4) [19, 22]. We included dingo mtDNA from 4 previous studies, a New Guinea singing dog and an ancient Iron Age dog from Taiwan [6, 22, 54–56]. There were 89 segregating sites and 32 parsimony informative sites in the dataset. Predictably, there were no differences between the mtDNA genome of Cooinda and that previously published from her brother Typia [54]. Further, as expected, Cooinda and Typia mtDNA clustered with samples that had previously been collected from the Alpine region (Fig. 4). Somewhat unexpectedly, the mtDNA from Sandy the dingo found in the desert [6] did not cluster with dingoes from the northwest clade but was closer to canids in the southeastern clade (Fig. 4). This relationship could imply the introgression of Alpine alleles into the Sandy genome, but further work would be needed to confirm this.

## DNA methylome

To explore the regulatory landscape of dingo Cooinda, we performed whole-genome bisulfite sequencing [58] on genomic DNA extracted from whole blood. In concordance with other adult vertebrates [59, 60], the Cooinda genome displays a typical bimodal DNA methylation pattern. Over 70% of CpG dinucleotides are hypermethylated (levels higher than 80%) and 5% of CpG dinucleotides hypomethylated (methylated at 20% or lower) (Supplementary Fig. S8A).

Next, to determine the number and genomic distribution of putative regulatory regions, we segmented the methylome into unmethylated regions (UMRs) and low-methylated regions (LMRs) using MethylSeekR [61]. UMRs are fully unmethylated and largely coincide with CpG island promoters, whereas LMRs display partial DNA methylation, characteristic of distal regulatory elements such as enhancers in other mammalian models [62]. MethylSeekR analysis identified ∼19,000 UMRs and ∼44,000 LMRs in line with previously reported numbers of promoters and enhancers (e.g., human: ∼18,000–20,000 UMRs and 40,000–70,000 LMRs; mouse: ∼17,000–19,000 UMRs and 55,000–90,000 LMRs) [61, 63] (Supplementary Fig. S8BC).

To establish whether proximal gene regulatory regions in the dingo Cooinda genome display different methylation states in the Desert dingo, we converted Cooinda UMR coordinates from Cooinda to the Desert dingo genome assembly using LiftOver (see Methods). Next, we calculated average DNA methylation at Cooinda UMRs and their corresponding lifted-over regions in the Desert dingo genome. We found 2 UMRs in the Cooinda dingo were hypermethylated in the Desert dingo. These regions overlapped gene bodies of glucagon receptor gene GCGR and histone deacetylase HDAC (Supplementary Fig. S8DE). GCGR is on chromosome 9 and has a single transcript. This transcript is 99.8% identical at the amino acid level between the dingoes. HDAC4 occurs on chromosome 25 and has 12 transcripts, with all 12 transcripts being 100% identical at the amino acid level. Further studies are needed to determine the functional significance of the observed differences in DNA methylation. Altogether, these data provide a genome-wide resource for the putative gene regulatory regions in the Alpine dingo genome, which will be instrumental for future studies.

### Morphology

#### Skull morphometrics

**Figure 5:**
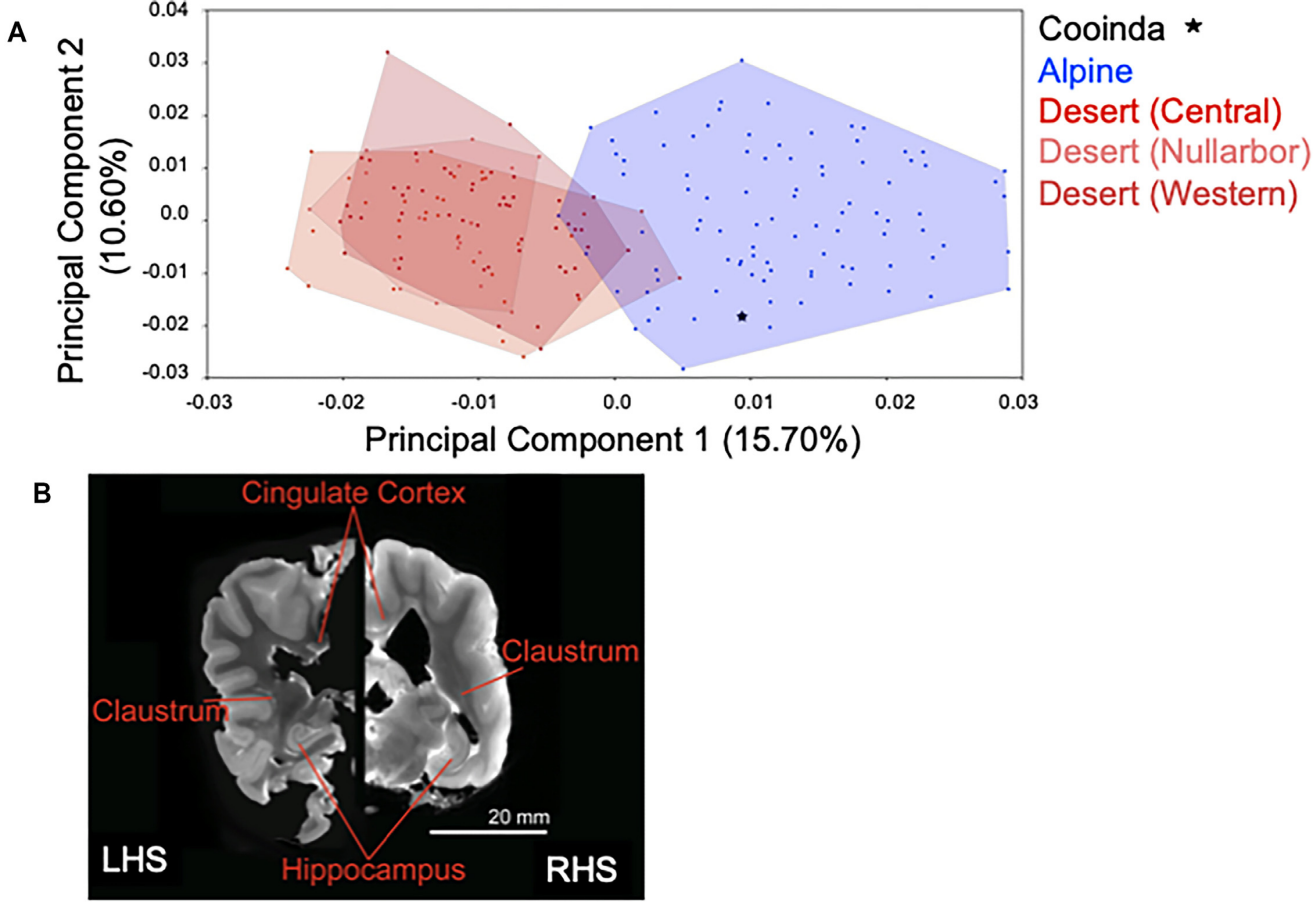
Morphometrics and brain image of Cooinda from the Bargo Dingo Sanctuary, NSW, Australia. (A) Principal component ordination of geometric morphometric cranial shape data indicating Cooinda's position in relation to Alpine and Desert dingoes. Blue represents Alpine dingoes, and the red hues indicate dingoes from different deserts that are broadly overlapping. Dingoes from the Nullarbor overlap most with those from the Alpine region. There is no overlap of dingoes from the Central desert with Alpine dingoes.(B) Brain image, showing a hemispheric comparison of slices generated by magnetic resonance (MR) imaging of Cooinda dingo (CD) and a similar-sized domestic dog (DD).

Cranial morphology (Supplementary Fig. S9A), quantified using 3-dimensional geometric morphometric landmarks, is that of a typical adult female Alpine dingo (Fig. 5). Within the morphospace defined by the principal components explaining the greatest variation between specimens (PC1, PC2), dingo Cooinda's position is clearly within the Alpine cluster (Fig. 5A). Alpine and Desert dingoes are most clearly differentiated from one another along PC1 (15.70%), for which increasing values describe crania with relatively shorter and broader rostra, shallower orbitals with broader zygomatic arches at the glenoid fossa, prominent and anteriorly positioned frontals, a higher cranial vault, and prominent sagittal cresting tending to terminate in a high, posteriorly positioned occiput (inion). Positive values along PC2 (10.60%) mainly denote relatively gracile crania with posteriorly angled frontals, poorly developed sagittal cresting, downward-sloping posterior calvarium, and a low occipital termination. The sampled Alpine and Desert groups exhibit a near-identical range of PC2 values. As the development of the sagittal cresting, calvarium shape and occipital prominence are related to age and sex, with these traits tending to be more robust and well developed in males and older dingoes [64], the shared PC2 values across Alpine and Desert groups likely reflect related demographic variation within the respective populations. Within each population (Alpine, Central Desert, Western Desert), males and females overlapped in their position along PC2 (Supplementary Fig. S9), indicating an absence of strong dimorphism associated with the major axes of shape variance. Despite considerable overlap, PC2 scores tended to be lower in females compared to males in the Alpine and Western Desert populations (see Supplementary Fig. S9, Supplementary Table S3).

The regression of cranial shape (Procrustes shape variables) on log centroid size (Procrustes shape variables ∼log(centroid size)) revealed that size contributed significantly to shape variance in the sample (3.91% variance, *P* < 0.001). Size was found to have a nonsignificant effect on the morphologic trajectory described by PC1, which separates Alpine and Desert dingo populations (Fig. 1C), with only 1.23% of related shape change predicted by centroid size (*P* = 0.124). Conversely, size predicted 19.88% of shape change associated with PC2 (*P* < 0.0001). Alpine and Desert dingo populations share overlapping scores along PC2, and variation along this axis reflects intrapopulation variability in demographic makeup (age, sex) that should be expected within a natural population. As such, size differences play very little to no role in determining Cooinda's morphologic relationship to Desert dingoes but are important to her position in the Alpine group (Supplementary Fig. S10B, C). The low proportion of variation captured in each principal component is a previously noted feature of the dingo cranial landmark dataset [65] and is unrelated to allometry.

#### Brain imaging

To supplement the morphologic data, we quantified brain size. Using a thresholding approach, we used the software 3D Slicer [66] to segment the whole brain as the region of interest. Despite the canids being of very similar size, the dingo brain (75.25 cm^3^) was 20% larger than the dog brain (59.53 cm^3^) (Fig. 5B).

## Discussion

Domestication has received much attention from diverse fields, reflecting the complexity of the process and variation in its duration and intensity [5]. A notable gap in our understanding of the principles of domestication has been the identification of a model system to test Darwin's 2-step predictions [2]. Here we provide the necessary groundwork to explore the potential for dingoes to be a functional and evolutionary intermediate between wild wolves and domestic dogs. One alternate hypothesis is that the process of domestication does not proceed in a stepwise manner [4] but is a continual process that represents an intensification of the relationship between a wild species and humans [5].

In this study, we compare our high-quality chromosome-level *de novo* assembly of the dingo Cooinda genome with that of the Desert dingo [6], 7 domestic dogs [27, 28, 36, 38–40] and the Greenland wolf [41]. Relative to the wolf and the domestic breeds, the Australasian dingo ecotypes are monophyletic. Future studies may include ancient dingo and Southeast Asian specimens [3], the New Guinea singing dog [4], and Chinese indigenous dogs [4]. Ancient specimens have potential to give insight into the evolutionary history of dingoes [3] and further instruct the influence of domestic dog admixture [17]. New Guinea singing dog may be the sister group to a monophyletic dingo lineage or perhaps more closely related to the Alpine ecotype, as suggested by the mtDNA network analyses [19] and cranial shape studies [65]. Inclusion of Chinese indigenous dogs will facilitate determination of the relationships among crown domestic dog breeds [4] and thereby facilitate determination of the divergence date of dingoes and modern dogs.

Multiple large-scale chromosomal inversions occur between the 2 dingo assemblies. There are 2 large rearrangements on chromosome 16 and likely structural events on chromosomes 11, 25, and 26 (Supplementary Figs. S7, S8). It is also possible that there are multiple small inversions on the X chromosome. It is important to determine the frequency of these events and whether breakpoints affect any regulatory regions or protein-coding genes. Inversions may maintain locally adapted ecotypes, while breakpoints may disrupt regulatory regions or protein-coding genes. Hager et al. [67] discovered a 41-megabase chromosomal inversion that characterized defining traits of deer mice (*Peromyscus maniculatus*) and implicated divergent selection in maintaining distinct ecotypes in the wild despite high levels of gene flow. An inversion disrupting FAM134b has been associated with sensory neuropathy in Border Collie dogs [68].

There is a single copy of AMY2B in both dingo genomes; however, they differ by a 6.4-kb retrotransposon insertion present in the Desert dingo. As the retrotransposon is absent in the Greenland wolf and Alpine dingo, it would seem likely that the retrotransposon has inserted into the Desert dingo and domestic dog lineages independently. LINE elements can generate duplications through an RNA intermediate and have been associated with amylase expansions in a range of species from humans to mice and rats to dogs [69, 70]. A 1.3-kb canid-specific LINE element in domestic dogs is associated with each amylase copy [70]. This expansion is predicted to increase the ability to digest starch [6, 71]. Field et al. [28] compared the influence of *AMY2B* copy number on the microbiomes of dingoes and German Shepherd dogs. They observed distinct and reproducible differences that they hypothesized may influence feeding behaviors. Further studies on *AMY2B* may be fruitful as copy number may be an ecologically relevant mechanism to establish the role of a canid in the ecosystem.

Both dingo ecotypes exhibited low variation on the X chromosome, although it could be argued that variation along the chromosome is not uniform (Fig. 2). Theoretical models predict that genes on the X chromosome can have unusual patterns of evolution due to hemizygosity in males. Sex chromosomes are predicted to exhibit reduced diversity and greater divergence between species and populations compared to autosomes due to differences in the efficacy of selection and drift in these regions [72, 73]. In canids, Plassais et al. [74] show genetic variation in 3 genes on the X chromosome is strongly associated with body size. Further studies of genetic variation of genes on the X chromosome within and between ecotypes are likely informative.

We integrate the mtDNA genome assembly data with that previously collected from 29 canids in Australasia [6, 22, 54–56]. The mitochondrial genome has been used to infer historical events in various species, including canids, but the D-loop region has been difficult to align. Here we show that the region can be folded to increase structural stability with repeat number (Supplementary Fig. S8A, B). We found twenty-eight 10-bp repeats in dingo Cooinda compared to 29 in the Desert dingo. The function of the proposed structures is unknown. Still, folding the region into an extended repeat-dependent stem is expected to decrease the time the DNA in the D-loop is single-stranded during replication. More speculatively, the structure may have a regulatory function that influences mitochondrial bioenergetics and the evolution of mtDNA [75]. Björnerfeldt et al. [76] found that domestic dogs have accumulated nonsynonymous changes in mitochondrial genes at a rate faster than wolves, implying a relaxation of selective constraint during domestication.

Phylogenetic and network analyses show that dingo Cooinda has the dingo southeastern Australian mtDNA type of the canine A1b4 subhaplogroup. This southeastern type has been proposed to originate in southern China and includes dogs from Papua New Guinea [19, 22]. Based on mtDNA data, Zhang et al. [19] propose that the time to most recent common ancestor (TMRCA) for most dingoes dates to 6,844 years ago (8,048–5,609 years ago). This estimate is about 3,000 years older than the first known fossil record [77], suggesting that at least 2 dingo mtDNA haplotypes colonized Australia or older fossil records of dingoes in Australia have yet to be found.

Next, we compare the regulatory landscape of Cooinda dingo with that previously published for the Desert dingo. In comparison to the Alpine dingo, the glucagon receptor gene GCGR and HDAC4 are hypermethylated in the Desert dingo, suggesting the potential for dietary or immune differences between ecotypes. Highly methylated gene promoters often indicate a transcriptionally repressed state, while unmethylated gene promoters specify a permissive state [78]. Field et al. [6] previously proposed differences in the feeding behavior of dingoes and wild dogs linked to their *AMY2B* copy number. GCGR is activated by glucagon and initiates a signal transduction pathway that begins with the activation of adenylate cyclase, which in turn produces cyclic AMP. Glucagon is considered the main catabolic hormone of the body and is central to regulating blood glucose and glucose homeostasis [79]. In mice, glucagon has anti-inflammatory properties [80]. HDAC4 is a member of the ubiquitously important family of epigenetic modifier enzymes and has been implicated in processes related to the formation and function of the central nervous system and metabolism. HDAC4 acts as a regulator of pattern recognition receptor signaling and is involved in regulating innate immune response [81]. In humans, mutations in HDAC4 have been linked with eating disorders [82]. Overlapping conserved Nanopore/PacBio structural variants with these genes identified no variants within GCGR and a single 35-bp intronic insertion in HDAC4. The functional impact (if any) of this insertion is unknown.

Dingo Cooinda's cranial morphology is consistent with the Alpine ecotype from the 20th century. As the first cranial morphologic assessment of an Alpine dingo considered to be “pure” by genomic verification, this result is significant in that it suggests that the phenotypic distinctiveness of Alpine dingoes from Desert dingoes is not exclusively the result of recent domestic dog ancestry. Dog admixture has been the predominant explanation given [83] primarily based on the fact that such ancestry is relatively enriched in the southeast region of Australia compared to the north and west [84, 85]. An alternative explanation is that the Alpine and Desert dingoes represent distinct evolutionary lineages. Koungoulos [65] suggested that the cranial shape of Alpine and other southeastern dingoes shares broad similarities with that of New Guinea singing dogs and is distinct from the more widespread northwestern lineage [22]. However, these 2 scenarios are not mutually exclusive. Most introgression likely occurs when a female dingo mates with a male domestic dog. In such cases, extensive backcrossing will not exclude the domestic dog Y. Therefore, examining the Y chromosome of males shown to be pure with the current battery of nuclear-encoded microsatellites will illuminate genetic history. A combination of direct radiocarbon dating, genetic sequencing, and morphometric assessment for subfossil material will provide a more confident picture of the nature of change or continuity between ancient and modern Alpine dingoes.

Finally, we supplement our morphologic data with magnetic resonance and computed tomography data of Alpine dingo Cooinda's brain. Her brain was 20% larger than the similarly sized domestic dog, which is consistent with the hypothesis that she was tamed but not domesticated [3] (Fig. 1C). Our brain imaging data are also compatible with prior comparisons that have used endocranial volume as a proxy for brain size, examining a small sample of dingoes (see Geiger et al. [86]) compared to wolves and domestic, basal, and archaeological dogs [3]. Endocranial volume in a mixed sample of domestic dogs was shown to be around 30 cm^3^ smaller than in wolves and jackals [87, 88], which is greater than the 15.7-cm^3^ difference between the brains of Cooinda and the domestic dog sampled here. Similarly, brain mass has been shown to be 28.8% smaller in a broad sample (>400) of domestic dogs as compared to wolves [87, 89], which also places the 20% difference between Cooinda and the domestic dog as less pronounced than is seen for comparisons with the wild counterpart (wolf). Brain size reductions are common among domesticated animals compared to their wild counterparts, having been observed across many species, including sheep, pigs, cats, and dogs [87, 90]. Smaller-sized brains, especially size reductions in regions of the forebrain involved in the fight-or-flight response, have been associated with tameness and reductions in fear-based response among domestic animals compared to wild animals [91]. These changes have also been linked to potential reductions in cognitive processing requirements associated with inhabiting anthropogenic environments with lower complexity [92, 93]. Moreover, brain size reductions appear to persist where domestic animals have reentered a wild environment and exist as feralized animals, at least under certain circumstances [94–96], suggesting that prolonged past exposure to the human niche may be detectable in brain traits. An alternative hypothesis is that differences in brain size are due to environmental adaptation or perhaps Cooinda was an anomaly. Examination of brain size may represent a fruitful pathway for further investigation determining the status of the dingo as a potential feralized animal.

There are at least 3 possible explanations supporting the existence of 2 dingo ecotypes (Alpine and Desert). The first is they are ancient Asian lineages that have come into sympatry in Australia. One alternate hypothesis is that a single lineage spread through Southeast Asia and then diverged in Australia. There are no major geographical divides in continental Australia, suggesting any differences may reside at the level of biological interactions or are influenced by climate. In the former case, 1 possibility is that 1 or more inversions may maintain the ecotypes [67]. An intriguing alternate hypothesis is that responses to parasites or venomous animals may occur if there are genetic differences in the responses of the ecotypes. In Nigeria, population genomic analyses of 19 indigenous dogs identified 50 positively selected genes, including those linked with immunity, that likely involve adaptations to local conditions [97]. Experimentally, it has been shown that adaptation to different parasites or snakes can influence the invasion success of three-spined sticklebacks (*Gasterosteus aculeatus*) and may represent a barrier to gene flow, even between closely related connected populations [98]. In Australia, various parasites and venomous animals have broadly similar distributions to the Alpine ecotype, such as the paralysis tick (*Ixodes holocyclus*) and the red-bellied black snake (*Pseudechis porphyriacus*) [99].

## Conclusions

Here we characterize dingo Cooinda and propose that she be considered the archetype for Australasian dingoes. Characterizing an archetype opens potential for testing Darwin's [2] 2-step model of domestication as an alternative to the hypothesis that domestication represents a continuum [5]. Under the scenario that the dingo has been unconsciously selected, we predict genomic signatures of tameness, as an outcome of unconscious selection [100–102]. Morphologically, we predict lowest shape variation in the rostrum and facial skeleton in the wolf (natural selection), intermediate in the dingo (unconscious selection), and highest in domestic breeds (artificial selection) (i.e., rank order: wolf < dingo < modern breeds). Wild populations are more likely to show a narrow range of shape variation about a fitness optimum, whereas changed environmental conditions could support and promote the survival of forms that are farther from the adaptive peak. This is evidenced by earlier research that has shown cranial morphologic variation in domestic dogs exceeds that exhibited by the order Carnivora [26]. In terms of brain size, we predict the magnitude of relative brain size difference will be greater between dingoes and modern breeds than between wolves and dingoes (i.e., rank order: wolf > dingo >> modern breeds). Brain size reduction is pronounced in artificial selection and associated with the lack of fear avoidance behavior in domesticates [103]. Dingoes do not show domesticate-level reductions in “fight-or-flight” response [29], and our initial data appear to be at least consistent with this based on the relative brain volume we report.

## Methods

### Sampling: Cooinda the dingo

In selecting an animal for the project, it was considered essential to select an individual that represented the Alpine ecotype, which is found around Sydney, New South Wales (NSW). The individual selected was bred at the Dingo Sanctuary Bargo, NSW, approximately 100 km west of Sydney, and has been included in multiple previous studies [6, 29]. Cooinda is the litter sister to Typia, from whom short-read data had previously been obtained [54]. Cooinda's parents (Mirri Mirri and Maka), her brothers Typia and Gunya, and her were all ginger in color and determined to be pure by microsatellite testing [104]. Mirri Mirri and Maka were independently found in the Alpine region of New South Wales.

An aim of the study is to link genetic and morphologic variation, so we provide a brief description of her here. As is typical of Alpine dingoes, Cooinda was stocky in appearance with a broad skull and prominent eyes. She was light ginger in color, with dark brown eyes with white paws and chest (Fig. 1A, B). Her double coat was not oily like many modern breed dogs and did not have a dog-like odor when wet. She had a pointed muzzle with a broad skull and hooded erect ears. She could turn her neck 180 degrees in any direction. She had lean muscular legs with a long bottle-shaped bushy tail. She weighed 22 kg and stood 46 cm at the withers. She did not have dewclaws and came into estrus annually. Dingo Cooinda had a loud and clear howl and did not have a modern dog bark [105]. Cooinda died in 2019 at 10 years of age.

### Chromosome-level genome assembly

#### DNA extraction and sequencing

Genomic DNA for the Pacific Bioscience Single Molecule Real-Time (PacBio) sequencing was prepared from 2 mL of fresh blood using the genomic-tip 100/G kit (Qiagen, Hilden, Germany). This was performed with additional RNase (Astral Scientific, Taren Point, Australia) and proteinase K (NEB, Ipswich, MA, USA) treatment following manufacturers' instructions. Isolated genomic DNA (gDNA) was further purified using AMPure XP beads (Beckman Coulter, Brea, CA, USA) to eliminate sequencing inhibitors. DNA purity was calculated using a Nanodrop spectrophotometer (Thermo Fisher Scientific, Waltham, MA, USA). Molecular integrity was assessed by pulse-field gel electrophoresis using the PippinPulse (Sage Science, Beverly, MA, USA) with a 0.75% KBB gel (Invitrogen, Waltham, MA, USA) 1-kb Extension DNA ladder, and 150 ng DNA on the 9-hour 10- to 48-kb (80 V) program. PacBiobell libraries with a 20-kb insert size were CLR sequenced on Sequel I machines with 2.0 chemistry. Sequencing included 18 PacBio cells with a total polymerase read length of 94.25 Gb.

DNA for ONT sequencing DNA (1 µg) was prepared for ONT sequencing using the 1-dimensional gDNA ligation kit (SQK-LSK109, ONT) according to the standard protocol. Long fragment buffer was used for the final elution to exclude fragments shorter than 1,000 bp. In total, 119 ng adapted DNA was loaded onto a FLO-PRO002 PromethION flow cell and run on an ONT PromethION sequencing device (RRID:SCR_017987) using MinKNOW (18.08.2) with a MinKNOW core (v1. 14.2). Base-calling was performed after sequencing with the GPU-enabled guppy basecaller (v3.0.3) using the PromethION high-accuracy flip-flop model with config “dna_r9.4.1_450bps_hac.cfg.”

For the 10X Genomics Chromium sequencing, DNA was prepared following the protocol described above for PacBio sequencing. A 10X GEM library was barcoded from high-molecular-weight (HMW) DNA according to the manufacturer's recommended protocols. The protocol used was the Chromium Genome Reagent Kits v2 (Document # CG00043 revision B). Quality control was performed using LabChip GX (PerkinElmer, Waltham, MA, USA) and Qubit 2.0 Flurometer (Life Technologies, Carlsbad, CA, USA). The library was run on a single lane of a v2 patterned flowcell. Sequencing was performed in a 150-bp paired-end sequencing mode on a single lane on the Illumina HiSeq X Ten platform with a version 2 patterned flowcell.

For the Bionano optical mapping, HMW DNA was isolated from fresh blood (stored at 4°C) using the Bionano Prep Blood DNA Isolation Protocol following [28]. HMW DNA (∼190 ng/µL) was labeled (BNG, Part #20351) at DLE-1 recognition sites, following the Bionano Prep Direct Label and Stain Protocol (BNG, Document #30206 revision C). Labeled DNA was loaded directly onto Bionano Saphyr Chips (BNG, Part #20319), without further fragmentation or amplification, and imaged using a Saphyr instrument to generate single-molecule optical maps. Multiple cycles were performed to reach an average raw genome depth of coverage of 180×.

For the Hi-C sequencing, the assembly was scaffolded to chromosome length by the DNA Zoo following the methodology described here: www.dnazoo.org/methods. Briefly, an *in situ* Hi-C library was prepared [106] from a blood sample of the same female and sequenced to 29× coverage (assuming a 2.6 Gb genome size).

#### Workflow

For the initial assembly, the PacBio and ONT reads were corrected and assembled with the Canu assembler (RRID:SCR_015880; v1.8.0) [31] with the command “canu correctedErrorRate = 0.105 corMhapSensitivity = normal corOutCoverage = 100 -p Cooinda -d assembly genomesize = 2.3 g -pacbio-raw Cooinda_PacBio_ONT_combined.fasta.” The resulting contigs were polished with 2 rounds of the Arrow pipeline, each consisting of aligning the raw PacBio reads to the assembly with pbmm2 (https://github.com/PacificBiosciences/pbmm2) and correcting the sequencing errors using gcpp [32].

The Arrow-polished PacBio/ONT assembly was scaffolded using Alpine dingo 10X linked reads as in ARCS [107]. The 10X data were aligned using the linked-read analysis software provided by 10X Genomics, Long Ranger, v2.1.6 [108]. Misaligned reads and reads not mapping to contig ends were removed, and all possible connections between contigs were computed keeping best reciprocal connections. Finally, contig sequences were joined, spaced by 10 kb with stretches of Ns, and, if required, reverse complemented.

To further improve the assembly, another round of polishing was performed by aligning the Illumina short reads from the 10X Chromium sequencing to the assembly using minimap2 [109] (v2.16) and correcting the sequencing errors using Racon (RRID:SCR_017642; v1.3.3) [110].

The Hi-C data were processed using Juicer (RRID:SCR_017226) [111] and used as input into the 3-dimensional DNA pipeline [112] to produce a candidate chromosome-length genome assembly. We performed additional curation of the scaffolds using Juicebox Assembly Tools [113].

After scaffolding and correction, all raw PacBio and ONT reads were separately aligned to the assembly with Minimap2 (v2.16) (-ax map-pb/map-ont) [109]. The combined alignments were used by PBJelly (pbsuite v.15.8.24) [114] for 1 round of gap filling.

Following scaffolding, another round of polishing was done to further improve the assembly. Polishing was performed by aligning the Illumina short reads from the Chromium sequencing to the assembly using Long Ranger v2.2.2 and correcting the SNVs and indels using Pilon (RRID:SCR_014731) [33].

The Pilon-polished genome underwent a final scaffold cleanup using Diploidocus as described in Edwards et al. [27] to generate a high-quality core assembly, remove low-coverage artifacts and haplotig sequences, and filter any remaining vector/adapter contamination. This reduced the final number of scaffolds to 632 (780 contigs), including the mtDNA.

Assembly completeness was evaluated using BUSCO v5.2.2 [37] short mode against the Carnivora_ob10 dataset (*n* = 14,502) implementing BLAST+ v2.11.0 [115], HMMer v3.3 [116], and Metaeuk v20200908 [117]. “Complete” BUSCO genes with available sequences were compiled across Alpine dingo Cooinda and 9 canid genomes (Desert dingo [6], 2 Basenjis [China and Wags] [27], 2 German Shepherd dogs [Nala and Mischa] [28, 36], Great Dane [38], Labrador [39], Dog10K Boxer [40], and Greenland wolf [41]) using BUSCOMP v1.0.1. Additional *k*-mer-based assembly completeness and quality evaluations were performed using Merqury v21.3 [42] from the 10X reads.

#### Chromosome mapping and variation

Chromosome mapping was completed in 2019 using the CanFam v3.1 reference genome downloaded from Ensembl (GCF_000002285.3 [118]). Full-length chromosomes were renamed with a CANFAMCHR prefix and used for reference mapping. The final Cooinda Alpine dingo genome assembly was mapped onto the CanFam3.1 reference genome using Minimap2 v2.16 [109] (-x asm5 –secondary = no –cs) to generate PAF output. Scaffolds were assigned to CanFam3.1 chromosomes using PAFScaff v0.2.0 [119] based on Minimap2-aligned assembly scaffold coverage against the reference chromosomes. Scaffolds were assigned to the chromosome with highest total coverage. Scaffolds failing to map onto a chromosome were rated as “Unplaced.”

#### Comparison of Alpine and Desert dingo genomes

To investigate the variation between the dingo ecotypes, we used Circos [43]. Circos uses a circular ideogram layout to facilitate the display of relationships between the genomes using ribbons, which encode the position and number of SNVs, small indels, and large indels for each of the 38 autosomes and the X chromosome. SNV and indel numbers were calculated using MUMmer4 “show-snp” script following pairwise alignments [44] (v4.0.0 beta 2).

Synteny plot between the Alpine and published Desert dingo assembly [6] was conducted using GenomeSyn [47]. With GenomeSyn, the position of the genome is indicated by a black horizontal ruler with tick marks. Syntenic blocks between the genomes are displayed as light gray regions, with white illustrating nonsyntenic regions. Inversions are represented by red-brown curves.

We used GeMoMa v1.6.2beta [48] to further investigate whole chromosomal events. Here we mapped genes onto the Alpine dingo assembly following previously described protocols [28]. Subsequently, we checked the synteny of the genes in the reference genome and the target genome using the GeMoMa module SynthenyChecker. This module uses the GeMoMa annotation with information for the reference gene and alternative to determine the best homolog of each transcript. Comparing the order of genes in the reference and the target genome, it allows to determine breakpoints of chromosomal events.

#### Phylogenetic analyses

All 39 full-length chromosomes in the final assembly were aligned to the corresponding chromosomes in 9 published canine *de novo* genome assemblies (Desert dingo [6], 2 basenjis [China and Wags] [27], 2 German Shepherd dogs [Nala and Mischa] [28, 36], Great Dane [38], Labrador [39], Dog10K Boxer [40], and Greenland wolf [41]) using MUMmer4 [44]. SNVs and small indels (deletions and insertions <50 bp) were called using MUMmer4 call-SNPs module for all possible pairings (Supplementary Table S2). Copy number variation (CNV) and SVs were also called using svmu (v0.2) [120], but these were not included in the phylogeny. SNVs and indels were analyzed separately. Distance matrices were generated from the intercanid differences in SNVs and indels and then transformed to WA distance [49]. Glazko et al. [49] report WA has better phylogenetic properties against normalization of genome sizes than other coefficients.

Phylogenetic analyses using maximum parsimony were generated from the R-package “phangorn” version 2.8.1 [121]. The analyses were run as unrooted networks to test the hypothesis that the wolf was the out-group. To test the stability of the nodes, a Bayesian bootstrap was applied to the original distance matrix using the program bayesian_bootstrap (github.com/lmc2179/bayesian_bootstrap), and the phylogenetic analysis was recalculated. This process was iterated 500,000 times. The consensus phylogenetic trees were rooted on the branch leading to wolf, and the values indicate the percentage of times that a node occurred. The y-axis and branch lengths were rescaled to the original number of differences in SNVs and indels among the taxa. The retention index that measures the fit of the network to the distance matrix exceeded 94% for all 500,000 trees of SNVs and indels.

Nonmetric multidimensional scaling (NMDS) was calculated from the distance matrices and scores for the taxa calculated from the largest 2 axes. Minimum spanning trees were calculated among the scores in NMDS space. NMDS and minimum spanning trees were calculated in Past 4.04 [122].

### Mitochondrial genome

#### Genome assembly workflow

A 46,192-bp contig from the assembly mapped onto the CanFam reference mtDNA (NC_002008.4), constituting a repeat of approximately 2.76 copies of the mtDNA. The CanFam mtDNA was mapped onto this contig using GABLAM v2.30 [123], and full-length mtDNA copy with the highest similarity to CanFam mtDNA was extracted along with 8 kb each side. PacBio reads were mapped onto this mtDNA contig using minimap2 v2.22 [109] and 10X linked reads mapped using BWA v0.7.17 [124] for polishing with HyPo v1.0.3 [125] (32.7 kb assembly size at 673× coverage). The CanFam mtDNA was remapped onto the polished assembly using GABLAM v2.30.5 [123] and a 16,719-bp sequence extracted, starting at position 1 of the CanFam sequence. The mtDNA was annotated with the MITOS2 server [126] for submission to NCBI GenBank (accession: OP476512).

#### Comparison of dingo mtDNA genomes

The mtDNA genome of Alpine dingo Cooinda was compared with the Desert dingo [6]. Direct observation of the D-loop region in the 2 dingoes suggested there was a 10-bp repeat, and the canids differed in the number of repeats. Imperfect tandem repeats have previously been reported in canids [50]. The D-loop region in Alpine dingo Cooinda was folded using the program mfold [52] to determine ay underlying structures.

To test whether the mtDNA from dingo Cooinda fell within the previously described SE clade, we compared the assembly with 33 other canids, including dogs from New Guinea and Taiwan [6, 22, 54, 55]. In this case, multiple large gaps were in some of the ancient samples, so the initial assembly was modified based on the predicted secondary structure folding. A interneighbor-joining network analysis with  α = 0.5 was completed in Popart [53]. A limitation of this analysis is that large sections of multiple mtDNAs were unknown, so it was not possible to distinguish deletions from missing data. Understanding these differences may be biologically important, particularly if the predicted folding of the D-loop region is biologically significant.

### DNA methylome

#### MethylC-seq library preparation

Genomic DNA was extracted from whole blood using the DNeasy Blood & Tissue kit (Qiagen). MethylC-seq library preparation was performed as described previously [127]. Briefly, 1 μg gDNA was sonicated to an average size of 300 bp using a Covaris sonicator. Sonicated DNA was then purified, end-repaired, and 3′-adenylated, followed by the ligation of methylated Illumina TruSeq sequencing adapters. Library amplification was performed with KAPA HiFi HotStart Uracil+ DNA polymerase (Millenium Science Pty Ltd, Mulgrave, VIC, Australia).

#### MethylC-seq data analysis

The methylome library was sequenced on the Illumina HiSeq X platform (150 bp, PE), generating 377 million reads. Sequenced reads in fastq format were trimmed using the Trimmomatic software (ILLUMINACLIP:adapter.fa:2:30:10 SLIDINGWINDOW:5:20 LEADING:3 TRAILING:3 MINLEN:50). Trimmed reads were mapped (GCA_012295265.2_UNSW_AlpineDingo_1.0_genomic.fna genome reference, containing the lambda genome as chrLambda) using WALT with the following settings: -m 10 -t 24 -N 10 000 000 -L 2000. Mapped reads in SAM format were converted to BAM format; BAM files were sorted and indexed using SAMtools. Duplicate reads were removed using Picard Tools v2.3.0. Genotype and methylation bias correction were performed using MethylDackel (MethylDackel extract dingo_lambda.fasta $input_bam -o $output –mergeContext –minOppositeDepth 5 –maxVariantFrac 0.5 –OT 10 140,10 140 –OB 10 140,10 140). The numbers of methylated and unmethylated calls at each genomic CpG position were determined using MethylDackel (MethylDackel extract dingo_lambda.fasta $input_bam -o output –mergeContext). Segmentation of hypomethylated regions into CpG-rich UMRs and CpG-poor LMRs was performed using MethylSeekR (segmentUMRsLMRs(m = meth, meth.cutoff = 0.5, nCpG.cutoff = 5, PMDs = NA, num.cores = num.cores, myGenomeSeq = build, seqLengths = seqlengths(build), nCpG.smoothing = 3, minCover = 5).

Cooinda UMR coordinates were converted to the Desert dingo genome assembly using LiftOver following the genomewiki.ucsc.edu pipeline (http://genomewiki.ucsc.edu/index.php?title = Minimal_Steps_For_LiftOver). Briefly, the query (Desert dingo) genome build was split into individual scaffolds using *faSplit* (i). The we performed pairwise sequence alignment of query sequences from (i) against the Cooinda genome build using BLAT. Then, coordinates of .psl files were changed to the parent coordinate system using *liftUp*, and alignments were chained together using axtChain. Chain files were combined and sorted using *chainMergeSort*; alignment nets were made using *chainNet*. Finally, a liftOver chain file was created using *netChainSubset*. Cooinda UMRs in .bed format were lifted over to the Desert dingo genome assembly using a created liftOver chain file. Average methylation was calculated for Cooinda UMRs and compared to that of corresponding lifted-over regions in the Desert dingo genome. Cooinda UMRs with a >50% methylation increase in the Desert dingo genome were considered hypermethylated in the Desert dingo.

### Morphology

#### Skull morphometrics

To examine cranial morphology, we obtained a 3-dimensional model of Cooinda's cranium using an (Artis Pheno, Siemens Healthcare, VIC, Australia) computed tomography (CT) scanner. The skull was damaged slightly when the brain was extracted, so the damaged region (dorsal part of the calvarium) was reconstructed using Blender to reassemble the separated fragment following guidelines for digital specimen reconstruction outlined by Lautenschlager [128] (Supplementary Fig. S10A). Geometric morphometric landmarks (*n* = 45) were collected on the 3-dimensional cranial model using Stratovan Checkpoint (version 2018.08.07; Stratovan Corporation, Davis, CA, USA) and analyzed with MorphoJ [129], following the landmarking protocol used for dingo crania by Koungoulos [65]. This approach uses 45 landmarks along the left side of the cranium, covering all major anatomic features and regions, excepting a few fragile processes that are frequently lost in prepared specimens (Supplementary Fig. S11; Supplementary Table S4). The cranial landmarks collected on the Cooinda cranium were incorporated into an existing dataset comprising 91 Alpine dingoes and 101 Desert dingoes [65] and subject to Procrustes superimposition to remove all nonshape differences due to translation, rotation and scaling [130]. The resultant Procrustes shape variables were ordinated using principal components analysis (PCA) to assess the cranial morphology of Cooinda in relation to other dingoes. To assess the impact of allometry on cranial shape variation in the sample, a regression of Procrustes shape variables against log centroid size was performed using MorphoJ [129]. Residuals were extracted from this regression and ordinated using PCA (see Supplementary Material).

#### Brain imaging

Cooinda's brain and that of a domestic dog (Kelpie) of the same body size were extracted. Brains of these animals, which died within 2 weeks of each other, were fixed in Sigma-Aldrich (St. Louis, MO, USA) 10% Neutral Buffered Formalin after extraction and were washed with Gd DTPA (gadolinium-diethylenetriamine pentaacetic acid) solution prior to imaging. Brains were scanned using high-resolution magnetic resonance imaging (MRI). A (Bruker, Billerica, MA, U.S.) Biospec 94/20 9.4T high-field preclinical MRI system was used to acquire MRI data of a fixed dingo and domestic dog brain. The system was equipped with microimaging gradients with a maximum gradient strength of 660 mT/m and a 72-mm Quadrature volume coil. Images were acquired in transverse and coronal orientation using optimized 2- and 3-dimensional fast spin echo and gradient echo methods. Image resolution was 200 × 200 × 500 and 300 × 300 microns isotropic for type 3- and 2-dimensional pulse sequences, respectively. To quantify brain size, we used the open-source software 3D Slicer “Segment Statistics” module [66]. The software considers the pixel spacing and slice thickness set to calculate the volume accurately. The threshold was empirically set to the grayscale intensity of 1495, where everything below that is background, and ventricles and everything above that is the brain.

## Supplementary Material

giad018_GIGA-D-22-00267_Original_Submission

giad018_GIGA-D-22-00267_Revision_1

giad018_GIGA-D-22-00267_Revision_2

giad018_Response_to_Reviewer_Comments_Original_Submission

giad018_Response_to_Reviewer_Comments_Revision_1

giad018_Reviewer_1_Report_Original_Submission Andreas Chavez -- 12/2/2022 Reviewed

giad018_Reviewer_1_Report_Revision_1 Andreas Chavez -- 1/30/2023 Reviewed

giad018_Reviewer_2_Report_Original_Submission Jack Tseng -- 12/9/2022 Reviewed

giad018_Reviewer_3_Report_Original_Submission Sven Winter -- 12/9/2022 Reviewed

giad018_Reviewer_3_Report_Revision_1 Sven Winter -- 1/19/2023 Reviewed

giad018_Reviewer_4_Report_Original_Submission Madeleine Geiger -- 12/14/2022 Reviewed

giad018_Supplemental_Figures

## Data Availability

The chromosomal assembly is available at NCBI GenBank under the accession number GCA_012295265.2 (Bioproject: PRJNA613141). The mtDNA has been submitted to NCBI GenBank (accession: OP476512). The methylation data are available at Gene Expression Omnibus (GEO), accession Nr GSE212509. The 3-dimensional cranial landmark data are available on Figshare [131]. The raw Dicom data for the MRI of the Alpine dingo and domestic dog brain are also available on Figshare [132]. Assembly files, annotations, BUSCO results, and other supporting data are also available via the *GigaScience* database GigaDB [133].

## Additional Files

**Supplementary Fig. S1**. Schematic overview of project workflow. Alpine Dingo Cooinda DNA was derived from blood of a single female from the Dingo Sanctuary Bargo. Sequences were generated on the Pacific Biosciences Sequel instrument (V2 chemistry) and Oxford Nanopore PromethION instrument (guppy bascaller Version 3.0.6+9999d81) to ∼30× genome coverage, each based on a genome size estimate of 2.4 Gb (this estimate is used for all coverage estimates). All long-read sequences were assembled with the Canu v1.8 algorithm and then error corrected twice using the Arrow genomic consensus polishing module. The assembly was scaffolded with Chromium 10X linked reads (∼41× coverage excluding the barcode) with Long Ranger v2.1.6 using DNA from the same animal. Polishing of the assembly for residual indels was done by aligning the Illumina data with Minimap2 and the Racon algorithm. Single-molecule Bionano data (∼57× effective coverage) were then used to super-scaffold the sequence assembly using DNA extracted from the same canid. For this, single-molecule optical maps were first *de novo* assembled into consensus maps, which were then aligned to the sequence assembly *in silico* digested with the same labeling enzyme for hybrid scaffolding, using Bionano Solve (v3.2.2_08022018) with RefAligner (7782.7865rel). This assembly was further scaffolded to chromosome length by DNA Zoo (www.dnazoo.org/methods). Briefly, an *in situ* Hi-C library was prepared from the same individual and sequenced to 29× coverage. The Hi-C data were processed using Juicer [111] and used as input into the 3-dimensional DNA pipeline [112] to produce a candidate chromosome-length genome assembly. We performed additional finishing on the scaffolds using Juicebox Assembly Tools [113]. The assembly was then long-read gap filled with the PBJelly algorithm and the additional data error corrected using Arrow [32]. The Chromium data were mapped onto the assembly with the Long Ranger v2.1.6 program, and the final assembly was then polished using the Pilon algorithm. Of the 2.4-Gb assembled genome, the total assembly N50 contig and scaffold lengths are 23.1 Mb and 64.8 Mb, respectively. The assembled contigs were then aligned to CanFam3.1 for chromosome assignments. Regulatory landscape was characterized by whole-genome bisulfite sequencing.

**Supplementary Fig. S2**. Alpine dingo assembly after Hi-C correction. Contact matrices (visualized in Juicebox.js) after the chromosome-length Hi-C upgrade. The chromosome-length contact map can be viewed at multiple resolutions using Juicebox.js [34] following the link https://tinyurl.com/ycbkezf4.

**Supplementary Fig. S3**. Assembly statistics. (A) BUSCO ratings for Cooinda assembly, compared to CanFam4. Purple, original assembly; black, scaffolding/polishing steps; blue, final assembly; red, CanFam4. Dashed red lines mark CanFam4 statistics. (B) 10X read *k*-mer frequency distributions for *k*-mers with different assembly copy numbers derived from A Read 1 (16-bp barcodes trimmed) and B Read 2 (barcodes not trimmed).

**Supplementary Fig. S4**. Genome shows a deficiency of variation on the X chromosome. SNV and SV comparisons show a relative deficiency of variation on the X chromosome. Line represents a regression through the nontransformed data, and each point represents 1 chromosome with the length of the Alpine dingo and SNVs or SV relative to the Desert dingo genome or CanFam4. Y = 3.8e-4x + 21,305, 1.1e-4 + 31,753, 7.2e-5 + 406.7, 2.5e-5 + 363.1 with an *r*^2^ of 0.37, 0.74, 0.33, and 0.77 for SNV Desert dingo, SNV CanFam, SV Desert dingo, and SV CanFam, respectively. If the SNV and SV Desert dingo X chromosome data are excluded, the *r*^2^ of these regressions increases to 0.67 and 0.54, respectively.

**Supplementary Fig. S5**. Synteny analyses. Synteny plot of Alpine dingo Cooinda (AD) in blue against Desert dingo Sandy in orange (DD). (A) Shows the 3.45-Mb rearrangement on chromosome 16. (B) Shows the complex rearrangement between 55 and 57 Mb downstream on chromosome 16. (C) Smaller inversion on chromosome 11. (D) Small inversion on chromosome 25. (E) Multiple possible small inversions on the X chromosome. Other smaller rearrangements are possible. (F) Possible duplication-like events.

**Supplementary Fig. S6**. Gene order plot comparing chromosome 26. Gene order plot comparing chromosome 26 for Cooinda the Alpine dingo (x-axis) and Desert dingo Sandy (y-axis) using GeMoMa (left) and MUMmer (right). (A) In the GeMoMa plot (left), the green and the blue dashed lines indicate the 2 structural events on chromosome 26 of Cooinda. (B) MUMmer plot shows the same region.

**Supplementary Fig. S7**. Possible folding of 10-bp repeats in D-loop region: (A) 1 repeat, ∆G = −4.68; (B) 7 repeats, ∆G = −29.07; (C) 13 repeats, ∆G = −48.21; and (D) 28 repeats, ∆G = −97.71.

**Supplementary Fig. S8**. DNA methylation profiling of Alpine dingo Cooinda's whole blood. (A) Percentage of CpG sites with different levels of methylation. High, 80–100%; medium, 20–80%; low, >0–20%; no, 0%. (B) Average DNA methylation profiles of hypomethylated regions into CpG-rich UMRs and CpG-poor LMRs. (C) Integrative Genomics Viewer (IGV) browser track depicting DNA methylation profile and putative regulatory elements (UMRs and LMRs). (D) Heatmap depicting average DNA methylation at hypomethylated UMRs in the Alpine dingo genome, which are more than 50% methylated in the Desert dingo genome. (E) IGV browser track depicting hypomethylated UMRs within GCGR and HDAC4 genes in the Alpine dingo genome, which are hypermethylated in the Desert dingo genome.

**Supplementary Fig. S9**. Scatterplot of PC1 and PC2 values for sexed dingo specimens. The distribution of greater PC2 values slightly favors males in all populations except for the Central desert, which is a very gracile population with relatively minimal differences between the cranial morphology of different sexes. In general, however, the difference in PC2 between males and females in any population is very marginal, and neither greater nor lesser values are particularly strongly associated with either sex.

**Supplementary Fig. S10**. Cooinda's cranial morphology. (A) Cranium before (upper) and after (lower) cranial reconstruction (lower). This was required because the brain was removed immediately after death, which caused some damage to the brain case. (B) Cranium size. Cooinda's cranium is larger than the median size reported for Desert dingoes, in line with Alpine dingoes in general, although this difference is not major, and there is heavy overlap between the 2 regions. Her centroid size (392.80 mm) is slightly below the pooled Alpine mean (396.49 mm) and median (396.23 mm) but well below the mean (403.26 mm) and median (403.64 mm) for Alpine males specifically, which make up a majority of the sample (male, *n* = 50; female, *n* = 33; sex unknown, *n* = 9). Alpine dingoes, as with all regional dingo populations, exhibit significant sexual dimorphism in centroid size, with males being on average 4.20% larger [65]. (C) Principal component ordination of allometric residuals. The residuals of a regression of shape against log centroid size were plotted to further explore the role of size (allometry) in overall form. This revealed that the separation of Alpine and Desert populations, and Cooinda's position within the former, remains essentially identical to their original distributions (Fig. 5A) when the size-related allometric component of form is removed from consideration.

**Supplementary Fig. S11**. (A) Landmarks used in this study. (B) Diagram of canid skull with basic anatomic features and regions referred to in text. (C) Lollipop figures illustrating change in landmark positions along PC2 in lateral (upper) and dorsal (lower) views. The lollipop “head” represents the mean position, and the end of the “stick” represents its position with the highest PC1 score.

**Supplementary Table S1**. Alpine dingo SNV and SV summary by chromosome.

**Supplementary Table 2**. Distance matrix table showing SNVs above diagonal and indels below. All possible pairwise alignments were generated using MUMmer4 [44] (v4.0.0 beta 2) and SNVs/indels numbers calculated using MUMmer4 “show-snp” script.

**Supplementary Table S3**. Mean and median PC2 scores for different sexes from dingo populations.

**Supplementary Table S4**. List and description of cranial landmarks used in this study. After Koungoulos [65].

## Abbreviations

BLAST: Basic Local Alignment Search Tool; BMG: Bionano Genomics; bp: base pairs; BUSCO: Benchmarking Universal Single-Copy Orthologs; CNV: copy number variant; Gb: gigabase; gDNA: genomic DNA; GSD: German Shepherd dog; HME: high molecular weight; HMM: hidden Markov model; LMR: low-methylated region; Mb: megabase; MRI: magnetic resonance imaging; mtDNA: mitochondrial DNA: ONT: Oxford Nanopore Technologies; ORF: open reading frame; PacBio: Pacific Biosciences; PCA: principal components analysis; SNP: single-nucleotide polymorphism; SNV: single-nucleotide variant; SV: structural variant; UMR: unmethylated region.
