# Supplementary material for: The Australasian dingo archetype: de novo chromosome-length genome assembly, DNA methylome, and cranial morphology
Source: Gigascience. 2023 Mar 28;12:giad018. doi: 10.1093/gigascience/giad018 (PMC10353722; doi:10.1093/gigascience/giad018)
Supplement: giad018_GIGA-D-22-00267_Revision_1 [file giad018_giga-d-22-00267_revision_1.pdf]

GigaScience

The Australasian dingo archetype: De novo chromosome-length genome assembly, DNA methylome, and cranial morphology

--Manuscript Draft--

**Manuscript Number:**	GIGA-D-22-00267R1
**Full Title:**	The Australasian dingo archetype: De novo chromosome-length genome assembly, DNA methylome, and cranial morphology
**Article Type:**	Research
**Funding Information:**	Australian Research Council (DP150102038) — Professor J. William O. Ballard<br>National Health and Medical Research Council (APP5121190) — Dr Matt A Field<br>National Health and Medical Research Council Fellowship (APP5121190) — Dr Matt A Field<br>Australian Research Council (FT200100822) — Dr Laura A.B. Wilson

Abstract:

Background

One difficulty in testing the hypothesis that the Australasian dingo is a functional intermediate between wild wolves and domesticated breed dogs is that there is no reference specimen. Here we link a high-quality de novo long read chromosomal assembly with epigenetic footprints and morphology to describe the Alpine dingo female named Cooinda. It was critical to establish an Alpine dingo reference because this ecotype occurs throughout coastal eastern Australia where the first drawings and descriptions were completed.

Findings

We generated a high-quality chromosome-level reference genome assembly (Canfam_ADS) using a combination of Pacific Bioscience, Oxford Nanopore, 10X Genomics, Bionano, and Hi-C technologies. Compared to the previously published Desert dingo assembly, there are large structural rearrangements on Chromosomes 11, 16, 25 and 26. Phylogenetic analyses of chromosomal data from Cooinda the Alpine dingo and nine previously published de novo canine assemblies show dingoes are monophyletic and basal to domestic dogs. Network analyses show that the mtDNA genome clusters within the southeastern lineage, as expected for an Alpine dingo. Comparison of regulatory regions identified two differentially methylated regions within glucagon receptor GCGR and histone deacetylase HDAC4 genes that are unmethylated in the Alpine dingo genome but hypermethylated in the Desert dingo. Morphological data, comprising geometric morphometric assessment of cranial morphology place dingo Cooinda within population-level variation for Alpine dingoes. Magnetic resonance imaging of brain tissue show she had a larger cranial capacity than a similar-sized domestic dog.

Conclusions

These combined data support the hypothesis that the dingo Cooinda fits the spectrum of genetic and morphological characteristics typical of the Alpine ecotype. We propose that she be considered the archetype specimen for future research investigating the evolutionary history, morphology, physiology, and ecology of dingoes. The female has been taxidermically prepared and is now at the Australian Museum, Sydney.

Corresponding Author: J. William O. Ballard, Ph.D.
University of Melbourne Department of Zoology: The University of Melbourne School of BioSciences
Melbourne, Victoria AUSTRALIA

Corresponding Author Secondary

Powered by Editorial Manager® and ProduXion Manager® from Aries Systems Corporation

**Information:**	
**Corresponding Author's Institution:**	University of Melbourne Department of Zoology: The University of Melbourne School of BioSciences
**Corresponding Author's Secondary Institution:**	
**First Author:**	J. William O. Ballard, Ph.D.
**First Author Secondary Information:**	
**Order of Authors:**	J. William O. Ballard, Ph.D.
	Matt A Field
	Richard J. Edwards
	Laura A.B. Wilson
	Loukas G. Koungoulos
	Benjamin D Rosen
	Barry Chernoff
	Olga Dudchenko
	Arina Omer
	Jens Keilwagen
	Ksenia Skvortsova
	Ozren Ozren Bogdanovic
	Eva Chan
	Rob Zammit
	Vanessa Hayes
	Erez Lieberman Aiden, PhD
**Order of Authors Secondary Information:**	
**Response to Reviewers:**	Reviewer reports:<br>Reviewer #1: The article "The Australasian dingo archetype: De novo chromosome-length genome assembly, DNA methylome, and cranial morphology" is well written and interesting study examining the evolutionary relationships between the focal species, the dingo, and related canids (both domestic and wild). This study uses an impressive amount of state-of- the-art genomic data and resources to produce a (chromosome-length) de novo assembly of the dingo genome. The approach for assembling the genome are all adequate. The comparisons of chromosomal structural variation and methylation patterns with another dingo ecotype and other canids show interesting patterns of divergence that are potentially important regions of adaptive differences. I have two major concerns and some minor concerns for this paper.<br><br>Major concerns: I believe this paper would be stronger if it contained analytical methods that addressed admixture between dingos and domestic dogs more explicitly. The authors state that admixture between dingos and domestic dogs (Line 453) is one of a few hypotheses that may explain phenotypic differences between the two dingo ecotypes. To evaluate this hypothesis with the genomic data, they rely primarily on phylogenetic analyses to explore the evolutionary relationships between the dingo, wolf, and domestic dog lineages. They show that the dingo lineages are outside of the domestic dog clade and that wolves are outside of the dog/dingo clade. Although it is probably true that dingos are a unique evolutionary lineage, phylogenetic analyses are not the strongest tool for assessing admixture and the contribution of genomic variation from different ancestral source populations. I would recommend using methods that would test admixture hypothesis more explicitly. D-statistic tests (ABBA BABA test) and related tests would seem appropriate for this kind of data and sampling scheme.<br>REPLY: Here we suggest that inclusion of D-statistic tests is beyond the scope of the

Powered by Editorial Manager® and ProduXion Manager® from Aries Systems Corporation

present study for three reasons.
1. We do acknowledge there are a multitude of downstream analyses that can be completed using the data presented in this study. D-statistic tests are certainly one. Assessment of the divergence time of the lineages is a second. Both these types of studies are best done with multiple individuals within each population. We plan to collect whole genome data from 12 dingoes of each ecotype and complete these analyses in a subsequent manuscript.
2. It is not the goal of the study to differentiate dingoes from dogs.
3. From a stylistic perspective, we are wary to include these population genetic analyses as it has the potential to shift the balance of the study, which has been carefully considered throughout.

I also have concerns about the interpretations of brain size differences between dogs, dingos, and wolves. Although I am intrigued by the idea that domestication may have driven reductions in brain size and shape variation, I find it hard to not consider natural selection pressures in the case of dingos and wolves. The best scenario for testing the domestication- driven hypothesis would be if dogs, dingos, and wolves evolved in a common environment and domestication practices were the most notable differences between them. However, given that wolves and dingos in Australia evolved with different prey and habitats on different continents, it seems hard to me to not consider environmental adaptations as another important factor in the evolution of brain-size and shape variation.
REPLY: We agree that there may be additional, but difficult to test, factors that could impact brain-size evolution. Although there's a robust amount of data across many domesticates (reviewed in Balcarcel et al. 2022) that document brain-size reductions associated with domestication, you're right that environment is an important consideration for each domestication case. We've now noted this in the Discussion.

Minor concerns:
Introduction: It took me awhile reading deeper into the manuscript to understand what was meant by the name Cooinda. For awhile, I thought it was the name for a dingo subspecies or ecotype. I would suggest including a brief section in the introduction stating that the genomic and morphological data in this study is based off of a single individual named Cooinda and that there are questions about it's ancestry and placement as one of the dingo ecotypes.
REPLY: Added comments to the Figure 1 title and legend.

Line 172: ")," should be replaced with ")."
REPLY: Sentence replaced.

Line 371: I don't think it is necessary to say "The passing of Cooinda the dingo"
REPLY: Paragraph restructured.

Line 463: more is needed to finish the point of "will illuminate"
REPLY: now says "illuminate genetic history."

Line 494-497: The role of venomous animals as barriers to gene flow is conceptually not clear and is not supported by the citations from what I can readily tell.
REPLY: This is now reworded to improve clarity.

Line 540: dewclaws?
REPLY: Corrected

Line 541: "Regrettably" isn't necessary to include
REPLY: Removed

Reviewer #2: My evaluation of the manuscript was restricted to the geometric morphometrics (GM) section. The authors seem to have followed a standard GM procedure in their analysis of cranial shape differences among dingo skull samples. My only suggestion is that additional detail be provided in the landmark data collection for the GM analyses:
REPLY: Added as Supplementary Table 4.

Reference 58 was cited as the source of the landmarks used in this study, but no other

Powered by Editorial Manager® and ProduXion Manager® from Aries Systems Corporation

details are provided. A list of landmarks that forms the basis of the geometric morphometric analyses should be presented in order for the reader to fully interpret the PCA plots.
REPLY: This has been now addressed in the supplementary info (Supplementary Figure 11a, and Supplementary Table 4.)

Reviewer #3: The manuscript " The Australasian dingo archetype: De novo chromosome- length genome assembly, DNA methylome, and cranial morphology" does describe a de novo genome assembly of the Alpine dingo based on PacBio, ONT, 10X Genomics Chromium, BioNano, and Hi-C. Furthermore, it describes cranial morphometrics and methylation patterns to describe an Alpine dingo "archetype". The methods used seemed overall sound, yet, the writing was often confusing and unclear, so it was difficult to understand what was done and why. The writing, in general, is my biggest criticism of the manuscript, so much so that I was wondering if the authors, by accident, uploaded an earlier version of it. Throughout the manuscript, multiple writing styles and skill levels are evident, and I am sorry to say that it seemed as if the manuscript was copied together from different sources written by the different co-authors rather than a coherent manuscript. Some of the Figure Captions have superscript numbers to highlight individuals and at the same time proper labels that make them obsolete. I first thought they were remnants of footnotes in a previous version. Unfortunately, the methods section, for me one of the most important sections, needs serious improvements. I am not a big fan of having the methods at the end of the manuscript (I know that is how GIGAScience likes it), especially when some methodology is mentioned in the results in a way that you must look up the details in the methods section to understand it. Unfortunately, that is the case with this manuscript.
As there are so many paragraphs in this manuscript that need some improvements, I can only focus on some of them in this review but encourage the authors to have a careful look at the whole manuscript before resubmission, as in the current state, I would not recommend it for publication.
REPLY: This is a large and interdisciplinary m/s. Certainly different sections were written by different authors. The whole manuscript was then pulled together by JWOB before recirculating. We do appreciate the comments made and have made an effort to improve readability. We now feel the text of the manuscript is improved.

Detailed Comments:
Abstract:
The abstract is overall too long and needs to be much more concise, e.g., the discussion on taxonomic designation (L75-78) should be part of the discussion section but not the background paragraph of the abstract.
L91 "this female" which female?
REPLY: 75-79 removed.
REPLY: Now says “the dingo Cooinda”.

Introduction:
I am missing a short review of the taxonomy of the dingo, mostly with respect to the dog or wolf. I know you do not want to draw taxonomic conclusions from this study, but a short review of what others proposed would be helpful. Also, even though everybody knows what a dog is, scientific taxon names are a requirement in scientific writing and should be added at the first mention of any taxon (e.g., dog, gray wolf, dingo, etc.).
REPLY: Section now added to the Introduction. Introduction of this section necessitated restructuring the Introduction. We think this revised structure works well.
REPLY: Scientific names added.

L113: intermediate in what sense? Morphological, behavioral, ecologically?
REPLY: added “evolutionarily intermediate.”

L123: Please explain in more detail why a type specimen is needed, especially, as I would argue, that a population-level genomic, morphological and behavioral study would be better to answer if dingoes are feral dogs or an intermediate form instead of a single individual type specimen.
Added: “because we do not have a single reference point that links the scientific name to a specific specimen [30].”

Powered by Editorial Manager® and ProduXion Manager® from Aries Systems Corporation

	L139: reevaluation<br>REPLY: Corrected<br><br>L142: this can be more concise, e.g., "Zhang et al. (17) found evidence for a separation of Australian dingoes into a northwestern group and a southeastern group, clustering with New Guinea Singing dogs"<br>REPLY: Corrected as suggested<br><br>L150: remove "?"<br>REPLY: Corrected as suggested<br><br>L151-154: This belongs in the discussion.<br>REPLY: Corrected as suggested<br><br>L153: "... being characterized. However, we suggest ...."<br>REPLY: Removed as suggested above.<br><br>Results:<br>L161: Please consider changing it to chromosome-scale or chromosome-level genome assembly, which is much more common.<br>REPY: We used this terminology to distinguish it from the mitochondrial genome. However, have now changed the title following some debate.<br><br>L162-170: This whole paragraph is a short summary of the methods and does not include a single result. I know it sometimes is nice to recap the methods but in this case I do not see the need for it. Or at least it can be shortened even more to something like: "The final assembly after hybrid long-read assembly, polishing, and scaffolding has a total length of 2,398,209,015 bp ...."<br>REPLY: Here we disagree with the Reviewer. In the GigaScience format we suggest that an overall summary is very useful at the beginning. In the re-write of this paragraph we do include the Results of the final round of polishing.<br><br>L163: I'll highlight it again in the methods, but Supplementary Figure 1 shows 18 pacbio SMRT cells were used, but the methods say 15.<br>REPLY: 18 SMRT cells.<br><br>L164: please be more precise were they pacbio CCS or CLR reads?<br>REPLY: Clarified.<br><br>L167: Please provide Supplementary Figure 2 with a better contrast allowing us to see the high and low contact density on the centre of the scaffold squares.<br>REPLY: Contrast has now been increased by 25%. You can interactively explore the chromosome-length contact map at multiple resolutions using Juicebox.js (Robinson et al., 2018) following the link given in the legend to Supplementary Figure 2 (https://tinyurl.com/ycbkezf4).<br><br>L172: ungapped is not a term I would use; instead, I prefer to refer to the assembly as scaffolded or scaffold-level if it is in scaffolds with gaps and contig-level if the scaffolds are split up into contigs for statistics or analyses. In this case, I would only state the total scaffolded length and maybe the amount of N's or gaps. Also, the second sentence would be better combined with the first e.g., "The final assembly had a total length of 2,398,209,015 bp in 477 scaffolds and a scaffold and contig N50 of 64.8 Mb and 23.1 Mb, respectively." REPLY: Ungapped is an NCBI term and we follow their lead.<br>REPLY: Sentence corrected as suggested.<br><br>L174: What does full-length mean?<br>REPLY: Changed to Chromosome-level scaffolds<br><br>L175: please reference the dog genome properly with the accession number and reference if available.<br>REPLY: Reference added.<br><br>L176: Please rewrite. There is something not quite right with the bracket and the

Powered by Editorial Manager® and ProduXion Manager® from Aries Systems Corporation

following remaining sentence.
REPLY: Yes, missing a bracket. In response to Reviewer 3 the section has been split into two.
In the Results, simplified to:
Evaluation by Benchmarking Universal Single-Copy Orthologs (BUSCO v5.2.2 [37]) against Carnivora_odb10 data set (n=14,502) indicated that 95.1 % of the conserved single-copy genes were complete (Table 1, Supplementary Fig. 3A). Only 3 of 13,791 complete (single-copy or duplicated) BUSCO genes were not on the 39 nuclear chromosome scaffolds.
In Materials and Methods:
"Assembly completeness was evaluated using BUSCO v5.2.2 [37] short mode against the Carnivora_ob10 data set (n=14,502) implementing BLAST+ v2.11.0 [116], HMMer v3.3 [117], and Metaeuk v20200908 [118]."

L178: Carnivora_odb10
REPLY: Corrected

L182: Please check the manuscript and the supplementary data for consistent spelling of Cooinda (or Cooindah).
REPLY: Cooinda

L184: what does "were full-length by BUSCOMP" mean? Please give more details here on which basis this is determined and what it means that the two other genomes hat a few more. Also, I am not sure if you have to repeat the list with canine assemblies if you have them properly listed in the methods. Again, that's why I prefer to have the methods before the results.
REPLY: Reworded Results: Of the 13,722 genes, 13,711 were found in the Alpine dingo Cooinda assembly using BUSCOMP v1.0.1. Only Sandy the Desert Dingo v2.2 (13,715 genes) and China the Basenji v1.2 (13,712 genes) had more.
REPLY: Reworded Materials and Methods "Complete" BUSCO genes with available sequences were compiled across Alpine dingo Cooinda and nine canid genomes (Desert dingo [6], two Basenji's (China and Wags) [27], two German shepherd dogs (Nala and Mischa) [28, 36], Great Dane [38], Labrador [39], Dog10K Boxer [40], and Greenland Wolf [41]) using BUSCOMP v1.0.1.
REPLY: We include a list of the assemblies in the Results for ease of reading in the GigaScience format.

Table1: Again "ungapped" sounds not right. Please consider changing it to be clearer. As a general side note, when you want to compare two assemblies of different assembly length, it would be better to compare NG50 instead of N50. I doubt that in this case, with only a 40- 50Mb difference, it would change the results much but consider adding NG50 values. Number of gaps is also not very clear, as the gaps can be of different sizes and can be of a determined length or a standard number of N's as a placeholder for a gap of unknown length.
REPLY: We have followed the standard formatting of NCBI for eukaryotic genome assembles. https://www.ncbi.nlm.nih.gov/assembly/GCA_012295265.2.

L198: "to align Alpine dingo long reads to the Desert dingo assembly" seems not to fit here. Please check the sentence structure and rephrase.
REPLY: Reworded to improve clarity.

L199: "These plots show low variation on the X chromosome" More context is needed. low compared to? Why are the results only so briefly mentioned after multiple lines of "methods". This is an issue I see throughout the results. There are barely any results and mostly method summaries.
REPLY: Added "In comparison to the autosomes,".
REPLY: We have aimed to keep the manuscript as succinct as possible.
REPLY: This is most generally a function of having the Results before the Materials and Methods. We have tried to balance the manuscript more in the direction that the reviewer considers suitable while maintaining the GigaScience flow.

Figure 2: Explain what the plot shows. I am, in general, not a big fan of these multi-layer circus plots as each individual plot is way too small to show much. However in this case the lower amount of SVs on the X chr is visible enough, but the caption needs

more details.
REPLY: We have added “The plot shows the low variation on the X chromosome compared to the autosomes.”

L211: Why list a reference for something that is a results of this study?
REPLY: Reference deleted.

Supplementary Fig. 5: Each chromosome is too small and the resolution too low to see details of the SVs.
REPLY: This is a Supplementary Figure and aimed at giving the reader a general impression of the differences. We expand upon the major SV’s in the text.

L217: So why is that important to mention? If there is no further reason I would remove it, it does not add to the story.
REPLY: These lines expand upon the SV’s (that are difficult to see in Supplementary Fig. 5).

L226: "In addition, however, we also found ..." à change to "In addition, we found... " or "We also found ..."
REPLY: Changed to “We also found”.

L227: Consider joining the two sentences: "We also found two structural events on Chromosome 26 (SFig. 6) containing mostly short genes..."
REPLY: Changed as suggested.

L227: What does perfectly conserved mean?
REPLY: It appears the position of these genes has changed over time. Again, this is important detail and is necessary to mention.

L228-229: Why not show it?
REPLY: MUMMER PLOT added to Supplementary Fig. 6.

L230-232: This can be more concise and easier to read for example: "The Alpine and Desert dingo both have a single copy pancreatic amylase gene (AMY2B) on Chr 6. However, only the copy in the Desert dingo includes a 6.4kb long LINE." I am not sure why reference 10 is cited twice here in the results. Is this a result already known before? If so, this belongs in the discussion.
REPLY: Reworded.

L233: Again, the whole section is a short methodological summary, and there are absolutely no results.
REPLY: This section is included to guide the reader as the Materials and Methods are after the Discussion. The reviewer has stated s/he does not like the format.

Figure 3: The figure caption needs to be rephrased completely. Not sure how this ended up here, but "NOTE: A and C as well as B and D are similar plots. However, A and B use SNVs while C and D use indels." really does not belong in a proper caption, especially as each plot is listed before stating if it is based on SNVs or indels. Bootstrapping usually does not need to be explained in a figure caption. Instead, it would be more important to mention what type of phylogenetic tree it is and on how many SNVs it is based. There is also no scale on the trees, does that mean these are pure cladograms? For B and D, please explain what an ordination analysis is and change the axis labels to something meaningful. Labeling the x and y axis "Axis 1" and "Axis 2" is absolutely pointless. I am quite surprised that this passed the final ok from all co-authors. .
REPLY: Some legend moved to text. Bootstrapping explanation removed and now in Materials and Methods.
REPLY: Added to legend - Branch length proportional to the number of changes and bootstrapping percentage in circles.
REPLY: For B and D we follow the axis labelling convention used in Field et al., (2022). Axis 1 and Axis 2 are similar in concept to PC1 and PC2 which is the classical way to denote Principal Component axis labels (e.g., Supplementary Fig. 9).

L260: please use Desert dingo and not Sandy.

Powered by Editorial Manager® and ProduXion Manager® from Aries Systems Corporation

REPLY: Changed to Sandy Desert Dingo to parallel Cooinda Alpine dingo.

L263-265: Not sure why this is important here if it is not discussed later.
REPLY: We include this here as a Result that can be built upon.

Figure 4: Again, the figure caption needs a complete rewrite. For example, L281 "dingo Sandy is in this clade" is very unclear and confusing; "In this figure," à remove!; What are the superscript numbers for? Please remove them, they look like they belong to some footnotes from an earlier manuscript version, which are now missing. Instead, important info such as the type of network, the meaning of the small lines, and the scale are missing.
REPLY: Superscripts deleted. Type of network analyses now included, and the small cross hatched lines defined.

Methylome:
L295: I would remove "the" before MethylSeek and a period is missing before UMRs.
REPLY: Corrected as suggested.

L297-299: I think here it would be very nice to not just mention that there are other studies but give some examples and comparisons. "These analyses" could either refer to the MethylSeek analyses or the analyses of reference 55, please rephrase to be clearer. Also, it is unclear what previously reported numbers mean, again give more details ("... in line with previously reported numbers of promoters and enhancers in, e.g., humans (promoters xxxx, enhancers xxxx), mouse (xxxx), and rat (xxxx).")
REPLY: We thank the reviewer for this suggestion. To provide more clarity and give extra details, this sentence now reads as: “MethylSeekR analysis identified ~ 19,000 UMRs and ~44,000 LMRs in line with previously reported numbers of promoters and enhancers (e.g human: ~18,000-20,000 UMRs and 40,000-70,000 LMRs; mouse: ~17,000-19,000 UMRs and 55,000-90,000 LMRs)”

L301: what does "we lifted over the former UMRs to the latter genome" Please rephrase, it is very unclear to me what you mean.
REPLY: We have now re-phrased the sentence and added extra information to the methods section (page 37). It now reads as: “To establish whether proximal gene regulatory regions in the dingo Cooinda genome display different methylation states in the dingo Sandy genome, we converted Cooinda UMR coordinates from Cooinda to Sandy genome assembly using LiftOver (see Methods)”.
Methods (page 37): Cooinda UMR coordinates were converted from Cooinda dingo to Sandy dingo genome assembly using LiftOver following genomewiki.ucsc.edu pipeline (http://genomewiki.ucsc.edu/index.php?title=Minimal_Steps_For_LiftOver). Briefly, the query (Sandy) genome build was split into individual scaffolds using faSplit (i). The we performed pairwise sequence alignment of query sequences from (i) against the Cooinda genome build using BLAT, Then, coordinates of .psl files were changed to parent coordinate system using liftUp and alignments were chained together using axtChain. Chain files were combined and sorted using chainMergeSort; alignment nets were made using chainNet. Finally, liftOver chain file was created using netChainSubset. Cooinda UMRs in .bed format were lifted over to Sandy genome assembly using created liftOver chain file. Average methylation was calculated for Cooinda UMRs and compared to that of corresponding lifted- over regions in Sandy genome. Cooinda UMRs with >50% methylation increase in Sandy’s genome were considered as hypermethylated in Sandy’s genome.

L302: why was average DNA methylation calculated for UMRs. Should they not be unmethylated by definition?
REPLY: We apologise for the confusion. Indeed, UMRs are hypomethylated by definition, however while the majority of UMRs possess 0-10% mean methylation levels (Figure 1b, Burger et al Nucleic Acids Research 2013), there exists some level of fluctuation of UMR methylation levels (up to 50%, a user-defined cut-off, please see below a paragraph on MethylSeekR parameters). Therefore, in order to determine whether UMRs identified in Cooinda genome possess differential methylation in Sandy genome, we compared average methylation levels of Cooinda UMRs and their corresponding coordinates in Sandy genome (lifted-over regions). Lifted-over regions with >50% methylation gain were deemed hypermethylated in Sandy’s genome. We have now added more information to the Methods section (page 37) to clarify the

analysis.
MethylSeekR first identifies hypomethylated regions as stretches of CpG sites with methylation levels below a user-defined cut-off meth.cutoff (set to 50% as per original publication Burger et al Nucleic Acids Research 2013, please refer to Methods section MethylC-seq data analysis, page 37: meth.cutoff=0.5) containing a minimal number of CpGs nCpG.cutoff=5. Then, separation of UMRs and LMRs (low-methylated regions, which correspond to distal regulatory regions) is performed based on the number of CpG sites (UMRs are defined as hypomethylated regions with n330 CpG sites) rather than methylation levels.

L306: Why have a sentence about that a gene is highly conserved but not perfect and then give the percentage of identity instead of just stating that it is 99.8% identical? I have now mentioned quite a few examples where the manuscript could be much more concise. I cannot list them all but would encourage you to read through the manuscript again and make it more concise.
REPLY: We thank the reviewer for this suggestion. We have now made it more succinct to read as: “GCGR is on chromosome 9 and has a single transcript. This transcript is 99.8% identical at the amino acid level between the dingoes. HDAC4 occurs on chromosome 25 and has 12 transcripts with all 12 transcripts being 100% identical at the amino acid level.”

Morphology:
My knowledge of morphological analyses is limited, but, despite the unfamiliar terminology, this section of the manuscript is easy to read and focuses in a more concise way on the actual results. My only suggestion would be to label Supplementary figure 9a with the different morphological features mentioned or adding an additional schematic to the supplementary, so non-morphologists can easier follow.
REPLY: There is now Supplementary Figure 10b for this purpose. 9a was not really suited to pointing out the features in a visually clear manner.

L353-354: I would suggest adding the sizes after you mention the individual to avoid repeating dingo and dog brain. For example: "... the dingo brain (75.25cm3) was 20% larger than the dog brain (59.53 cm3) (Figure 5B)."
REPLY: Corrected as suggested.

Figure 5: Please add an explanation of what the polygons in 5A represent. Also, consider changing the labels in 5B. I assume LHS and RHS are short for the left-hand side and the right-hand side. This, for me, is usually used to describe positions in unlabeled figures. I would suggest changing it to Cooinda dingo (CD) and domestic dog (DD).
REPLY: Done. We have re-labelled Fig 5B as suggested using CD and DD.

Discussion
L375: It is not clear why Cooinda should be considered the archetype at the beginning of the discussion. I would place this in the conclusions and base it on the results and the discussion. REPLY: Corrected as suggested.

L394-395: Please rephrase and shorten, e.g.,: "There is a single copy of AMY2B in both dingo genomes; however, they differ by a 6.3 kb retrotransposon insertion present in the Desert dingo."
REPLY: Corrected as suggested.

L394-405: I would like to see a more in-depth discussion on the differences between wolf, dingo, and dog. If there is no LINE in the wolf but both in the dingo and the dogs, when did the transposition happen? Could be two independent events in the dog and dingo lineages or one in the ancestral lineage. Are the LINEs in dog and dingo at the same position in the gene region? Could it be the same insertion that was reduced in length in the dog lineage, and what does that mean for the evolution of dogs and dingoes?
REPLY: Added, As the retrotransposon is absent in the Greenland wolf and Alpine dingo (and they are in different positions) it would seem likely that the retrotransposon has inserted into the Desert dingo and domestic dog lineages independently.

L431/432: please use Alpine and Desert dingo instead of the individuals' names.

Powered by Editorial Manager® and ProduXion Manager® from Aries Systems Corporation

	REPLY: We have changed Sandy to Desert dingo, but retain Cooinda for consistency.<br><br>L471-473: Not sure if a single sample (Cooinda) is sufficient to come to this conclusion, also how does it compare to the wolf? She could just have been a dingo with an exceptionally large brain. I think a more in-depth discussion is needed.<br>REPLY: added – "An alternative hypothesis is that differences in brain size is due to environmental adaptation or perhaps Cooinda was an anomaly."<br><br>Methods:<br>Overall, the methods need to be more concise but at the same time clear and complete. L530-531: Why is solving the puzzle-box experiment important? Does that not suggest an exceptionally intelligent dingo if she was the only one, and could that not potentially explain the large brain size? How does brain size and intelligence or the potential to solve the puzzle- box correlate?<br>REPLY: Phrase concerning puzzle box deleted.<br><br>L532: her brothers<br>REPLY: Corrected as suggested.<br>L533: What is the importance of the ginger color? As it is stated here, it is a bit out of context. Why is it important?.<br>REPLY: Here we are giving a context to Cooinda, Not all dingoes are ginger so a future study looking at coat colour will be interested in this detail.<br><br>L535-542: Why is this detailed report on her appearance of importance? I am often missing logical connections in the manuscript.<br>REPLY: As we are attempting to suggest Cooinda become the archetype morphological descriptions are important.<br><br>L541: I would usually not expect to read such a statement with an emotional connotation in a scientific manuscript.<br>REPLY: Now says "Cooinda died in 2019 at 10 years of age."<br><br>L545ff: When were the samples taken? What type of samples were taken? How were they preserved? As it is stated that fresh blood was used, I assume Cooinda was still alive at that point. Are there any sampling and ethics permits to be mentioned?<br>REPLY: As stated, DNA was extracted from fresh blood. An ethics approval statement is included before competing interests.<br><br>L552-56. This whole section about the pulse-field electrophoresis can be much shorter without losing any information, e.g., "Molecular integrity was assessed by pulse-field gel- electrophoresis using the PippinPulse (Sage Science) with a 0.75% KBB gel, Invitrogen 1kb Extension DNA ladder (cat ....) and 150 ng of DNA on the 9hr 10-48kb (80V) program."<br>REPLY: Modified as suggested.<br><br>L556: What libraries? You have not explained how the libraries were prepared. CLR or CCS?<br>REPLY: Included "SMRTbell libraries with 20kb insert size were CLR sequenced on Sequel I machines with 2.0 chemistry."<br><br>L557: Which Sequel platform was used? Sequel I, II or, IIe?<br>REPLY: As above, SMRTbell libraries with 20kb insert size were CLR sequenced on Sequel I machines with 2.0 chemistry.<br><br>L558: remove the hours of movies, that does not matter unless you used a custom sequencing program.<br>REPLY: OK<br><br>L559: AS 15 SMRT cells were used, I assume the sequencing was performed on the Sequel I.<br>REPLY: OK<br><br>L561: I usually avoid starting a section or paragraph with "for". Please consider rephrasing as you start most paragraphs that way.

	REPLY: Generally, we agree. In this case, we have done this intentionally so that the method is right at the front of the sentence. However, it does look a little repetitive so we have modified slightly.<br><br>L564: 119 ng of library, especially for long DNA-molecules, seems very low for a decent ONT run. I have mostly used the MinION, and I would usually only load a library with so little DNA if I only needed a few reads. I am just curious how well that worked on the larger PromethION flow cell.<br>REPLY: Thank you. This protocol was optimised at the Garvan in Sydney.<br><br>L573: In some sections, this manuscript reads like an early draft that was accidentally submitted.<br>"User Guide, manual part number CG00043 Rev B." Please rephrase.<br>REPLY: (Document # CG00043 revision B).<br><br>L575: For me personally, it does not matter where Qubit measurements were taken, but if you include that info, please try not to repeat it as you did in L578.<br>REPLY: Multiple reference to the Kinghorn centre deleted.<br><br>L576-577: Please shorten the two sentences about sequencing to one, e.g., "Sequencing was performed in 150bp paired-end sequencing mode on a single lane on the Illumina HiSeq X Ten platform with a version 2 patterned flowcell."<br>REPLY: Corrected as suggested.<br><br>L581-582: Does reference 8 use the same protocol version? If so, I would remove the brackets. If not, Is there no version number of the protocol available?<br>REPLY: Corrected as suggested (section in brackets removed).<br><br>L594-601: Why is this a mixture of insufficiently described methods and results? Please give additional information about trimming and assembly using canu. Why mention the number of sequences, bubbles, and unassembled sequences in the methods?<br>REPLY: The canu parameters used for assembly have been added. Details about the canu assembly stages (correction/trimming/assembly) can be found in the canu manuscript referenced. Details about the number of sequences have been moved to results.<br><br>L597: How were the reads aligned to the assembly? I have not used Arrow but if the pipeline uses mapping tools, please mention them.<br>REPLY: Additional details about the Arrow pipeline have been added as suggested.<br><br>L598-601: These are results and should not be part of the methods.<br>REPLY: Moved as suggested.<br><br>L614: what does finishing mean? In the literature, it is more common to write "manually curation of" or scaffolds were "manually curated" or "manually edited".<br>REPLY: Corrected as suggested.<br><br>L615: Again, these are results. I would not place them in the methods.<br>REPLY: Moved as suggested.<br><br>L621: Was gap-closing performed only once? Were pacbio and ONT reads combined on one iteration of gap-closing? Maybe PBJelly suggests only using it once, but in my experience, gap-closing can be performed iteratively to further improve the contiguity.<br>REPLY: Only one round of gap filling is suggested by the PBJelly documentation. PBJelly was run with the combined alignments of ONT and pacbio read.<br><br>L622-623: Again, results in the methods.<br>REPLY: Moved as suggested.<br><br>L634: Why is it important when the chromosome mapping was completed? You did not specify when the sequencing was performed or when the samples were taken.<br>REPLY: We suggest that this is important as it underpins why we mapped to CanFam3.1 and not 4.1.

L635: Please add accession number and, if available the reference.
REPLY: Accession number = GCF_000002285.3.
Citation: Hoeppner MP, Lundquist A, Pirun M, Meadows JRS, Zamani N, Johnson J, et al. (2014) An Improved Canine Genome and a Comprehensive Catalogue of Coding Genes and Non-Coding Transcripts. PLoS ONE 9(3): e91172. https://doi.org/10.1371/journal.pone.0091172

L644: Circos is a tool for plotting data in a circular plot. How were the SNV, and indels identified?
REPLY: Added - SNV and indel numbers were calculated using MUMmer4 'show-snp' script following pairwise alignments [37] (v4.0.0 beta 2).

L647: X chromosome
REPLY: Corrected as suggested.

L652: I usually use GeMoMo for homology-based gene prediction. I would like to see a short description of the method rather than [43] just linking to a previous publication.
REPLY: currently: We also used [41] GeMoMa to further investigate whole chromosomal events. Here we mapped genes onto the Alpine Dingo assembly following previously described protocols [8] and then determined the gene order.
new: We also used [41] GeMoMa to further investigate whole chromosomal events. Here we mapped genes onto the Alpine Dingo assembly following previously described protocols [8]. Subsequently, we checked the syntheny of the genes in the reference genome and the target genome using the module GeMoMa module SynthenyChecker. This module uses the GeMoMa annotation with information for reference gene and alternative to determine the best homolog of each transcript. Comparing the order of genes in the reference and the target genome, it allows to determine breakpoints of chromosomal events.

L658: "processes that produce differences" is not very precise, please give some more info here. I would usually remove Indels from phylogenetic datasets due to the uncertainty of their mutational history. How were they coded and how were they analysed?
REPLY: We now simply say "SNV's and indels were analysed separately"
REPLY: Different people have different approaches, so we analyzed both SNP's and indels, but kept them separate.
REPLY: Methods of analyses are presented in the two following paragraphs.

L659: What is WA distance? Reference?
REPLY: Its simply called WA distance. Reference 43 added.

L660: The Glazko et al reference is quite out of context here. Better phylogenetic properties than what?
REPLY: than other coefficients.

Why use distance-based phylogeny? How many SNVs were used? How were they filtered
REPLY: We used distance-based methods so the data could be analysed within weeks. REPLY: SNV's presented in Supplementary Table 2. As described, they were calculated using MUMmer4 'show-snp' script following pairwise aligments.

L662: Maximum parsimony is not frequently used anymore for phylogenetic reconstruction. Why not use a Maximum-likelihood or Bayesian approach?
REPLY: We used both distance and parsimony based methods. No method is perfect.

L664: It is mentioned that the wolf should be the outgroup, but the dataset itself is not mentioned in the methods. List all samples that were included in the phylogeny. If the dingo is assumed to be an intermediate between wolf and dog why did you not use a different canine as outgroup to avoid bias?
REPLY: We used all de novo canine genomes currently available.

L665: include version and the URL of the tool if there is no paper to cite.
REPLY: (github.com/lmc2179/bayesian_bootstrap)

I cannot judge the methods for Methylation and Morphology, as this is not my expertise, but these method sections read very well and seem clear to me.
REPLY: Thank you.

Availability of supporting data:
There are quite some broken sentences and misplaced periods in this section. Please check the text again and make sure that the links to your datasets are functioning.
REPLY: Figshare morphology datasets are live, and the links have been tested – please see:
https://figshare.com/articles/dataset/Dicom_data_MRI_Alpine_dingo_and_domestic_dog_bra in/20514693
https://figshare.com/articles/dataset/Cooinda_Alpine_Dingo_3D_Cranial_Landmarks/205238 04

Overall, the presented data are interesting and a valuable resource, but the manuscript itself needs some major improvements to make the interesting results available to the reader in an easier-to-follow and more understandable form. It is obvious that multiple authors with different expertise worked on different sections of the manuscript. The challenge during the revision is to bring it together into a concise and easy-to-read manuscript with a consistent writing style. I hope that my comments, questions, and suggestions can help in that process. Please take my writing suggestions as such, feel free to adjust and change it in a different way as long as the result is more reader-friendly and more concise.
REPLY: Thank you. By-and-large we followed the Reviewers suggestions. We have also made several changes to improve readability. The comments of the four reviewers are acknowledged.

Reviewer #4: This well written study integrates different approaches and methodologies to tackle the still obscure nature and origin of the dingo and its sub-populations by thoroughly characterising and comparing an "archetype" dingo specimen.
I have read and commented on the abstract and the introduction, as well as the morphology related parts of the methods, the results and the discussion.
The methods of morphological comparison, as well as their description and the reporting of the results are sound. However, in some sections it is difficult to comprehend the results and their interpretations, as well as the significance and nature of the suggested "archetype" specimen Cooinda. I therefore made some suggestions for additions and edits to the text and the figures, which hopefully help to increase comprehensibility and consistence of the text (see my comments below).
I could not check and comment on the raw data because the links to the supplement given in the manuscript (figshare) do not work. Sorry if I'm stating the obvious here, but to be able to access the raw data is particularly important if the described dingo should act as a reference archetype.

L. 74: Add «of the dingo» after "ecotypes": "[...] compare the Alpine and Desert ecotypes of the dingo [...]". Otherwise it's not really clear what this is about.
REPLY: Corrected as suggested.

L. 91: It's unclear to me what you mean by "this female". I would suggest to exchange this expression with the previously used name of the animal.
REPLY: replaced with “the dingo Cooinda”.

L. 94 ff.: The conclusions do not really fit to the rest of the abstract, specifically the aims as stated in the beginning. What I read from the "Background" section is that this work is about defining a "dingo archetype" via different approaches (genetic and morphological). The conclusion, however, is centred around the individual Cooinda. I would suggest to open up this section, to also make conclusions concerning the previously stated aims of the paper.
REPLY: Corrected to follow Reviewer 3.

L. 105 ff. and L. 369 and L. 508: A very nice opening! However, I feel that there is a somewhat misleading interpretation of the domestication process as a discrete trichotomy: wild > tamed > domesticated, when in fact domestication is a continuum

Powered by Editorial Manager® and ProduXion Manager® from Aries Systems Corporation

	with various stages in between the two extremes of the "wild" and the "intensively bred". There are various forms - even today - of "half-domesticated" populations, such as e.g., many of the Asian domestic bovids, or the reindeer. Thus, I would strongly argue that the dingo - although special due to the almost complete lack of human influence on its evolution in the last millennia - is not the only link between the "wild" and the "domesticated". See e.g.: Vigne, Jean-Denis. "The origins of animal domestication and husbandry: a major change in the history of humanity and the biosphere." Comptes rendus biologies 334.3 (2011): 171-181<br>REPLY: Yes, agreed, we have added further acknowledgment of the alternative hypothesis that domestication represents an intensification of the relationship between humans and animal/plant and have cited Vigne (2011) now in the introduction and discussion in relation to this comment. Lines 113, 385, 535.<br><br>L. 117: How do you define "large carnivore"? And: Are dogs more numerous than cats? I don't know the tallies overall, but in many parts of the world domestic cats are more frequent than dogs.<br>REPLY: To avoid confusion we've deleted 'large carnivore'<br><br>L. 120 - 121: I think this sentence does not contribute to the manuscript and I would suggest to delete it. I also think that these are not the usual characteristics to discern the wolf from other canids.<br>REPLY: Corrected as suggested.<br><br>L123 - 125: I do not understand this distinction. In my opinion, the dingo could well be both, a tamed intermediate between wolf and domestic dog AND a feral canid. If I understand the current view of dingo evolution correctly, the dingo most probably constitutes an early domestic stage of the dog, which became feral.<br>REPLY: We attribute these terms following their use by Darwin. We now say "One alternate hypothesis is that the process of domestication is continual and does not proceed in a stepwise manner [4]."<br><br>L. 150: I do not understand the reference to Figure 1 at this point. If you want to keep the figure reference at this place, I would recommend to extend the legend in order to be more descriptive about the significance of this individual dingo. Also: Is the question mark on purpose?<br>REPLY: Legend to figure 1 now expanded.<br><br>Intro and Results in general: Cooinda is central for the research question and the paper. However, I do not really understand her position and significance right away from the text. Maybe this is just a matter of sequence of the paragraphs (some information is given at the beginning of the methods section at the end of the manuscript), but I think it would be crucial to introduce and explain Cooinda and her role (as kind of a reference "archetype") for the aims thoroughly already early on, preferably in the Introduction. This would e.g. also include: why of all the dingoes in Australia is Cooinda an appropriate choice to function as the "archetype". Further, it would be helpful to maybe have a figure showing the geographical distribution of the compared populations (alpine and desert, as well as Cooindas origin) to better understand the setting.<br>REPLY: We suggest the short expansion of the Legend covers the majority of these points. The selection of Cooinda is presented in the Materials and Methods.<br><br>L. 320 ff. and Figure 5: Would it be possible to add a visualisation of the shape changes described in the text into the figure? It is otherwise impossible to evaluate these shape changes.<br>REPLY: Added – in Supplementary Figure 10.<br><br>L. 328 - 345: It would be interesting to pursue the variation along PC2 further: Do you maybe have information from the raw-data if specimens of both the alpine and the desert group that were found to have particularly low or high values for PC2 are especially young and female, or old and male? In other words, do you find evidence in the dataset that there is an actual age and/or sex gradient along PC2? And what age was Cooinda when she died?<br>REPLY: Thanks, we've added an additional supplementary figure and table to show sex- related trends in PC space, and have tabulated PC2 scores by sex and

	population. Please see new supplementary figure 11. Data on sex were only available for most of the alpine sample, some of the central desert and a small number of western desert dingoes. Despite a lot of overlap, PC2 scores tends to be lower in females than males in the alpine population and in the western desert. Although central desert males seem to have slightly larger centroid size on average, they score a lower PC2 than females. This is a very gracile population where sexually dimorphic differences are at their most minimal as far as dingoes are considered (Koungoulos 2022:138). We've added additional text to line 350-354 directing readers to the supplementary supporting information. All sampled dingoes were adults ≥ 1 y.o., but no further specificity regarding age was available (pulp-cavity X-rays are generally required for this purpose. As such the role of age in PC2 if any cannot be tested with the present dataset, though it could be (for example) that the male samples include more younger individuals than the female samples. Cooinda as an older individual certainly scores low in PC2.<br>REPLY: Cooinda was 10 when she died (now added to text).<br><br>L. 347: As also pointed out below, it would be important to note somewhere if these two specimens died at about the same time and/or were similarly treated (because of brain shrinkage in specimens that were frozen or otherwise fixed for a long time).<br>REPLY: Added to the Materials and Methods that "These animals died within 2 weeks of each other."<br><br>L. 472: I would suggest to rewrite as: "Cooinda's brain was 20% larger than that of a similarity sized domestic dog [...]". Further, I do not agree with the rest of the statement in this sentence. One of the hallmark characteristics of domestication is brain size reduction, which might be the result of selection for tameness (which you also describe later on). However, selection for tameness (an evolutionary process within a population) is not the same as taming (on the level of the individual). I would therefore suggest to re-write this sentence.<br>REPLY: Now says: Cooinda's brain was 20% larger than the domestic dog, which is consistent with the hypothesis that she was tamed but not domesticated [3].<br><br>Further and in general concerning the brain size part of this study: It would greatly increase the significance of this part of the work if you would compare the dingo brain size not only to one domestic dog, but set it into a larger context. There are plenty of published references for wolf, domestic dog, and dingo brain size estimates and it would be enlightening to compare your findings with those. Of course, there are methodological issues, but maybe a meaningful comparison is possible for some of them. For this I could recommend this review article: Balcarcel, A. M., et al. "The mammalian brain under domestication: Discovering patterns after a century of old and new analyses." Journal of Experimental Zoology Part B: Molecular and Developmental Evolution (2021).<br>REPLY: thanks, we've added direct comparison of our brain size data to previous published data on wolf-domestic dog brain size comparisons and we have also cited Balcarcel et al. Please see lines 490-496.<br><br>L. 483: Many of the surviving populations of re-introduced (i.e., feral) domestics were part of a fauna that did not correspond to the one of their wild relatives, but was somehow characterised by reduced predation or competition. This was certainly the case for the dingo (few other large predators in Australia) and for some island populations. Maybe you should double-check if this is really the case for the provided examples, but maybe it would be better to write that brain size reduction persists in feral populations at least under certain circumstances.<br>REPLY: Corrected as suggested.<br><br>L. 527: Why is it important that the reference dingo is a fe...
**Additional Information:**	
**Question**	**Response**
Are you submitting this manuscript to a special series or article collection?	No
**Experimental design and statistics**	Yes

Full details of the experimental design and statistical methods used should be given in the Methods section, as detailed in our Minimum Standards Reporting Checklist. Information essential to interpreting the data presented should be made available in the figure legends.<br><br>Have you included all the information requested in your manuscript?	
**Resources**<br><br>A description of all resources used, including antibodies, cell lines, animals and software tools, with enough information to allow them to be uniquely identified, should be included in the Methods section. Authors are strongly encouraged to cite Research Resource Identifiers (RRIDs) for antibodies, model organisms and tools, where possible.<br><br>Have you included the information requested as detailed in our Minimum Standards Reporting Checklist?	Yes
**Availability of data and materials**<br><br>All datasets and code on which the conclusions of the paper rely must be either included in your submission or deposited in publicly available repositories (where available and ethically appropriate), referencing such data using a unique identifier in the references and in the "Availability of Data and Materials" section of your manuscript.<br><br>Have you have met the above requirement as detailed in our Minimum Standards Reporting Checklist?	Yes

Powered by Editorial Manager® and ProduXion Manager® from Aries Systems Corporation

Click here to access/download;Manuscript;Dingo_GigaScience (Revision 5).pdf

The Australasian dingo archetype: *De novo* chromosome-length genome assembly, DNA methylome, and cranial morphology

J. William O. Ballard,[1,2*] Matt A. Field,[3,4] Richard J. Edwards,[5] Laura A.B. Wilson,[6, 7] Loukas G. Koungoulos,[8] Benjamin D. Rosen,[9] Barry Chernoff,[10] Olga Dudchenko,[11, 12] Arina Omer,[12] Jens Keilwagen, [13] Ksenia Skvortsova, [14] Ozren Bogdanovic, [14] Eva Chan,[14,15] Rob Zammit,[16] Vanessa Hayes,[14,17] Erez Lieberman Aiden[11,12,18,19,20]

1 School of Biosciences, University of Melbourne, Royal Parade, Parkville, Victoria 3052, Australia. Bill.Ballard@unimelb.edu.au

2 Department of Environment and Genetics, SABE, La Trobe University, Melbourne Victoria 3086, Australia Bill.Ballard@unimelb.edu.au

3 Centre for Tropical Bioinformatics and Molecular Biology, College of Public Health, Medical and Veterinary Science, James Cook University, Cairns, Queensland, Australia. matt.field@jcu.edu.au

4 Immunogenomics Lab, Garvan Institute of Medical Research, Darlinghurst, NSW, Australia. matt.field@jcu.edu.au

5 School of Biotechnology and Biomolecular Sciences, University of New South Wales, Sydney NSW 2052, Australia. Richard.edwards@unsw.edu.au

6. School of Archaeology and Anthropology, The Australian National University, Acton, ACT 2600, Australia. Laura.Wilson@anu.edu.au

7. School of Biological, Earth and Environmental Sciences, University of New South Wales, Sydney, NSW 2052, Australia. Laura.Wilson@anu.edu.au

8. Department of Archaeology, School of Philosophical and Historical Inquiry, the University of Sydney, Sydney, Australia 2006lkou2342@uni.sydney.edu.au

9. Animal Genomics and Improvement Laboratory, Agricultural Research Service USDA, Beltsville, MD 20705. ben.rosen@usda.gov

10. College of the Environment, Departments of Biology, and Earth & Environmental Sciences, Wesleyan University, Middletown, CT 06459, USA. B.chernoff@wesleyan.edu.

11. The Center for Genome Architecture, Department of Molecular and Human Genetics, Baylor College of Medicine, Houston, TX, USA. Olga.Dudchenko@bcm.edu, erez@erez.com

1.2 Center for Theoretical and Biological Physics, Rice University, Houston, TX 77005, USA. Olga.Dudchenko@bcm.edu, arinaomer@gmail.com, erez@erez.com

13. Julius Kühn-Institut, Erwin-Baur-Str. 27 06484 Quedlinburg, Germany Jens.keilwagen@julius-kuehn.de

14. Garvan Institute of Medical Research, Darlinghurst, NSW, Australia. k. Skvortsova@garvan.org.au, o.bogdanovic@gmail.com, eva.chan@health.nsw.gov.au, vanessa.hayes@sydney.edu.au,

15. Statewide Genomics, New South Wales Health Pathology, 45 Watt St, Newcastle NSW 2300, Australia

16. Vineyard Veterinary Hospital, 703 Windsor Rd, Vineyard, NSW 2765, Australia. razammit@me.com

17. Charles Perkins Centre, Faculty of Medical Sciences, University of Sydney, Camperdown, NSW, Australia. vanessa.hayes@sydney.edu.au

18. UWA School of Agriculture and Environment, The University of Western Australia, Perth, WA 6009, Australia. erez@erez.com

19. Shanghai Institute for Advanced Immunochemical Studies, ShanghaiTech, Pudong 201210, China. erez@erez.com

20. Broad Institute of MIT and Harvard, Cambridge, MA 02142, USA. erez@erez.com

ORCID IDS:

J. William O. Ballard [0000-0002-2358-6003]; Matt A. Field [0000-0003-0788-6513]; Richard J. Edwards [0000-0002-3645-5539]; Laura A. B. Wilson [0000-0002-3779-8277]. Loukas Koungoulos [0000-0002-5148-0142]; Benjamin D. Rosen [0000-0001-9395-8346]; Barry Chernoff [0000-0001-8439-4542]; Olga Dudchenko [0000-0001-9163-9544]; Arina Omer [0000-0003-1336-2505], Jens Keilwagen [0000-0002-6792-7076]; Ksenia Skvortsova [0000-0003-1400-1998], Ozren Bogdanovic [0000-0001-5680-0056], Eva Chan [0000-0002-6104-3763]; Rob Zammit [0000-0002-7520-8338]; Vanessa Hayes [0000-0002-4524-7280]; Lieberman Aiden [0000-0003-0634-6486].

[§]**Correspondence address**. Bill.Ballard@unimelb.edu.au. School of Biosciences, University of Melbourne, Royal Parade, Parkville, Victoria 3052, Australia.

.

Abstract

Background

One difficulty in testing the hypothesis that the Australasian dingo is a functional intermediate between wild wolves and domesticated breed dogs is that there is no reference specimen. Here we link a high-quality *de novo* long read chromosomal assembly with epigenetic footprints and morphology to describe the Alpine dingo female named Cooinda. It was critical to establish an Alpine dingo reference because this ecotype occurs throughout coastal eastern Australia where the first drawings and descriptions were completed.

Findings

We generated a high-quality chromosome-level reference genome assembly (Canfam_ADS) using a combination of Pacific Bioscience, Oxford Nanopore, 10X Genomics, Bionano, and Hi-C technologies. Compared to the previously published Desert dingo assembly, there are large structural rearrangements on Chromosomes 11, 16, 25 and 26. Phylogenetic analyses of chromosomal data from Cooinda the Alpine dingo and nine previously published *de novo* canine assemblies show dingoes are monophyletic and basal to domestic dogs. Network analyses show that the mtDNA genome clusters within the southeastern lineage, as expected for an Alpine dingo. Comparison of regulatory regions identified two differentially methylated regions within glucagon receptor GCGR and histone deacetylase HDAC4 genes that are unmethylated in the Alpine dingo genome but hypermethylated in the Desert dingo. Morphological data, comprising geometric morphometric assessment of cranial morphology place dingo Cooinda within population-level variation for Alpine dingoes. Magnetic resonance imaging of brain tissue show she had a larger cranial capacity than a similar-sized domestic dog.

Conclusions

These combined data support the hypothesis that the dingo Cooinda fits the spectrum of genetic and morphological characteristics typical of the Alpine ecotype. We propose that she be considered the archetype specimen for future research investigating the evolutionary history, morphology, physiology, and ecology of dingoes. The female has been taxidermically prepared and is now at the Australian Museum, Sydney.

Key Words: type specimen**,** cranium, long-read sequencing, de novo genome assembly, biogeography

Introduction

The most influential book on evolution, Darwin's 1859 *On the origin of species* [1], starts with a chapter on domestication to reverse engineer natural selection. Some nine years later Darwin [2] expanded his initial thinking into the book *The variation of animals and plants under domestication*. He hypothesized that the process of domestication proceeded in a stepwise manner first by unconscious selection (wild →tamed) followed by what we now call artificial selection (tamed →domesticated), with the key distinction between these processes being the involvement of humans on mating and reproduction. A gap in our ability to test Darwin's hypothesis has been the identification of a model system with an extant plant or animal that is intermediate between the wild ancestor and the domesticate. Here we explore the overarching hypothesis that the Australasian dingo (*Canis (familiaris) dingo*) is evolutionarily intermediate between the wild wolf (*Canis lupus*) and domestic dogs (*Canis familiaris*) [3]. One alternate hypothesis is that the process of domestication is continual and does not proceed in a stepwise manner [4], instead representing a series of phases reflecting an intensification of the relationship between a wild animal (or plant) and human societies [5].

The taxonomic name of the dingo remains unstable, however, it is now clear the Australasian dingo is a distinct evolutionary lineage closely related to domestic dogs [6]. The first European drawing of an animal referred to as a "dingo" appears in White 1790 [7] with a more complete anatomical description appearing in Meyer 1793 [8]. A "large dog" from coastal eastern Australia near Sydney was earlier illustrated by George Stubbs in 1772, based on a recorded description by Joseph Banks from 1770; it is now clear that this animal was a dingo, but the name had not yet been learned from the local Aboriginal people. We follow the precedent that when zoologists disagree over whether a certain population is a subspecies or a full species, the species name may be written in parentheses. Scientists advocating a General

Lineage Species Concept consider dingoes to be distinct species (*Canis dingo*) or a subspecies of domestic dog (*Canis familiaris dingo*) [9-11]. Others advocating a Biological Species Concept [12] consider the dingo to be a breed of dog (*Canis familiaris* breed dingo) due to the interfertility between dingo and domestic dog [11, 13, 14].

Corbett [15] mentioned the possibility of three different dingo ecotypes existing in north, central and southeastern Australia. These are now referred to Tropical, Desert, and Alpine dingoes [16]. Subsequently, Corbett [17] noted that dingo skulls from southeastern Australia (Alpine dingoes) were genuinely different from those of the rest of the country, but posited the differences may be due to hybridization with domestic dogs rather than independent lineages. Jones [18] agreed that the southeastern dingoes, were distinct and suggested a revaluation of ecotype morphologies to resolve the conundrum.

Analyses of mitochondrial variation in canids from Southeast Asia supports the hypothesis that there are distinct dingo lineages [19-22]. Zhang et al. [19] found a strong Bayesian posterior value supporting the separation of Australian dingoes into two groups. One is a northwestern group, whereas the other is a southeastern group that clusters with New Guinea Singing dogs (*Canis (familiaris) hallstromi*). Support for two, or perhaps three, distinct lineages of dingoes has also come from Y-chromosome and SNP-chip data [23, 24].

The dog is the first species to be domesticated [25]. They are likely the most frequently kept domestic animal, exhibit exceptional levels of morphological variation, and many breeds have been developed by strong artificial selection in the past 200 years [26-28]. The Australasian dingo has been proposed to be a functional [29] and evolutionary [6] intermediate between wild wolves and domesticated dogs. Unfortunately, the absence of a dingo holotype reference specimen impedes our ability to definitively determine whether

dingoes are a tamed intermediate or a feral canid because we do not have a single reference point that links the scientific name to a specific specimen [30].

This study aims to link high resolution long-read *de novo* chromosomal assembly, mitochondrial DNA sequence and the DNA methylome with morphological descriptions of head shape and computed tomography data of brain data to describe the ‘archetype’ dingo (Fig. 1). This designation will support future comparisons with a reference enabling further characterization of the evolutionary history of the dingo. In this case we do not propose any formal taxonomic name for the specimen as it is a regional morphotype that is being characterized however we suggest the principle of having a ‘type’ specimen makes biological sense and will enable the focusing of future research.

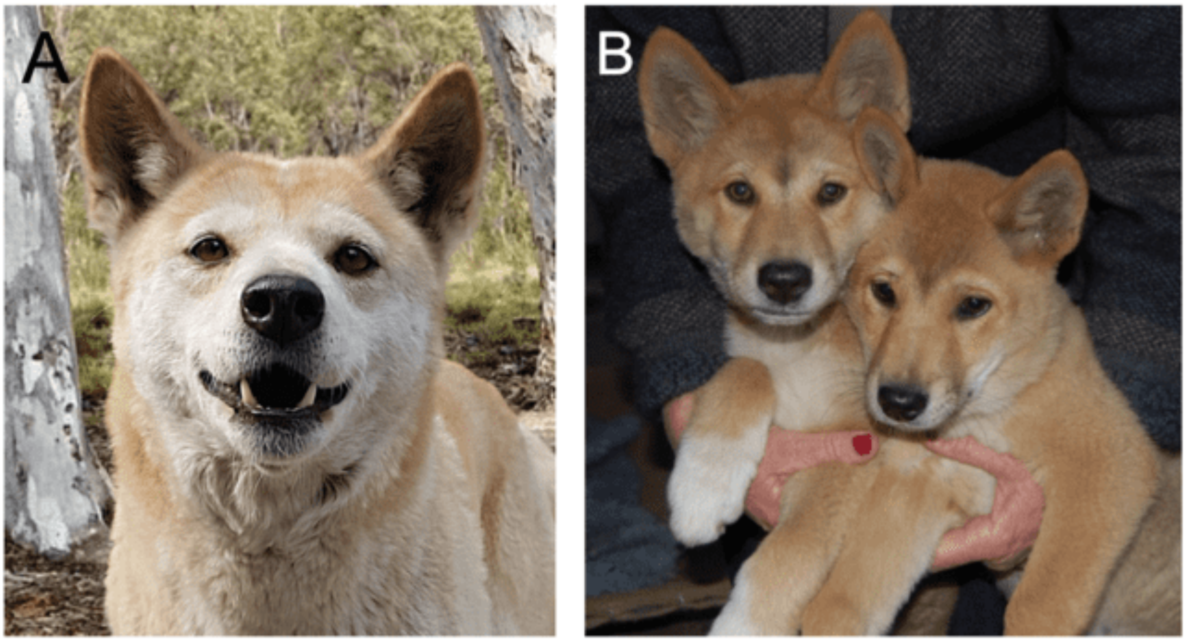

Figure 1 title: Cooinda the dingo.

Figure 1 legend: The genomic and morphological data in this study is based upon a single individual named Cooinda from Dingo Sanctuary Bargo in the southern highland region of New South Wales. Based on her parentage, broad skull, and stocky appearance the Sanctuary considers her an Alpine dingo. We compare her with other dingoes found in southeastern

Australia and with those found in the center and northwest of the continent including Desert dingo Sandy [6]. (A) Dingo Cooinda as an adult female. (B) Brother Typia (RHS) and Cooinda (LHS) as 8-week-old puppies.

Results

Chromosome-level genome assembly

Workflow

The genome was assembled following a similar pipeline to Field et al. [28] (Supplementary Fig. 1). Briefly, 1722 contigs were assembled from SMRT and ONT sequence data with a total length of 2.38 Gb and N50 length of 12.4 Mb [31]. The contig assembly was then polished for two rounds with SMRT reads, correcting ~5 million bases in the first round and ~15 thousand in the second [32, 33]. The assembled sequence contigs were scaffolded sequentially using 10X linked-reads and polished with 10X linked-reads [33]. The scaffolded assembly was then super scaffolded with Bionano and Hi-C proximity ligation. Supplementary Fig. 2 shows the contact matrices generated by aligning the Hi-C data set to the genome assembly after Hi-C scaffolding [34, 35]. To increase the contiguity of the assembly we used the SMRT and ONT reads to fill gaps, which was then followed by a final round of SMRT read polishing. The gap filling successfully closed 282 gaps increasing contig N50 to the final figure of 23.1 Mb. A final round of polishing was performed with 10X linked reads. The resulting chromosome-length genome assembly and its gene annotation was deposited to NCBI with accession number GCA_012295265.2.

Assembly statistics and completeness

The final assembly had a total length of 2,398,209,015 bp in 477 scaffolds with a scaffold and contig N50 of 64.8 Mb and 23.1 Mb, respectively (Table 1). Chromosome-level scaffolds

accounted for 98.4 % of the assembly with only 0.9 % (21.1 Mb) of all sequences not aligning to a CanFam4.1 chromosome [36].

Evaluation by Benchmarking Universal Single-Copy Orthologs (BUSCO v5.2.2 [37]) against Carnivora_odb10 data set (n=14,502) indicated that 95.1 % of the conserved single-copy genes were complete (Table 1**,** Supplementary Fig. 3A). Only 3 of 13,791 complete (single-copy or duplicated) BUSCO genes were not on the 39 nuclear chromosome scaffolds.

Next, we compared single-copy "Complete" BUSCO genes in Alpine dingo Cooinda and nine canid genomes [6, 27, 28, 36, 38-41]). Of the 13,722 genes, 13,711 were found in the assembly using BUSCOMP v1.0.1. Only Sandy the Desert Dingo v2.2 (13,715 genes) and China the Basenji v1.2 (13,712 genes) had more.

Additional kmer analysis of the final assembly [42] yielded 97.32 % (97.2% in chromosomes) and an overall Q-score estimate of 37.5 (38.4 for chromosomes). No sign of retained haplotigs was evident (Supplementary Fig. 3B).

Table 1: Genome assembly and annotation statistics for Alpine dingo (Cooinda) vs Desert dingo assembly (Sandy)

**Statistic**	**Alpine dingo**	**Desert dingo**
Total sequence length	2,398,209,015	2,349,862,946
Total ungapped length	2,390,794,485	2,349,829,267
Number of contigs	802	228
Contig N50	23,108,747	40,716,615
Contig L50	36	20
Number of scaffolds	477	159

Scaffold N50	64,752,584	64,250,934
Scaffold L50	15	14
Number of gaps	325	69
BUSCO complete (single/ duplicate copy)	95.1% (S: 92.7% D:2.4%)	95.3% (S: 92.9% D:2.5%)
BUSCO fragmented	0.8%	0.8%
BUSCO missing	4.1%	3.8%

Comparison of dingo genomes

We generated a Circos plot [43] to represent the single-nucleotide variants (SNV) and small indel variation between the Alpine and Desert dingo (Fig. 2) using MUMmer4 [44], and sniffles v1.0.11 [45]. In comparison to the autosomes, these plots show low variation on the X chromosome (Fig. 2). To further investigate the low variation, we compared each of the dingoes to CanFam4 (Supplementary Fig. 4, Supplementary Table 1). We then generated a conservative consensus set of structural variants (SV) by merging PacBio, and Nanopore SV calls generated with sniffles [45, 46]. Overall, we found ~half the number of SV and small variants calls relative to Desert dingo than to CanFam4 (32798 v 62524 and 1729790 v 3839712, respectively).

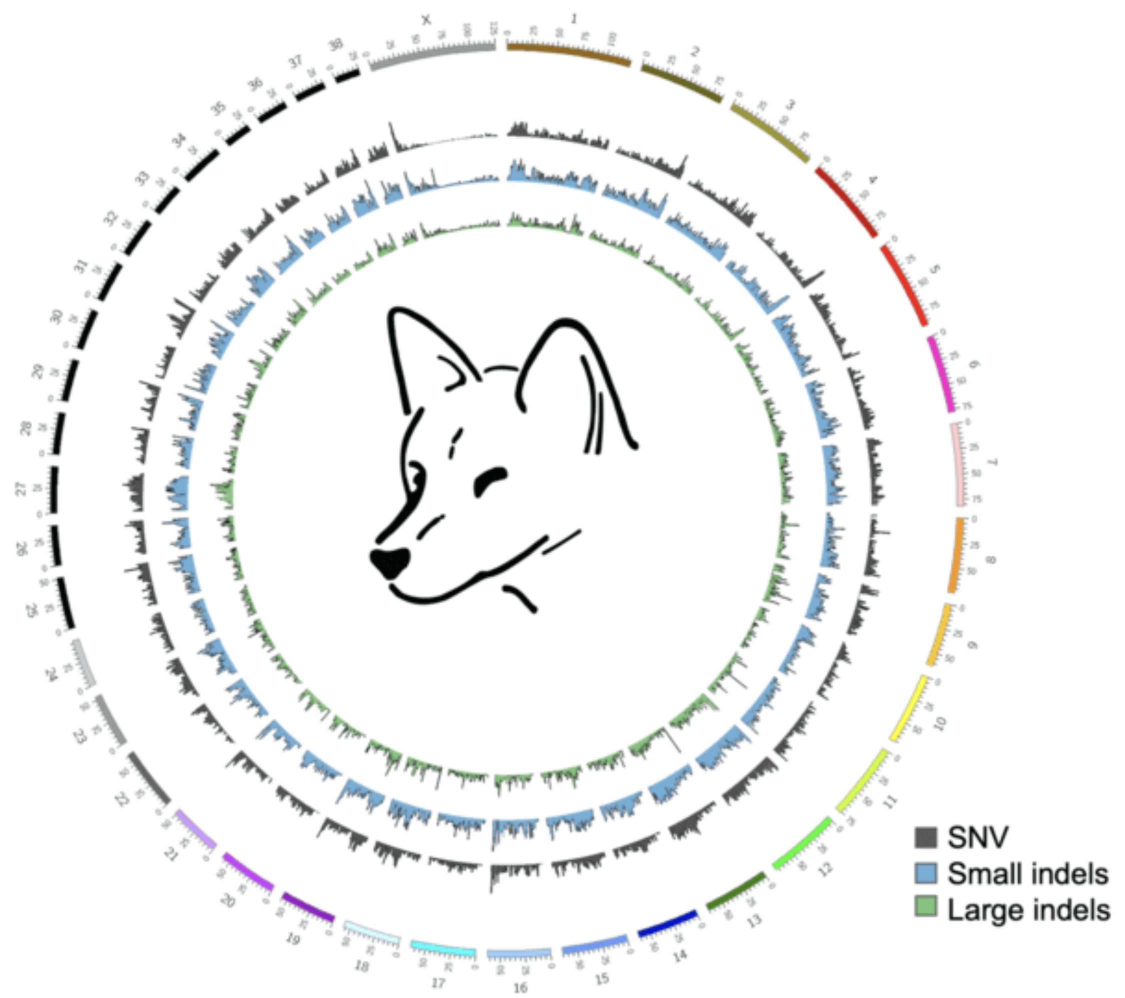

Figure 2 title: Circos plot comparing Alpine and Desert dingo genomes

Figure 2 legend: Plot compares the 38 autosomes and X chromosome of the Alpine and Desert dingo. The plot shows the low variation on the X chromosome compared to the autosomes.

We generated synteny plots using MUMmer plot and GenomeSym [47]. Synteny plots between the dingo genomes show several large-scale chromosomal events. On chromosome 16 there is a 3.45Mb inverted region and a 0.9Mb complex rearrangement (Supplementary Fig. 5). This 3.45Mb inversion does not appear in the wolf or domestic dogs, so we speculate it is unique to the Desert Dingo assembly [6]. The inversion overlaps 60 unique ENSEMBL transcripts and was enriched for gene ontology terms of cellular metabolic processes,

including glycolysis and glucose metabolism [6]. Also, on Chromosome 16, the 0.9Mb complex rearrangement occurs between 55 – 57 Mb downstream (Supplementary Fig. 5). Additional structural events include small inversions on Chromosome 11 and on Chromosome 25 (Supplementary Fig. 5). On the X chromosome, there appear to be multiple small nonsyntenic regions (Supplementary Fig. 5); however, further examination of these apparent differences is required to establish whether they are true biological differences or assembly artifacts.

In parallel, we used GeMoMa gene predictions [48] to investigate chromosomal level events. Like the synteny analyses, this approach revealed a large inversion and a disordered region on chromosome 16 as well as smaller inversions on Chromosomes 11 and 25. We also found two structural events on chromosome 26 (Supplementary Fig. 6) containing mostly short genes that are not perfectly conserved (Supplementary Fig. 5F). A MUMmer4 nucmer alignment plot [44] for chromosome 26 corroborated these events (Supplementary Fig. 6)

The Alpine and Desert dingo both have a single copy pancreatic amylase gene (AMY2B) on Chromosome 6. The Alpine dingo assembly does not include a 6.4kb long LINE that was previously reported in the Desert dingo [6].

Phylogenetic analyses

All 39 full-length chromosomes in the final assembly were aligned to the corresponding chromosomes in nine published canine *de novo* genome assemblies [6, 27, 28, 36, 38-41]). SNVs and small indels (deletions and insertions <50bp) were called using MUMmer4 call-SNPs module for all possible pairings (Supplementary Table 2). Distance matrices were generated from the inter-canid differences in SNVs and indels and then transformed to WA distance [6, 49]. Fig. 3AC show the phylogenetic tree from SNVs and indels respectively. Both figures show strong support for monophyly of dingoes and dogs relative to the wolf.

These figures also strongly support the hypothesis that dingoes are the sister group to domestic dogs. Fig. 3BD show the ordination analyses from SNVs and indels, respectively. Scores for the taxa calculated from the largest two axes (Axis 1 and Axis 2) describe 75.6% of the variance in SNV's and 73.2% of the variance in indels (Fig. 3BD).

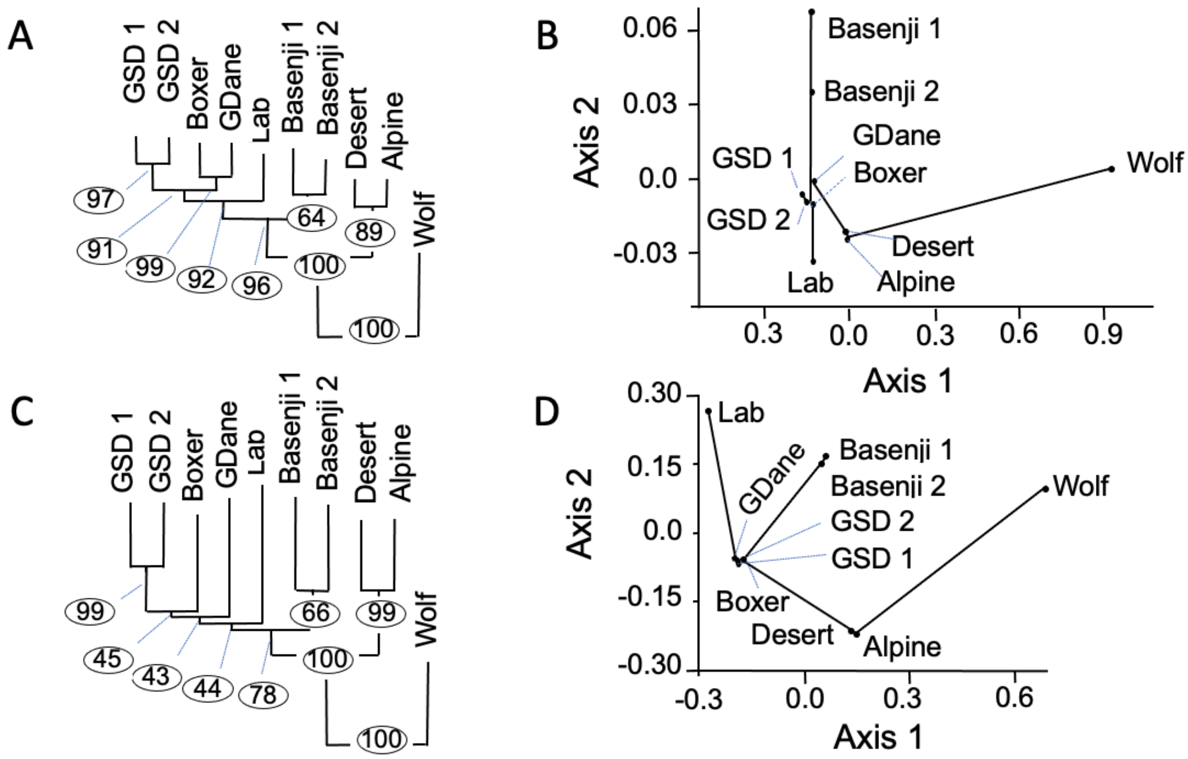

Figure 3 title: Phylogenetic and ordination analyses of nuclear DNA from SNVs and indels from 10 canines.

Figure 3 legend: (A) Phylogenetic tree from SNVs. Branch length proportional to the number of changes and bootstrapping percentage in circles. (B) Ordination analyses from SNVs showing first two axes from non-metric multidimensional scaling. (C) Phylogenetic tree from indels. Branch length proportional to the number of changes and bootstrapping percentage in circles. (D) Ordination analyses from indels showing the first two axes from non-metric multidimensional scaling. Abbreviations: Lab – Labrador; GSD – German Shepherd Dog; GDane – Great Dane; Wolf — Greenland wolf

Mitochondrial genome

Genome assembly workflow

A 46,192 bp contig from the assembly mapped onto the CanFam reference mtDNA. It constituted a repeat of approximately 2.76 copies of the mtDNA. Following additional polishing and circularization, a final 16,719 bp mtDNA genome was extracted and has been uploaded to GenBank (OP476512).

Comparison of dingo mtDNA genomes

When the mtDNA genome of Alpine dingo Cooinda is compared with that of Desert dingo there is a single 10bp SV in the control region that highlights the repeat number difference. In the former, there are 28 repeats (RCGTACACGT) ACGTACGCGCGT, while in the latter, there are 29. Potentially the R(G or A) could represent heteroplasmy [50] that may be further studied with single cell sequencing approaches [51]. Folding this region [52] shows that increasing repeat number increases stem length and overall stability (Supplementary Fig. 7).

Next, we conducted a network analysis in Popart [53] to determine whether the mtDNA of dingo Cooinda fell within the previously described dingo southeastern or northwest clade (Fig. 4) [19, 22]. We included dingo mtDNA from four previous studies, a New Guinea Singing Dog, and an ancient Iron Age dog from Taiwan [6, 22, 54-56]. There were 89 segregating sites and 32 parsimony informative sites in the dataset. Predictably, there were no differences between the mtDNA genome of Cooinda and that previously published from her brother Typia [54]. Further, as expected, Cooinda and Typia mtDNA clustered with samples that had previously been collected from the Alpine region (Fig. 4). Somewhat unexpectedly, the mtDNA from Sandy the dingo found in the desert [6] did not cluster with dingoes from the northwest clade but was closer to canids in the southeastern clade (Fig. 4). This

relationship could imply the introgression of Alpine alleles into the Sandy genome however further work would be needed to confirm this.

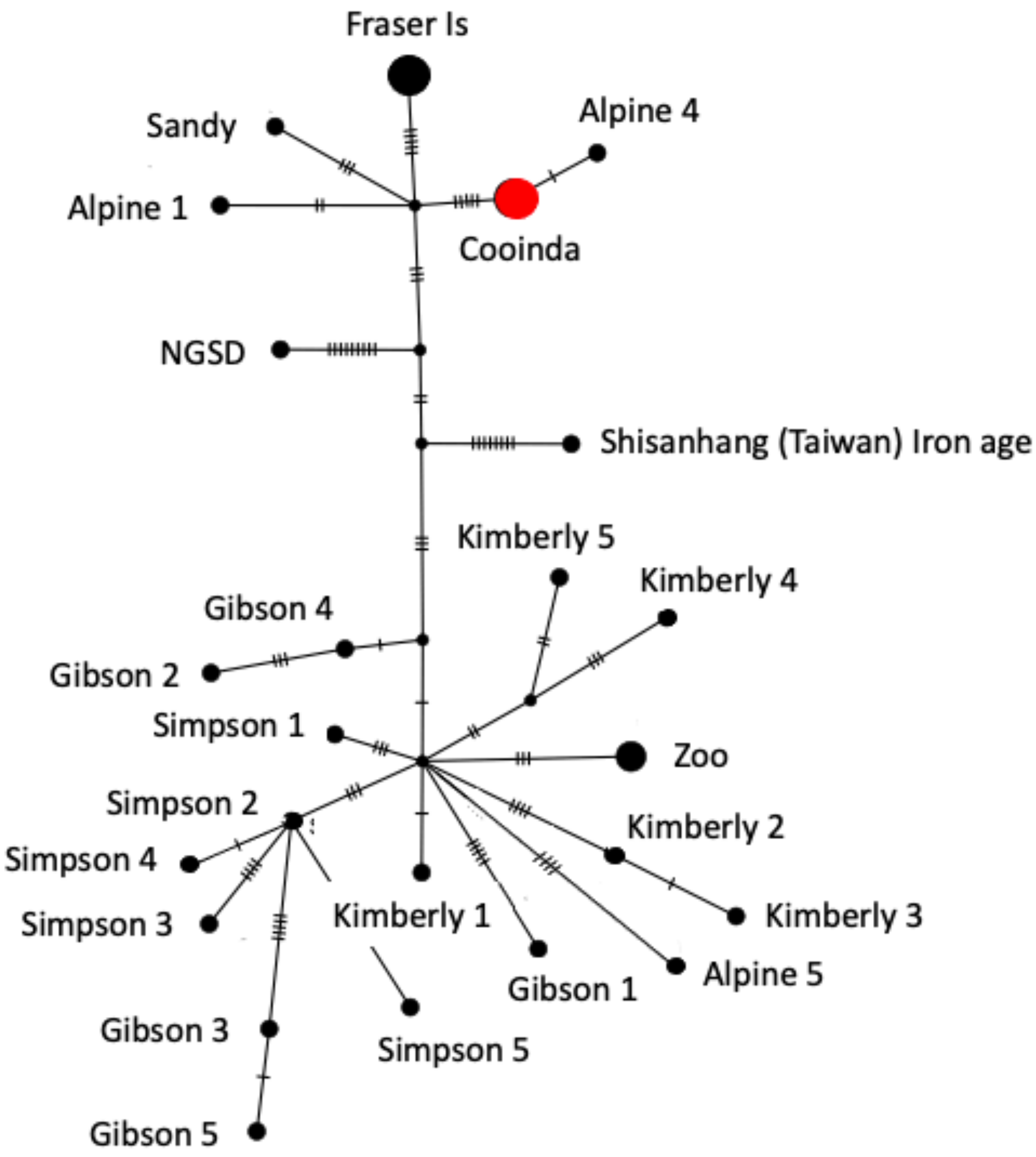

Figure 4 title: Neighbor-joining network analysis from mtDNA.

Figure 4 legend: The size of the circle represents the number of identical sequences and small cross lines the number of SNPs on each branch. The analyses show that dingo Cooinda

is in the southeastern clade. Cooinda represents Alpine dingo Cooinda sequenced here, as well as Alpine 2, Alpine 3 [22], MH035670 [55], and Typia [57]. Fraser Is represents the Fraser Island 1-5 samples [22]. Zoo represents three dingoes from the New Zealand Zoo [55]. Shisanhang (Taiwan) is one of two samples from the region and is considered the root of the network [19].

DNA methylome

To explore the regulatory landscape of dingo Cooinda, we performed whole genome bisulfite sequencing [58] on genomic DNA extracted from whole blood. In concordance with other adult vertebrates [59, 60], the Cooinda genome displays a typical bimodal DNA methylation pattern. Over 70% of CpG dinucleotides are hypermethylated (levels higher than 80%), and 5% of CpG dinucleotides hypomethylated (methylated at 20% or lower) (Supplementary Fig. 8A).

Next, to determine the number and genomic distribution of putative regulatory regions, we segmented the methylome into unmethylated regions (UMRs) and low-methylated regions (LMRs) using MethylSeekR [61]. UMRs are fully unmethylated and largely coincide with CpG island promoters, whereas LMRs display partial DNA methylation, characteristic of distal regulatory elements such as enhancers in other mammalian models [62]. MethylSeekR analysis identified ~ 19,000 UMRs and ~44,000 LMRs in line with previously reported numbers of promoters and enhancers (e.g., human: ~18,000-20,000 UMRs and 40,000-70,000 LMRs; mouse: ~17,000-19,000 UMRs and 55,000-90,000 LMRs) [61, 63] (Supplementary Fig. 8BC).

To establish whether proximal gene regulatory regions in the dingo Cooinda genome display different methylation states in the Desert dingo, we converted Cooinda UMR coordinates

from Cooinda to the Desert dingo genome assembly using LiftOver (see Methods). Next, we calculated average DNA methylation at Cooinda UMRs and their corresponding lifted-over regions in the Desert dingo genome. We found two UMRs in the Cooinda dingo were hypermethylated in the Desert dingo. These regions overlapped gene bodies of glucagon receptor gene GCGR and histone deacetylase HDAC (Supplementary Fig. 8DE). GCGR is on chromosome 9 and has a single transcript. This transcript is 99.8% identical at the amino acid level between the dingoes. HDAC4 occurs on chromosome 25 and has 12 transcripts with all 12 transcripts being 100% identical at the amino acid level. Further studies are needed to determine the functional significance of the observed differences in DNA methylation. Altogether, this data provides a genome-wide resource for the putative gene regulatory regions in the Alpine dingo genome, which will be instrumental for future studies.

Morphology

Skull Morphometrics

Cranial morphology (Supplementary Fig. 9A), quantified using 3D geometric morphometric landmarks, is that of a typical adult female Alpine dingo (Fig. 5). Within the morphospace defined by the principal components explaining the greatest variation between specimens (PC1, PC2), dingo Cooinda's position is clearly within the Alpine cluster (Fig. 5A). Alpine and Desert dingoes are most clearly differentiated from one another along PC1 (15.70%), for which increasing values describe crania with relatively shorter and broader rostra, shallower orbitals with broader zygomatic arches at the glenoid fossa, prominent and anteriorly-positioned frontals, a higher cranial vault, and prominent sagittal cresting tending to terminate in a high, posteriorly-positioned occiput (inion). Positive values along PC2 (10.60%) mainly denote relatively gracile crania with posteriorly-angled frontals, poorly-developed sagittal cresting, downward-sloping posterior calvarium and a low occipital

termination. The sampled Alpine and Desert groups exhibit a near-identical range of PC2 values. As the development of the sagittal cresting, calvarium shape and occipital prominence are related to age and sex, with these traits tending to be more robust and well-developed in males and older dingoes [64], the shared PC2 values across Alpine and Desert groups likely reflect related demographic variation within the respective populations. Within each population (Alpine, Central Desert, Western Desert), males and females overlapped in in their position along PC2 (Supplementary Fig. 9), indicating an absence of strong dimorphism associated with the major axes of shape variance. Despite considerable overlap, PC2 scores tended to be lower in females compared to males in the Alpine and Western Desert populations (see Supplementary Fig. 9, Supplementary Table 3).

The regression of cranial shape (Procrustes shape variables) on log centroid size (Procrustes shape variables ~ log(centroid size)) revealed that size contributed significantly to shape variance in the sample (3.91% variance, $p < 0.001$). Size was found to have a non-significant effect on the morphological trajectory described by PC1, which separates Alpine and Desert dingo populations (Fig. 1C), with only 1.23% of related shape-change predicted by centroid size ($p = 0.124$). Conversely, size predicted 19.88% of shape-change associated with PC2 ($p < 0.0001$). Alpine and Desert dingo populations share overlapping scores along PC2, and variation along this axis reflects intra-population variability in demographic makeup (age, sex) that should be expected within a natural population. As such, size differences play very little to no role in determining Cooinda's morphological relationship to Desert dingoes but are important to her position in the Alpine group (Supplementary Fig. 10BC). The low proportion of variation captured in each principal component is a previously-noted feature of the dingo cranial landmark dataset [65] and is unrelated to allometry.

Brain imaging

To supplement the morphological data, we quantified brain size. Using a thresholding approach, we used the software 3D Slicer [66] to segment the whole brain as the region of interest. Despite the canids being of very similar size the dingo brain ($75.25 cm^3$) was 20% larger than the dog brain ($59.53 cm^3$) (Fig. 5B).

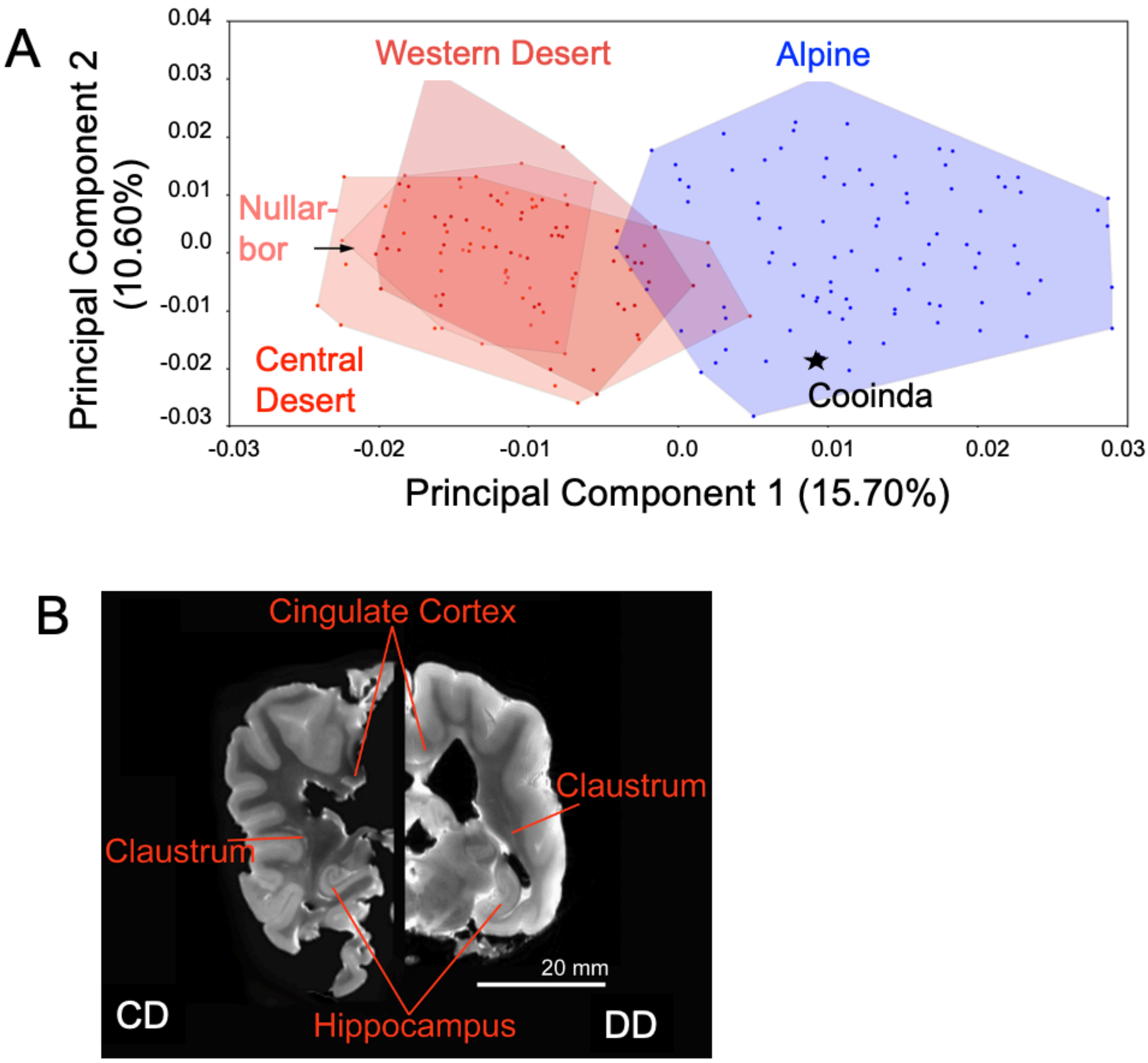

Figure 5 title: Morphometrics and brain image of Cooinda from the Bargo Dingo Sanctuary, NSW, Australia.

Figure 5 legend: (A) Principal Component ordination of geometric morphometric cranial shape data indicating Cooinda's position in relation to Alpine and Desert dingoes. Blue represents Alpine dingoes, and the red hues indicate dingoes from different Deserts that are broadly overlapping. Dingoes from the Nullarbor overlap most with those from the Alpine region. There is no overlap of dingoes from the Central desert with Alpine dingoes.(B) Brain image, showing a hemispheric comparison of slices generated by Magnetic Resonance (MR) imaging of Cooinda dingo (CD) and a similar-sized domestic dog (DD).

Discussion

Domestication has received much attention from diverse fields, reflecting the complexity of the process and variation in its duration and intensity [5]. A notable gap in our understanding of the principles of domestication has been the identification of a model system to test Darwin's two-step predictions [2]. Here we provide the necessary groundwork to explore the potential for dingoes to be a functional and evolutionary intermediate between wild wolves and domestic dogs. One alternate hypothesis is that the process of domestication does not proceed in a stepwise manner [4], but is continual process that represents an intensification of the relationship between a wild species and humans [5].

In this study we compare our high-quality chromosome-level *de novo* assembly of the dingo Cooinda genome with that of the Desert dingo [6], seven domestic dogs [27, 28, 36, 38-40] and the Greenland Wolf [41]. Relative to the wolf and the domestic breeds the Australasian dingo ecotypes are monophyletic. Future studies may include ancient dingo and south east Asian specimens [3], the New Guinea Singing dog [4] and Chinese indigenous dogs [4]. Ancient specimens have potential to give insight into the evolutionary history of dingoes [3] and further instruct the influence of domestic dog admixture [17]. New Guinea Singing Dog may be the sister group to a monophyletic dingo lineage or perhaps more closely related to

the Alpine ecotype as suggested by the mtDNA network analyses [19] and cranial shape studies [65]). Inclusion of Chinese indigenous dogs will facilitate determination of the relationships among crown domestic dog breeds [4] and thereby facilitate determination of the divergence date of dingoes and modern dogs.

Multiple large scale chromosomal inversions occur between the two dingo assemblies. There are two large rearrangements on chromosome 16 and likely structural events on Chromosomes 11, 25 and 26 (Supplementary Figs 7, 8). It is also possible that there are multiple small inversions on the X chromosome. It is important to determine the frequency of these events and whether breakpoints affect any regulatory regions or protein coding genes. Inversions may maintain locally adapted ecotypes, while breakpoints may disrupt regulatory regions or protein coding genes. Hager et al. [67] discovered a 41-megabase chromosomal inversion that characterized defining traits of deer mice (*Peromyscus maniculatus*) and implicated divergent selection in maintaining distinct ecotypes in the wild despite high levels of gene flow. An inversion disrupting FAM134b has been associated with sensory neuropathy in Border Collie dogs [68].

There is a single copy of AMY2B in both dingo genomes; however, they differ by a 6.4 kb retrotransposon insertion present in the Desert dingo. As the retrotransposon is absent in the Greenland wolf and Alpine dingo it would seem likely that the retrotransposon has inserted into the Desert dingo and domestic dog lineages independently. LINE elements can generate duplications through an RNA intermediate and have been associated with amylase expansions in a range of species from humans to mice and rats to dogs [69, 70]. A 1.3kb canid-specific LINE element in domestic dogs is associated with each amylase copy [70]. This expansion is predicted to increase the ability to digest starch [6, 71]. Field et al. [28] compared the influence of *AMY2B* copy number on the microbiomes of dingoes and German

Shepherd dogs. They observed distinct and reproducible differences that they hypothesized may influence feeding behaviors. Further studies on *AMY2B* may be fruitful as copy number may be an ecologically relevant mechanism to establish the role of a canid in the ecosystem.

Both dingo ecotypes exhibited low variation on the X chromosome, although it could be argued that variation along the chromosome is not uniform (Fig. 2). Theoretical models predict that genes on the X chromosome can have unusual patterns of evolution due to hemizygosity in males. Sex chromosomes are predicted to exhibit reduced diversity and greater divergence between species and populations compared to autosomes due to differences in the efficacy of selection and drift in these regions [72, 73]. In canids, Plassais et al. [74] show genetic variation in three genes on the X chromosome is strongly associated with body size. Further studies of genetic variation of genes on the X chromosome within and between ecotypes are likely informative.

We integrate the mtDNA genome assembly data with that previously collected from 29 canids in Australasia [6, 22, 54-56]. The mitochondrial genome has been used to infer historical events in various species including canids, but the D-loop region has been difficult to align. Here we show that the region can be folded to increase structural stability with repeat number (Supplementary Fig. 8AB**).** We found 28, 10-bp repeats in dingo Cooinda compared to 29 in the Desert dingo. The function of the proposed structures is unknown. Still, folding the region into an extended repeat-dependent stem is expected to decrease the time the DNA in the D-loop is single-stranded during replication. More speculatively, the structure may have a regulatory function that influences mitochondrial bioenergetics and the evolution of mtDNA [75]. Björnerfeldt et al. [76], found that domestic dogs have accumulated nonsynonymous changes in mitochondrial genes at a rate faster than wolves implying a relaxation of selective constraint during domestication.

Phylogenetic and network analyses show that dingo Cooinda has the dingo southeastern Australian mtDNA type of the canine A1b4 subhaplogroup. This southeastern type has been proposed to originate in southern China and includes dogs from Papua New Guinea [19, 22]. Based on mtDNA data, Zhang et al. [19] propose that the TMRCA for most dingoes dates to 6,844 years ago (8,048–5,609 years ago). This estimate is about 3,000 years older than the first known fossil record [77] suggesting that at least two dingo mtDNA haplotypes colonized Australia or older fossil records of dingoes in Australia have yet to be found.

Next, we compare the regulatory landscape of Cooinda dingo with that previously published for the Desert dingo. In comparison to the Alpine dingo, the glucagon receptor gene GCGR and HDAC4 are hypermethylated in the Desert dingo suggesting the potential for dietary or immune differences between ecotypes. Highly methylated gene promoters often indicate a transcriptionally repressed state, while unmethylated gene promoters specify a permissive state [78]. Field et al. [6] previously proposed differences in the feeding behavior of dingoes and wild dogs linked to their *AMY2B* copy number. GCGR is activated by glucagon and initiates a signal transduction pathway that begins with the activation of adenylate cyclase, which in turn produces cyclic AMP. Glucagon is considered the main catabolic hormone of the body and is central to regulating blood glucose and glucose homeostasis [79]. In mice, glucagon has anti-inflammatory properties [80]. HDAC4 is a member of the ubiquitously important family of epigenetic modifier enzymes and has been implicated in processes related to the formation and function of the central nervous system and metabolism. HDAC4 acts as a regulator of pattern-recognition receptor signaling and is involved in regulating innate immune response [81]. In humans, mutations in HDAC4 have been linked with eating disorders [82]. Overlapping conserved Nanopore/PacBio structural variants with these genes identified no variants within GCGR and a single 35bp intronic insertion in HDAC4. The functional impact (if any) of this insertion is unknown.

Dingo Cooinda's cranial morphology is consistent with the Alpine ecotype from the 20th century. As the first cranial morphological assessment of an Alpine dingo considered to be "pure" by genomic verification, this result is significant in that it suggests that the phenotypic distinctiveness of Alpine dingoes from Desert dingoes is not exclusively the result of recent domestic dog ancestry. Dog admixture has been the predominant explanation given [83] primarily based on the fact that such ancestry is relatively enriched in the southeast region of Australia compared to the north and west [84, 85]. An alternative explanation is that the Alpine and Desert dingoes represent distinct evolutionary lineages. Koungoulos [65] suggested that the cranial shape of Alpine and other southeastern dingoes shares broad similarities with that of New Guinea Singing Dogs and is distinct from the more widespread northwestern lineage [22]. However, these two scenarios are not mutually exclusive. Most introgression likely occurs when a female dingo mates with a male domestic dog. In such cases, extensive backcrossing will not exclude the domestic dog Y. Therefore, examining the Y chromosome of males shown to be pure with the current battery of nuclear-encoded microsatellites will illuminate genetic history. A combination of direct radiocarbon dating, genetic sequencing and morphometric assessment for subfossil material will provide a more confident picture of the nature of change or continuity between ancient and modern Alpine dingoes.

Finally, we supplement our morphological data with magnetic resonance and computed tomography data of Alpine dingo Cooinda's brain. Her brain was 20% larger than the similarly sized domestic dog, which is consistent with the hypothesis that she was tamed but not domesticated [3] (Fig. 1C). Our brain imaging data are also compatible with prior comparisons that have used endocranial volume as a proxy for brain size, examining a small sample of dingoes (see Geiger et al. [86]) compared to wolves, domestic, basal and archaeological dogs [3]. Endocranial volume in a mixed sample of domestic dogs was shown

to be around 30 cm^3 smaller than in wolves and jackals [87, 88], which is greater than the 15.7 cm^3 difference between the brains of Cooinda and the domestic dog sampled here. Similarly, brain mass has been shown to be 28.8% smaller in a broad sample (>400) of domestic dogs as compared to wolves [87, 89], which also places the 20% difference between Cooinda and the domestic dog as less pronounced than is seen for comparisons with the wild counterpart (wolf). Brain size reductions are common among domesticated animals compared to their wild counterparts, having been observed across many species, including sheep, pigs, cats, and dogs [87, 90]. Smaller-sized brains, especially size reductions in regions of the forebrain involved in the fight-or-flight response, have been associated with tameness and reductions in fear-based response among domestic animals compared to wild animals [91]. These changes have also been linked to potential reductions in cognitive processing requirements associated with inhabiting anthropogenic environments with lower complexity [92, 93]. Moreover, brain size reductions appear to persist where domestic animals have re-entered a wild environment and exist as feralized animals, at least under certain circumstances [94-96], suggesting that prolonged past exposure to the human niche may be detectable in brain traits. An alternative hypothesis is that differences in brain size is due to environmental adaptation or perhaps Cooinda was an anomaly. Examination of brain size may represent a fruitful pathway for further investigation determining the status of the dingo as a potential feralized animal.

There are at least three possible explanations supporting the existence of two dingo ecotypes (Alpine and Desert). The first is they are ancient Asian lineages that have come into sympatry in Australia. One alternate hypothesis is that a single lineage spread through southeast Asia and then diverged in Australia. There are no major geographical divides in continental Australia, suggesting any differences may reside at the level of biological interactions or they are influenced by climate. In the former case, one possibility is that one or more inversions

may maintain the ecotypes [67]. An intriguing alternate hypothesis is that responses to parasites or venomous animals may occur if there are genetic differences in the responses of the ecotypes. In Nigeria, population genomic analyses of 19 indigenous dogs identified 50 positively selected genes including those linked immunity that likely involve adaptations to local conditions [97]. Experimentally it has been shown that adaptation to different parasites or snakes can influence the invasion success of three-spined sticklebacks (*Gasterosteus aculeatus*) and may represent a barrier to gene flow, even between closely related connected populations [98]. In Australia, various parasites and venomous animals have broadly similar distributions to the Alpine ecotype, such as the paralysis tick (*Ixodes holocyclus*) and the red-bellied black snake (*Pseudechis porphyriacus*) [99].

Conclusions

Here we characterize dingo Cooinda and propose that she be considered the archetype for Australasian dingoes. Characterizing an archetype opens potential for testing Darwin's [2] two-step model of domestication as an alternative to the hypothesis that domestication represents a continuum [5]. Under the scenario that the dingo has been unconsciously selected, we predict genomic signatures of tameness, as an outcome of unconscious selection [100-102]. Morphologically, we predict lowest shape variation in the rostrum and facial skeleton in the wolf (natural selection), intermediate in the dingo (unconscious selection) and highest in domestic breeds (artificial selection) (i.e., rank order wolf< dingo < modern breeds). Wild populations are more likely to show a narrow range of shape variation about a fitness optimum, whereas changed environmental conditions could support and promote the survival of forms that are farther from the adaptive peak. This is evidenced by earlier research that has shown cranial morphological variation in domestic dogs exceeds that exhibited by the Order Carnivora [26]. In terms of brain size, we predict

the magnitude of relative brain size difference will be greater between dingoes and modern breeds than between wolves and dingoes (i.e., rank order wolf> dingo >> modern breeds). Brain size reduction is pronounced in artificial selection and associated with the lack of fear avoidance behavior in domesticates [103]. Dingoes do not show domesticate level reductions in ‘fight or flight’ response [29], and our initial data appear to be at least consistent with this based on the relative brain volume we report.

Methods

Sampling: Cooinda the dingo

In selecting an animal for the project, it was considered essential to select an individual that represented the Alpine ecotype, which is found around Sydney, New South Wales (NSW). The individual selected was bred at the Dingo Sanctuary Bargo, NSW, approximately 100km west of Sydney, and has been included in multiple previous studies [6, 29]. Cooinda is the litter sister to Typia from whom short read data had previously been obtained [54]. Cooinda’s parents (Mirri Mirri and Maka), her brothers Typia and Gunya and her were all ginger in color and determined to be pure by microsatellite testing [104]. Mirri Mirri and Maka were independently found in the Alpine region of New South Wales.

An aim of the study is to link genetic and morphological variation, so we provide a brief description of her here. As is typical of Alpine dingoes Cooinda was stocky in appearance with a brad skull and prominent eyes. She was light ginger in color, with dark brown eyes with white paws and chest (Fig. 1AB). Her double coat was not oily like many modern breed dogs and did not have a dog-like odor when wet. She had a pointed muzzle with a broad skull and hooded erect ears. She could turn her neck 180 degrees in any direction. She had lean muscular legs with a long bottle-shaped bushy tail. She weighed 22kg and stood 46cm at the

withers. She did not have dewclaws and came into estrus annually. Dingo Cooinda had a loud and clear howl and did not have a modern-dog bark [105]. Cooinda died in 2019 at 10 years of age.

Chromosome-level genome assembly

DNA extraction and sequencing

Genomic DNA for the Pacific Bioscience Single Molecule Real-Time (SMRT) sequencing was prepared from 2 mL of fresh blood using the genomic-tip 100/G kit (Qiagen, Hilden, Germany). This was performed with additional RNase (Astral Scientific, Taren Point, Australia) and proteinase K (NEB, Ipswich, MA, USA) treatment following manufacturer's instructions. Isolated gDNA was further purified using AMPure XP beads (Beckman Coulter, Brea, CA, USA) to eliminate sequencing inhibitors. DNA purity was calculated using a Nanodrop spectrophotometer (Thermo Fisher Scientific). Molecular integrity was assessed by pulse-field gel-electrophoresis using the PippinPulse (Sage Science) with a 0.75% KBB gel, Invitrogen 1kb Extension DNA ladder and 150 ng of DNA on the 9hr 10-48kb (80V) program. SMRTbell libraries with 20kb insert size were CLR sequenced on Sequel I machines with 2.0 chemistry. Sequencing included 18 SMRT cells with a total polymerase read length 94.25 Gb.

DNA for the Oxford Nanopore (ONT) PromethION sequencing DNA (1 µg) was prepared for ONT sequencing using the 1D genomic DNA ligation kit (SQK-LSK109, ONT) according to the standard protocol. Long fragment buffer was used for the final elution to exclude fragments shorter than 1000 bp. In total, 119 ng of adapted DNA was loaded onto a FLO-PRO002 PromethION flow cell and run on an ONT PromethION sequencing device (PromethION, RRID:SCR_017987) using MinKNOW (18.08.2) with MinKNOW core (v1. 14.2). Base-calling was performed after sequencing with the GPU-enabled guppy basecaller

(v3.0.3) using the PromethION high accuracy flip-flop model with config 'dna_r9.4.1_450bps_hac.cfg'.

For the 10X Genomics Chromium sequencing, DNA was prepared following the protocol described above for SMRT sequencing. A 10X GEM library was barcoded from high-molecular-weight DNA according to the manufacturers recommended protocols. The protocol used was the Chromium Genome Reagent Kits v2 (Document # CG00043 revision B). QC was performed using LabChip GX (PerkinElmer, MA, USA) and Qubit 2.0 Flurometer (Life Technologies, CA, USA). The library was run on a single lane of a v2 patterned flowcell. Sequencing was performed in 150bp paired-end sequencing mode on a single lane on the Illumina HiSeq X Ten platform with a version 2 patterned flowcell.

For the Bionano optical mapping high molecular weight (HMW) DNA was isolated from fresh blood (stored at 4°C) using the Bionano Prep Blood DNA Isolation Protocol following [28]. HMW DNA (~190 ng/μL) was labelled (BNG, Part #20351) at DLE-1 recognition sites, following the Bionano PrepTM Direct Label and Stain Protocol (BNG, Document #30206 revision C). Labelled DNA was loaded directly onto Bionano Saphyr Chips (BNG, Part #20319), without further fragmentation or amplification, and imaged using a Saphyr instrument to generate single-molecule optical maps. Multiple cycles were performed to reach an average raw genome depth of coverage of 180X.

For the Hi-C sequencing the assembly was scaffolded to chromosome-length by the DNA Zoo following the methodology described here: www.dnazoo.org/methods. Briefly, an *in situ* Hi-C library was prepared [106] from a blood sample of the same female and sequenced to 29X coverage (assuming 2.6 Gb genome size).

Workflow

For the initial assembly, The SMRT and ONT reads were corrected and assembled with the Canu assembler (Canu, RRID:SCR_015880; v1.8.0) [31] with the command "canu correctedErrorRate=0.105 corMhapSensitivity=normal corOutCoverage=100 -p Cooinda -d assembly genomesize=2.3g -pacbio-raw Cooinda_SMRT_ONT_combined.fasta. The resulting contigs were polished with two rounds of the Arrow pipeline, each consisting of aligning the raw SMRT reads to the assembly with pbmm2 (https://github.com/PacificBiosciences/pbmm2) and correcting the sequencing errors using gcpp [32].

The Arrow-polished SMRT/ONT assembly was scaffolded using Alpine dingo 10X linked-reads as in ARCS [107]. The 10X data was aligned using the linked-read analysis software provided by 10X Genomics, Long Ranger, v2.1.6 [108]. Misaligned reads and reads not mapping to contig ends were removed, and all possible connections between contigs were computed keeping best reciprocal connections. Finally, contig sequences were joined, spaced by 10kb with stretches of N's, and if required reverse complemented.

To further improve the assembly, another round of polishing was performed by aligning the Illumina short reads from the 10X Chromium sequencing to the assembly using minimap2 [109] (v2.16) and correcting the sequencing errors using Racon (Racon, RRID:SCR_017642; v1.3.3) [110].

The Hi-C data was processed using Juicer (Juicer, RRID:SCR_017226) [111], and used as input into the 3D-DNA pipeline [112] to produce a candidate chromosome-length genome assembly. We performed additional curation of the scaffolds using Juicebox Assembly Tools [113].

After scaffolding and correction, all raw SMRT and ONT reads were separately aligned to the assembly with Minimap2 (v2.16) (-ax map-pb/map-ont) [109]. The combined alignments were used by PBJelly (pbsuite v.15.8.24) [114] for one round of gap filling.

Following scaffolding, another round of polishing was done to further improve the assembly. Polishing was performed by aligning the Illumina short reads from the Chromium sequencing to the assembly using Long Ranger v2.2.2 and correcting the SNVs and indels using Pilon (Pilon, RRID:SCR_014731) [33].

The Pilon-polished genome underwent a final scaffold clean-up using Diploidocus as described in Edwards et al. [27] to generate a high-quality core assembly, remove low-coverage artefacts and haplotig sequences, and filter any remaining vector/adapter contamination. This reduced the final number of scaffolds to 632 (780 contigs), including the mtDNA.

Assembly completeness was evaluated using BUSCO v5.2.2 [37] short mode against the Carnivora_ob10 data set (n=14,502) implementing BLAST+ v2.11.0 [115], HMMer v3.3 [116], and Metaeuk v20200908 [117]. "Complete" BUSCO genes with available sequences were compiled across Alpine dingo Cooinda and nine canid genomes (Desert dingo [6], two Basenji's (China and Wags) [27], two German shepherd dogs (Nala and Mischa) [28, 36], Great Dane [38], Labrador [39], Dog10K Boxer [40], and Greenland Wolf [41]) using BUSCOMP v1.0.1. Additional kmer-based assembly completeness and quality evaluations were performed using Merqury v21.3 [42] from the 10x reads.

Chromosome mapping and variation

Chromosome mapping was completed in 2019 using the CanFam v3.1 reference genome downloaded from Ensembl (GCF_000002285.3 [118]). Full length chromosomes were renamed with a CANFAMCHR prefix and used for reference mapping. The final Cooinda

Alpine dingo genome assembly was mapped onto the CanFam3.1 reference genome using Minimap2 v2.16 [109] (-x asm5 --secondary=no --cs) to generate PAF output. Scaffolds were assigned to CanFam3.1 chromosomes using PAFScaff v0.2.0 [119] based on Minimap2-aligned assembly scaffold coverage against the reference chromosomes. Scaffolds were assigned to the chromosome with highest total coverage. Scaffolds failing to map onto a chromosome were rated as "Unplaced".

Comparison of Alpine and Desert dingo genomes

To investigate the variation between the dingo ecotypes we used Circos [43]. Circos uses a circular ideogram layout to facilitate the display of relationships between the genomes using ribbons, which encode the position and number of SNV's, small indels and large indels for each of the 38 autosomes and the X chromosome. SNV and indel numbers were calculated using MUMmer4 'show-snp' script following pairwise alignments [44] (v4.0.0 beta 2).

Synteny plot between the Alpine and published Desert dingo assembly [6] was conducted using GenomeSyn [47]. With GenomeSyn the position of the genome is indicated by a black horizontal ruler with tick marks. Syntenic blocks between the genomes are displayed as light grey regions with white illustrating non-syntenic regions. Inversions are represented by red-brown curves.

We used GeMoMa v1.6.2beta [48] to further investigate whole chromosomal events. Here we mapped genes onto the Alpine Dingo assembly following previously described protocols [28]. Subsequently, we checked the synteny of the genes in the reference genome and the target genome using the module GeMoMa module SynthenyChecker. This module uses the GeMoMa annotation with information for reference gene and alternative to determine the best homolog of each transcript. Comparing the order of genes in the reference and the target genome, it allows to determine breakpoints of chromosomal events.

Phylogenetic analyses

All 39 full-length chromosomes in the final assembly were aligned to the corresponding chromosomes in nine published canine *de novo* genome assemblies (Desert dingo [6], two basenjis (China and Wags) [27], two German shepherd dogs (Nala and Mischa) [28, 36], Great Dane [38], Labrador [39], Dog10K Boxer [40], and Greenland Wolf [41]) using MUMmer4 [44]. SNVs and small indels (deletions and insertions <50bp) were called using MUMmer4 call-SNPs module for all possible pairings (Supplementary Table 2). Copy number (CNV) and SVs were also called using svmu (v0.2) [120] however these were not included in the phylogeny. SNV's and indels were analyzed separately. Distance matrices were generated from the inter-canid differences in SNV's and indels and then transformed to WA distance [49]. Glazko et al. [49] report WA has better phylogenetic properties against normalization of genome sizes than other coefficients.

Phylogenetic analyses using maximum parsimony were generated from the R-package 'phangorn' version 2.8.1 [121]. The analyses were run as unrooted networks to test the hypothesis that the wolf was the outgroup. To test the stability of the nodes, a Bayesian bootstrap was applied to the original distance matrix using the program bayesian_bootstrap (github.com/lmc2179/bayesian_bootstrap) and the phylogenetic analysis was re-calculated. This process was iterated 500,000 times. The consensus phylogenetic trees were rooted on the branch leading to wolf, the values indicate the percentage of times that a node occurred. The Y-axis and branch lengths were rescaled to the original number of differences in SNV's and indels among the taxa. The retention index that measures the fit of the network to the distance matrix exceeded 94% for all 500,000 trees of SNVs and indels.

Non-metric multidimensional scaling (NMDS) was calculated from the distance matrices and scores for the taxa calculated from the largest two axes. Minimum spanning trees were

calculated among the scores in NMDS space. NMDS and minimum spanning trees were calculated in Past 4.04 [122].

Mitochondrial genome

Genome assembly workflow

A 46,192 bp contig from the assembly mapped onto the CanFam reference mtDNA (NC_002008.4), constituting a repeat of approx. 2.76 copies of the mtDNA. The CanFam mtDNA was mapped onto this contig using GABLAM v2.30 [123] and full-length mtDNA copy with highest similarity to CanFam mtDNA was extracted along with 8 kb each side. PacBio reads were mapped onto this mtDNA contig using minimap2 v2.22 [109] and 10x linked reads mapped using BWA v0.7.17 [124] for polishing with HyPo v1.0.3 [125] (32.7 kb assembly size at 673X coverage). The CanFam mtDNA was re-mapped onto the polished assembly using GABLAM v2.30.5 [123] and a 16,719 bp sequence extracted, starting at position 1 of the CanFam sequence. The mtDNA was annotated with the MITOS2 server [126] for submission to NCBI GenBank (accession: OP476512).

Comparison of dingo mtDNA genomes

The mtDNA genome of Alpine dingo Cooinda was compared with the Desert dingo [6]. Direct observation of the D-loop region in the two dingoes suggested there was a 10bp repeat and the canids differed in the number of repeats. Imperfect tandem repeats have previously been reported in canids [50]. The D-loop region in Alpine dingo Cooinda was folded using the program mfold [52] to determine ay underlying structures.

To test whether the mtDNA from dingo Cooinda fell within the previously described SE clade we compared the assembly with 33 other canids, including dogs from New Guinea and Taiwan [6, 22, 54, 55]. In this case multiple large gaps were in some of the ancient samples,

so the initial assembly was modified based on the predicted secondary structure folding. A inter neighbor-joining network analysis with $\alpha = 0.5$ was completed in POPART [53]. A limitation of this analyses is that large sections of multiple mtDNA's were unknown, so it was not possible to distinguish deletions from missing data. Understanding these differences may be biologically important, particularly if the predicted folding of the D-loop region is biologically significant.

DNA methylome

MethylC-seq library preparation

Genomic DNA was extracted from whole blood using DNeasy Blood & Tissue kit (Qiagen, USA). MethylC-seq library preparation was performed as described previously [127]. Briefly, 1 ug of genomic DNA was sonicated to an average size of 300 bp using a Covaris sonicator. Sonicated DNA was then purified, end-repaired and 3'-adenylated followed by the ligation of methylated Illumina TruSeq sequencing adapters. Library amplification was performed with KAPA HiFi HotStart Uracil+ DNA polymerase (Millenium Science Pty Ltd).

MethylC-seq data analysis

The methylome library was sequenced on the Illumina HiSeq X platform (150 bp, PE), generating 377M reads. Sequenced reads in fastq format were trimmed using the Trimmomatic software (ILLUMINACLIP:adapter.fa:2:30:10 SLIDINGWINDOW:5:20 LEADING:3 TRAILING:3 MINLEN:50). Trimmed reads were mapped (GCA_012295265.2_UNSW_AlpineDingo_1.0_genomic.fna genome reference, containing the lambda genome as chrLambda) using WALT with the following settings: -m 10 -t 24 -N 10000000 -L 2000. Mapped reads in SAM format were converted to BAM format; BAM files were sorted and indexed using SAMtools. Duplicate reads were removed using Picard Tools v2.3.0. Genotype and methylation bias correction were performed using MethylDackel

(MethylDackel extract dingo_lambda.fasta $input_bam -o $output --mergeContext --minOppositeDepth 5 --maxVariantFrac 0.5 --OT 10,140,10,140 --OB 10,140,10,140). The numbers of methylated and unmethylated calls at each genomic CpG position were determined using MethylDackel (MethylDackel extract dingo_lambda.fasta $input_bam -o output –mergeContext). Segmentation of hypomethylated regions into CpG-rich unmethylated regions (UMRs) and CpG-poor low-methylated regions (LMRs) was performed using MethylSeekR (segmentUMRsLMRs(m=meth, meth.cutoff=0.5, nCpG.cutoff=5, PMDs = NA, num.cores=num.cores, myGenomeSeq=build, seqLengths=seqlengths(build), nCpG.smoothing = 3, minCover = 5).

Cooinda UMR coordinates were converted to the Desert dingo genome assembly using LiftOver following genomewiki.ucsc.edu pipeline (http://genomewiki.ucsc.edu/index.php?title=Minimal_Steps_For_LiftOver). Briefly, the query (Desert dingo) genome build was split into individual scaffolds using *faSplit* (i). The we performed pairwise sequence alignment of query sequences from (i) against the Cooinda genome build using BLAT, Then, coordinates of .psl files were changed to parent coordinate system using *liftUp* and alignments were chained together using axtChain. Chain files were combined and sorted using *chainMergeSort*; alignment nets were made using *chainNet*. Finally, liftOver chain file was created using *netChainSubset*. Cooinda UMRs in .bed format were lifted over to Desert dingo genome assembly using created liftOver chain file. Average methylation was calculated for Cooinda UMRs and compared to that of corresponding lifted-over regions in the Desert dingo genome. Cooinda UMRs with >50% methylation increase in Desert dingo genome were considered as hypermethylated in the Desert dingo.

Morphology

Skull Morphometrics

To examine cranial morphology, we obtained a 3D model of Cooinda's cranium using an Artis Pheno Computed Tomography (CT) Scanner. The skull was damaged slightly when the brain was extracted, so the damaged region (dorsal part of the calvarium) was reconstructed using Blender to reassemble the separated fragment following guidelines for digital specimen reconstruction outlined by Lautenschlager [128] (Supplementary Fig. 10A). Geometric morphometric landmarks (n=45) were collected on the 3D cranial model using Stratovan Checkpoint (Stratovan Corporation, Davis, CA version 2018.08.07) and analyzed with MorphoJ [129], following the landmarking protocol used for dingo crania by Koungoulos [65]. This approach uses 45 landmarks along the left side of the cranium, covering all major anatomical features and regions, excepting a few fragile processes which are frequently lost in prepared specimens (Supplementary Fig. 11; Supplementary Table 4**)**. The cranial landmarks collected on the Cooinda cranium were incorporated into an existing data set comprising 91 Alpine dingoes and 101 Desert dingoes [65] and subject to Procrustes superimposition to remove all non-shape differences, due to translation, rotation and scaling [130]. The resultant Procrustes shape variables were ordinated using Principal Component Analysis (PCA) to assess the cranial morphology of Cooinda in relation to other dingoes. To assess the impact of allometry on cranial shape variation in the sample, a regression of Procrustes shape variables against log centroid size was performed using MorphoJ [129]. Residuals were extracted from this regression and ordinated using PCA (see Supplementary Material).

Brain imaging

Cooinda's brain and that of a domestic dog (Kelpie) of the same body size were extracted. Brains of these animals, which died within 2 weeks of each other, were fixed in Sigma-

Aldrich 10% Neutral Buffered Formalin (NBF) after extraction and were washed with Gd DTPA (gadolinium-diethylenetriamine pentaacetic acid) solution prior to imaging. Brains were scanned using high-resolution magnetic resonance imaging (MRI). A Bruker Biospec 94/20 9.4T high field pre-clinical MRI system was used to acquire MRI data of a fixed dingo and domestic dog brain. The system was equipped with microimaging gradients with a maximum gradient strength of 660mT/m and a 72mm Quadrature volume coil. Images were acquired in transverse and coronal orientation using optimized 2D and 3D Fast Spin Echo (FSE) and Gradient Echo (MGE) methods. Image resolution was 200x200x500 and 300x300 microns isotropic for type 3D and 2D pulse sequences, respectively. To quantify brain size, we used the open-source software 3D Slicer “Segment Statistics” module [66]. The software considers the pixel spacing and slice thickness set to calculate the volume accurately. The threshold was empirically set to the grayscale intensity 1495, where everything below that is background, and ventricles and everything above that is the brain.

Acknowledgements

Comments from four reviewers improved the manuscript. We would like to thank Luci Ellem, and Dingo Sanctuary Bargo for providing frequent access to Cooinda. Picture of Cooinda was taken by Luci Ellem. Staff at the Vineyard Veterinary Hospital provided constant encouragement. Mike Archer suggested the usage of the term “archetype” and we thank him for valuable taxonomic discussions. Richard Melvin conformed the purity of Cooinda using microsatellites. We thank Shyam Gopalakrishnan and Simon Ho for discussions and Hauke Koch for assistance with translation. SMRT sequencing was conducted at the Ramaciotti Center for Comparative Genomics at University of New South Wales (UNSW). The ONT, 10X Chromium and Bionano genomics data were collected within the Kinghorn Centre for Clinical Genomics at the Garvan Institute of Medical Research, Sydney, Australia and the Hi-C data at Baylor College of Medicine. The high field

pre-clinical MRI system was located at the Biological Resources imaging Laboratory at UNSW. Thanks to Jiaming Song for the GenomeSyn analyses, Mihwa Lee for help with DNA folding and Tim Smith for synteny plots. Bootstrapping was on the Wesleyan computing cluster. Thanks go to the facilities of Sydney Imaging at the University of Sydney, and the expertise of Pranish Kolakshyapati in generating the Artis Pheno CT scans of Cooinda's cranium. Finally, we thank Sandy Ingelby and Harry Parnaby of the Australian Museum for their assistance in facilitating scans of Cooinda's cranium.

Availability of supporting data and materials

The chromosomal assembly is available at GCA_012295265.2. The mtDNA and has been submitted to NCBI GenBank (accession: OP476512). The methylation data is available at https://www.ncbi.nlm.nih.gov/geo/query/acc.cgi?acc=GSE212509. The 3D Cranial landmark data are available on Figshare at https://figshare.com/articles/dataset/Cooinda_Alpine_Dingo_3D_Cranial_Landmarks/2052304. The raw Dicom data for the magnetic resonance imaging (MRI) of the Alpine dingo and domestic dog brain are available on Figshare at https://figshare.com/articles/dataset/Dicom_data_MRI_Alpine_dingo_and_domestic_dog_brain/20514693.

Additional Files

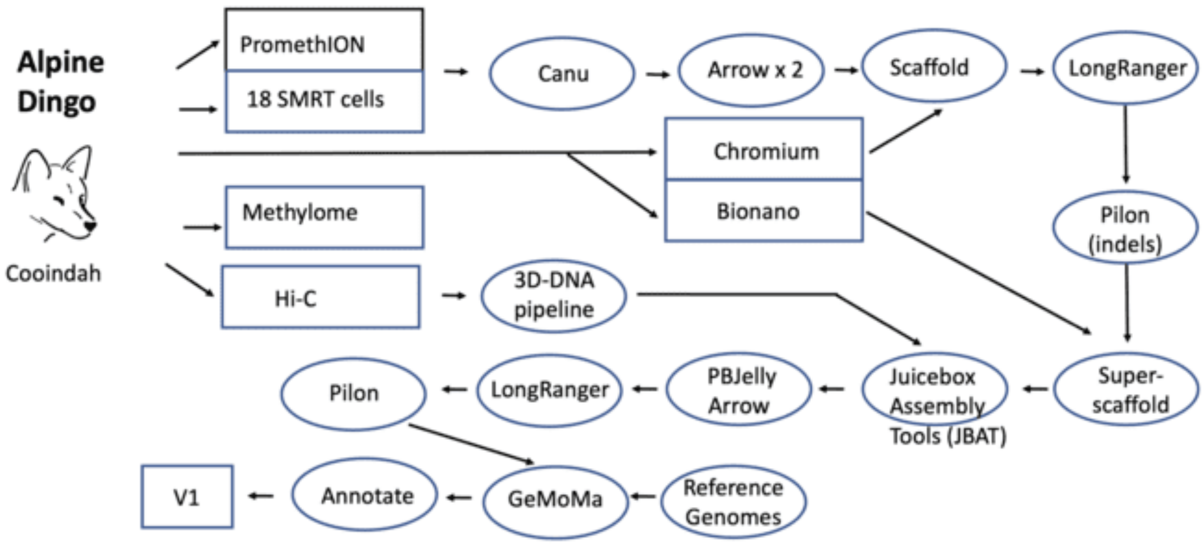

Supplementary Figure 1 Title. Schematic overview of project workflow

Supplementary Figure 1 Legend. Alpine Dingo Cooinda DNA was derived from blood of a single female from the Dingo Sanctuary Bargo. Sequences were generated on the Pacific Biosciences Sequel instrument (V2 chemistry) and Oxford Nanopore PromethION instrument (guppy bascaller Version 3.0.6+9999d81) to ~30x genome coverage, each, based on a genome size estimate of 2.4 Gb (this estimate is used for all coverage estimates). All long read sequences were assembled with the Canu v1.8 algorithm then error corrected twice using the Arrow genomic consensus polishing module. The assembly was scaffolded with Chromium 10x linked-reads (~41x coverage excluding the barcode) using Long Ranger v2.1.6 using DNA from the same animal. Polishing of the assembly for residual indels was done by aligning the Illumina data with Minimap2 and the Racon algorithm. Single molecule Bionano data (~57x effective coverage) was then used to superscaffold the sequence assembly using DNA extracted from the same canid. For this, single molecule optical maps were first de novo assembled into consensus maps, which were than aligned to the sequence assembly in silico digested with the same labelling enzyme for hybrid scaffolding, using Bionano Solve (v3.2.2_08022018) with RefAligner (7782.7865rel). This assembly was

further scaffolded to chromosome-length by DNA Zoo (www.dnazoo.org/methods). Briefly, an *in situ* Hi-C library was prepared from the same individual and sequenced to 29x coverage. The Hi-C data was processed using Juicer [111], and used as input into the 3D-DNA pipeline [112] to produce a candidate chromosome-length genome assembly. We performed additional finishing on the scaffolds using Juicebox Assembly Tools [113]. The assembly was then long-read gap filled with the PBJelly algorithm, and the additional data error corrected using Arrow [32]. The Chromium data was mapped onto the assembly with the Long Ranger v2.1.6 program and the final assembly was then polished using the Pilon algorithm. Of the 2.4 Gb assembled genome, the total assembly N50 contig and scaffold lengths are 23.1 Mb and 64.8 Mb, respectively. The assembled contigs were then aligned to CanFam3.1 for chromosome assignments. Regulatory landscape was characterised by whole genome bisulphite sequencing.

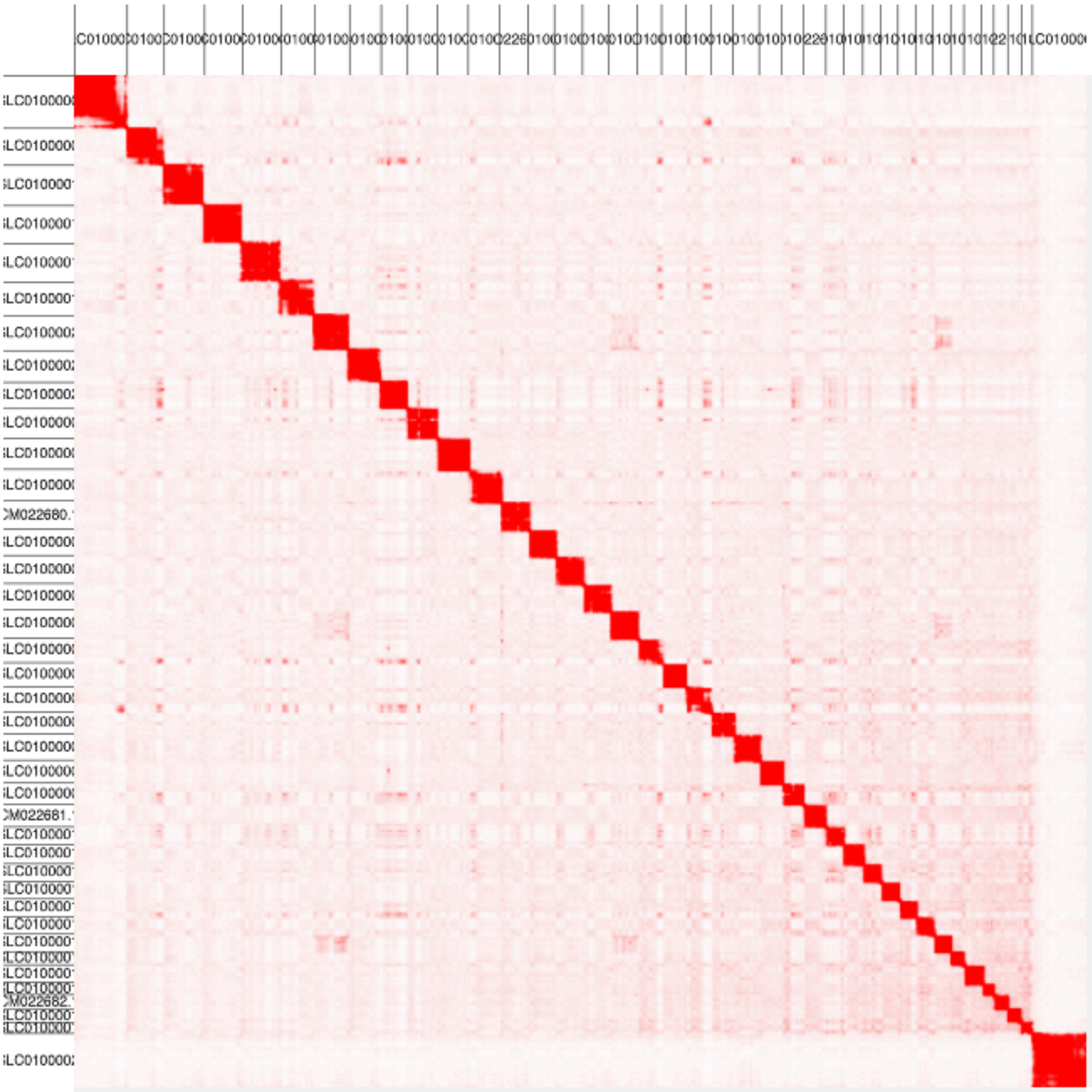

Supplementary Figure 2 Title: Alpine dingo assembly after Hi-C correction

Supplementary Figure 2 Legend: Contact matrices (visualised in Juicebox.js) after the chromosome-length Hi-C upgrade. The chromosome-length contact map can be viewed at multiple resolutions using Juicebox.js [34] following the link https://tinyurl.com/ycbkezf4.

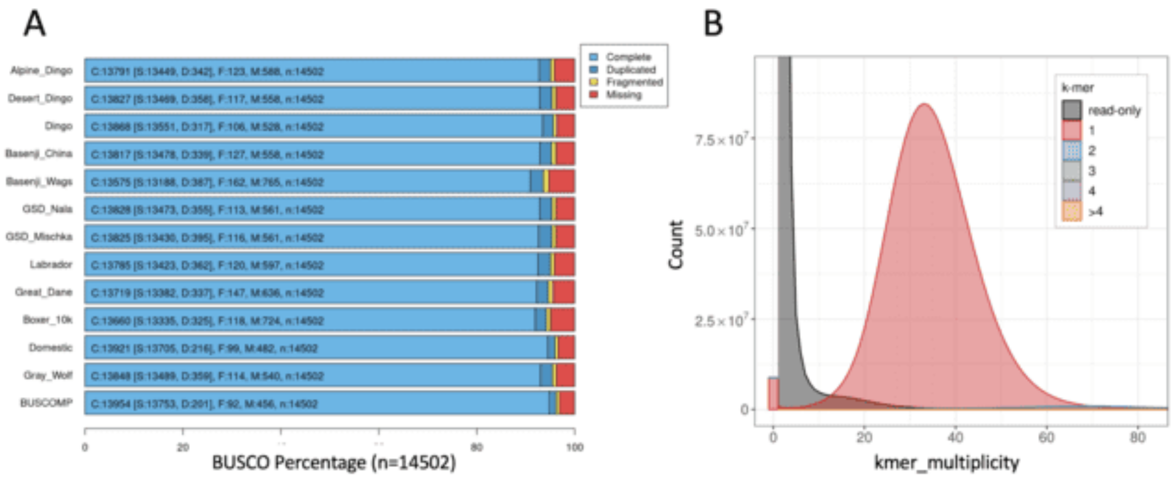

Supplementary Figure 3 Title: Assembly statistics

Supplementary Figure 3 Legend: (A) BUSCO ratings for Cooinda assembly, compared to CanFam4. Purple, original assembly; Black, scaffolding/polishing steps; Blue, final assembly; Red, CanFam4. Dashed red lines mark CanFam4 statistics.

(B) 10x read kmers frequency distributions for kmers with different assembly copy numbers derived from A Read 1 (16bp barcodes trimmed) and B Read 2 (barcodes not trimmed).

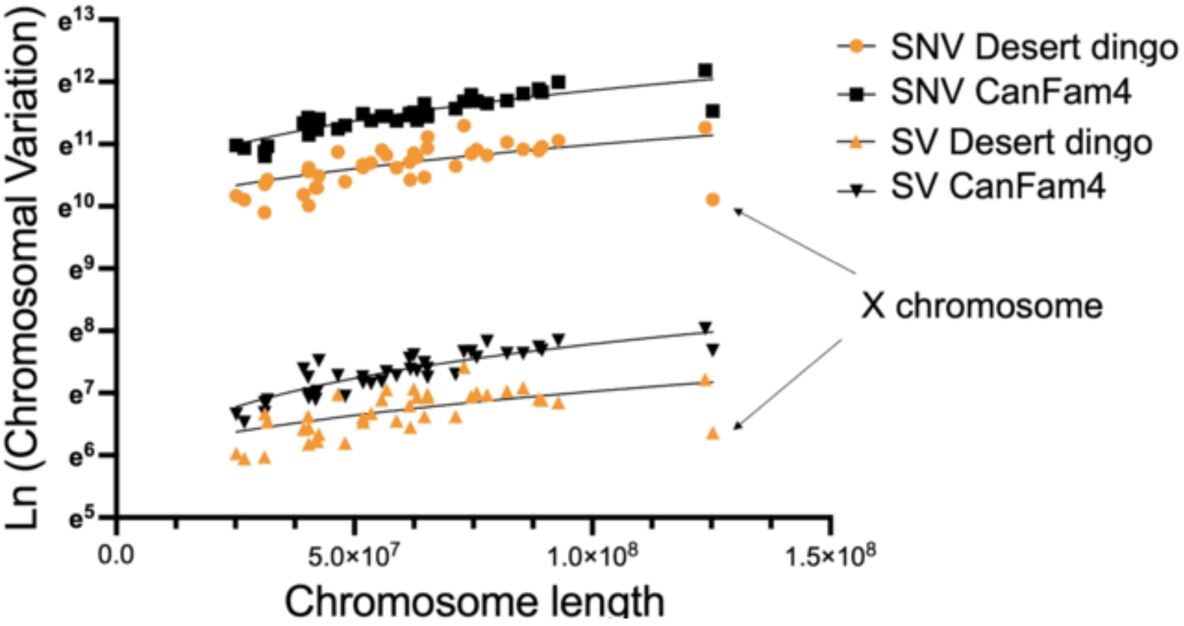

Supplementary Figure 4: Genome shows a deficiency of variation on the X chromosome

Supplementary Figure 4 Legend: SNV (single nucleotide variant) and SV (structural variation) comparisons show a relative deficiency of variation on the X chromosome. Line represents a regression through the non-transformed data and each point represents one chromosome with the length of the Alpine dingo and SNV's or SV relative to the Desert dingo genome or CanFam4. Y=3.8e-4x+21305, 1.1e-4+31753, 7.2e-5+406.7, 2.5e-5+363.1 with an r^2 of 0.37, 0.74, 0.33, 0.77 for SNV Desert dingo, SNV CanFam, SV Desert dingo and SV CanFam, respectively. If the SNV and SV Desert dingo X chromosome data are excluded the r^2 of these regressions increases to 0.67 and 0.54, respectively.

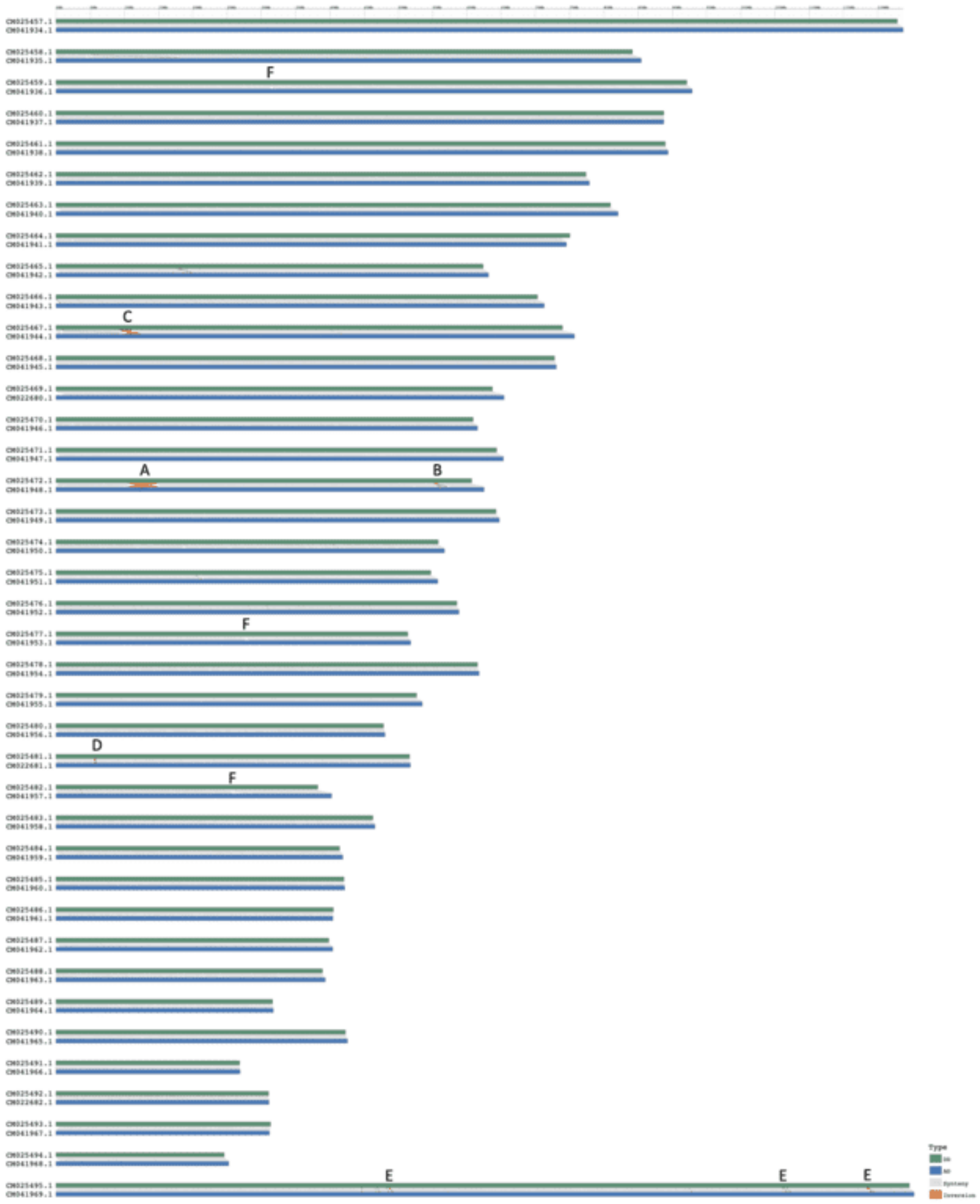

Supplementary Figure 5: Synteny analyses

Supplementary Figure 5 Legend: Synteny plot of Alpine dingo Cooinda (AD) in blue against Desert dingo Sandy in orange (DD). A. Shows the 3.45Mb rearrangement on Chromosome 16. B. Shows the complex rearrangement between 55-57 Mb downstream on Chromosome 16. C Smaller inversion on Chromosome 11. D. Small inversion on

Chromosome 25. E. Multiple possible small inversions on X chromosome. Other smaller rearrangements are possible. F. Possible duplication like events.

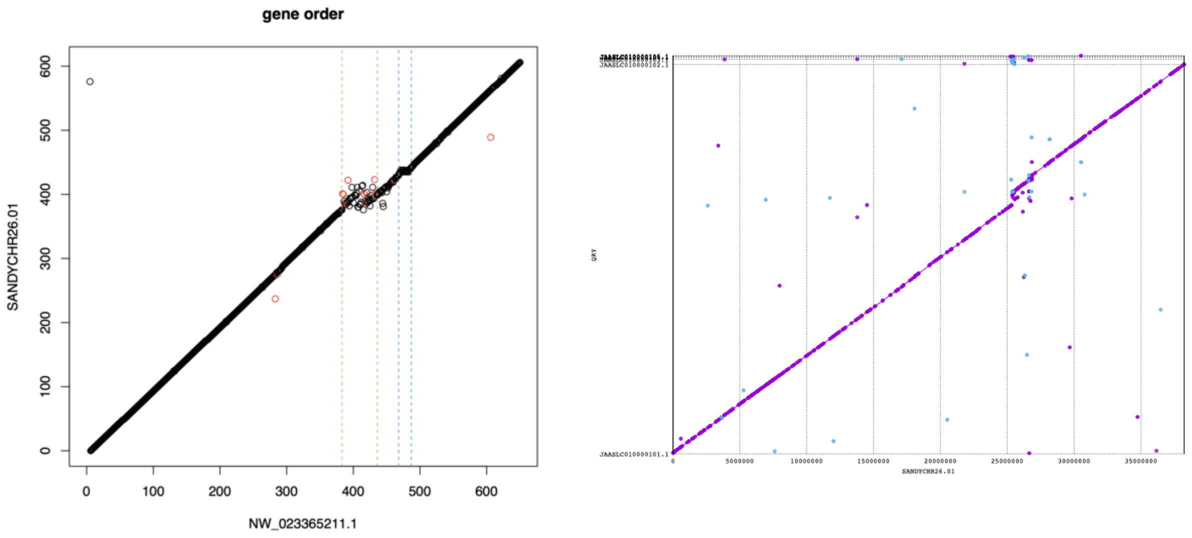

Supplementary Figure 6: Gene order plot comparing Chromosome 26 for Cooinda the Alpine Dingo (X-axis) and Desert Dingo Sandy (Y-axis) using GenomeSym (left) and MUMmer (right).

Supplementary Figure 6 Legend: In GeMoMa plot (left) the green and the blue dashed lines indicate the two structural events on chromosome 26 of Cooinda. The same region is shown using MUMmer (right).

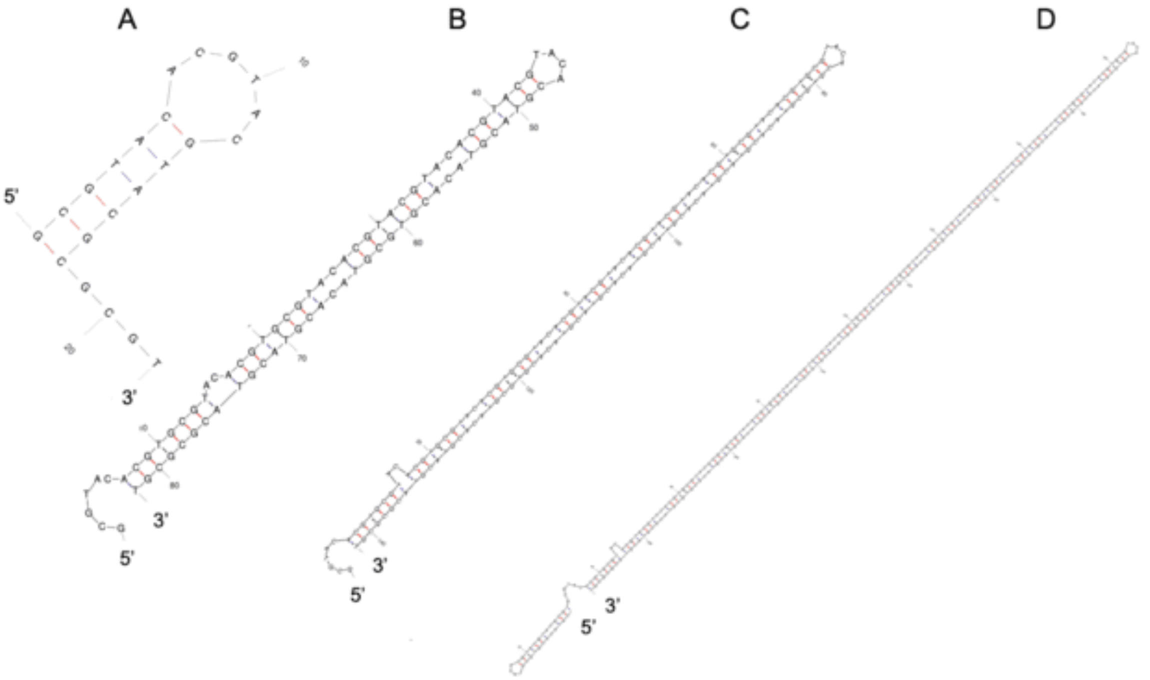

Supplementary Figure 7: Possible folding of 10bp repeats in D-loop region

S Supplementary Figure 7 Legend: (A) 1 repeat, ΔG=-4.68, (B) 7 repeats ΔG=-29.07, (C) 13 repeats ΔG=-48.21, (D) 28 repeats ΔG=-97.71.

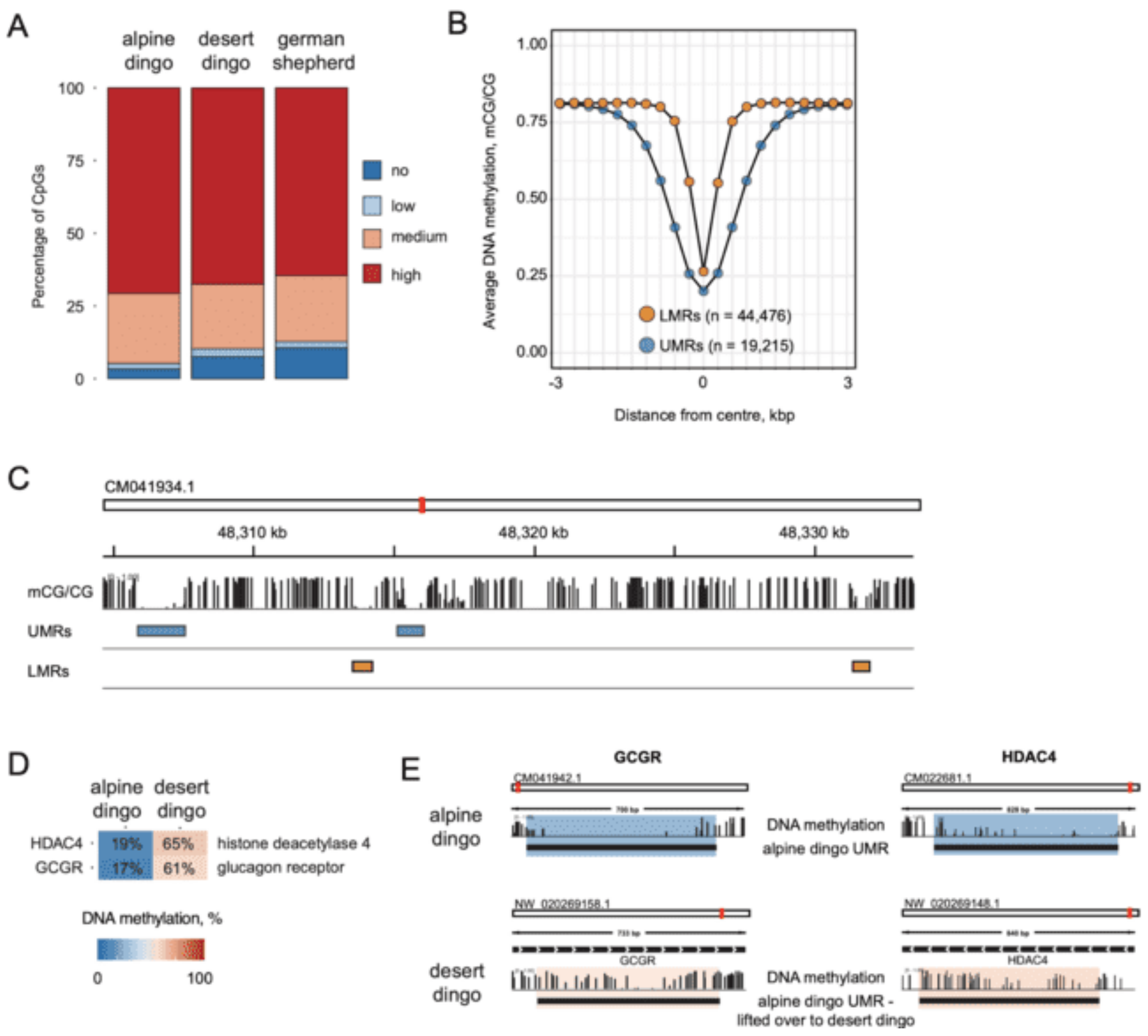

Supplementary Figure 8: DNA methylation profiling of alpine dingo Cooinda's whole blood

Supplementary Figure 8 Legend: **(A)** Percentage of CpG sites with different levels of methylation. High, 80-100%; medium, 20-80%; low, >0-20%; no, 0%. **(B)** Average DNA methylation profiles of hypomethylated regions into CpG-rich unmethylated regions (UMRs) and CpG-poor low-methylated regions (LMRs). **(C)** Integrative Genomics Viewer **(**IGV) browser track depicting DNA methylation profile and putative regulatory elements (UMRs and LMRs). **(D)** Heatmap depicting average DNA methylation at hypomethylated UMRs in the alpine dingo genome, which are more than 50% methylated in the desert dingo genome. **(E)** IGV browser track depicting hypomethylated UMRs within GCGR and HDAC4 genes in the alpine dingo genome, which are hypermethylated in the desert dingo genome.

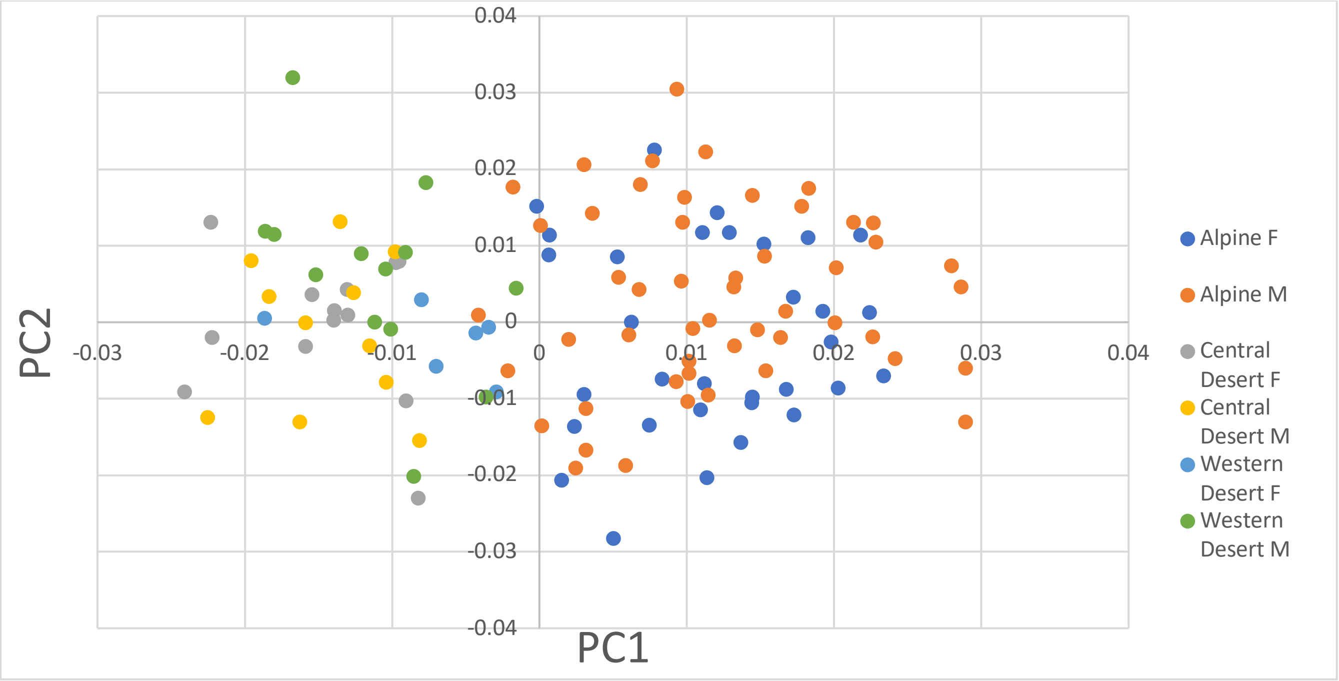

Supplementary Figure 9

Supplementary Figure 9 Legend: Scatterplot of PC1 and PC2 values for sexed dingo specimens. The distribution of greater PC2 values slightly favors males in all populations except for Central Desert, which is a very gracile population with relatively minimal differences between the cranial morphology of different sexes. In general, however, the difference in PC2 between males and females in any population is very marginal and neither greater nor lesser values are particularly strongly associated with either sex.

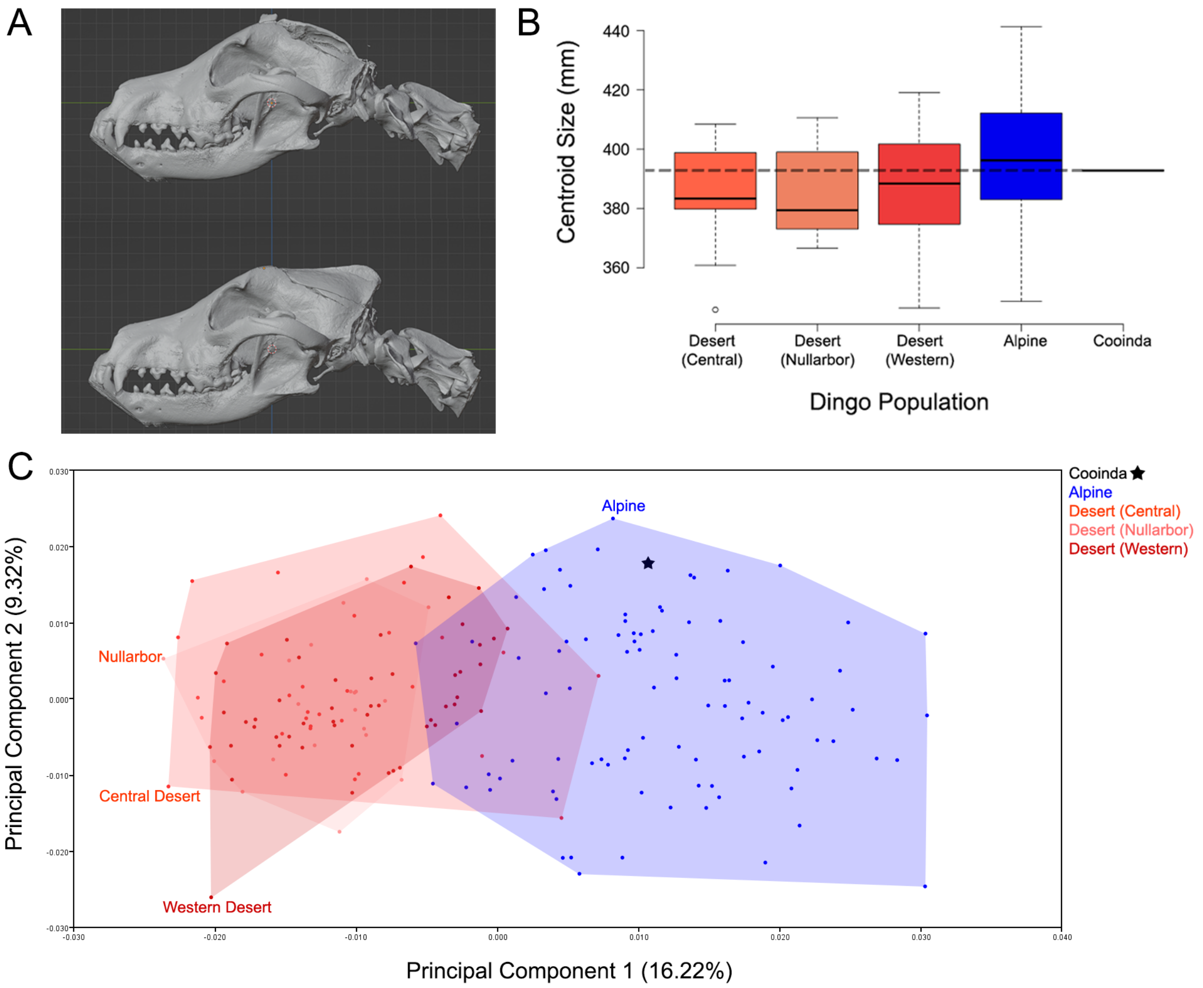

Supplementary Figure 10: Cooinda's cranial morphology

Supplementary Figure 10 Legend: (A) Cranium before (upper) and after (lower) cranial reconstruction (lower). This was required because the brain was removed immediately after death, which caused some damage to the braincase. (B) Cranium size. Cooinda's cranium is larger than the median size reported for Desert dingoes, in line with Alpine dingoes in general, although this difference is not major and there is heavy overlap between the two regions. Her centroid size (392.80mm) is slightly below the pooled Alpine mean (396.49mm) and median (396.23mm), but well below the mean (403.26mm) and median (403.64mm) for Alpine males specifically, which make up a majority of the sample (male *n* = 50; female *n* = 33; sex unknown *n* = 9). Alpine dingoes, as with all regional dingo populations, exhibit significant sexual dimorphism in centroid size with males being on average 4.20% larger

[65]. (C) Principal component ordination of allometric residuals. The residuals of a regression of shape against log centroid size were plotted to further explore the role of size (allometry) in overall form. This revealed that the separation of Alpine and Desert populations, and Cooinda's position within the former, remains essentially identical to their original distribution s (Fig. 5a) when the size-related allometric component of form is removed from consideration.

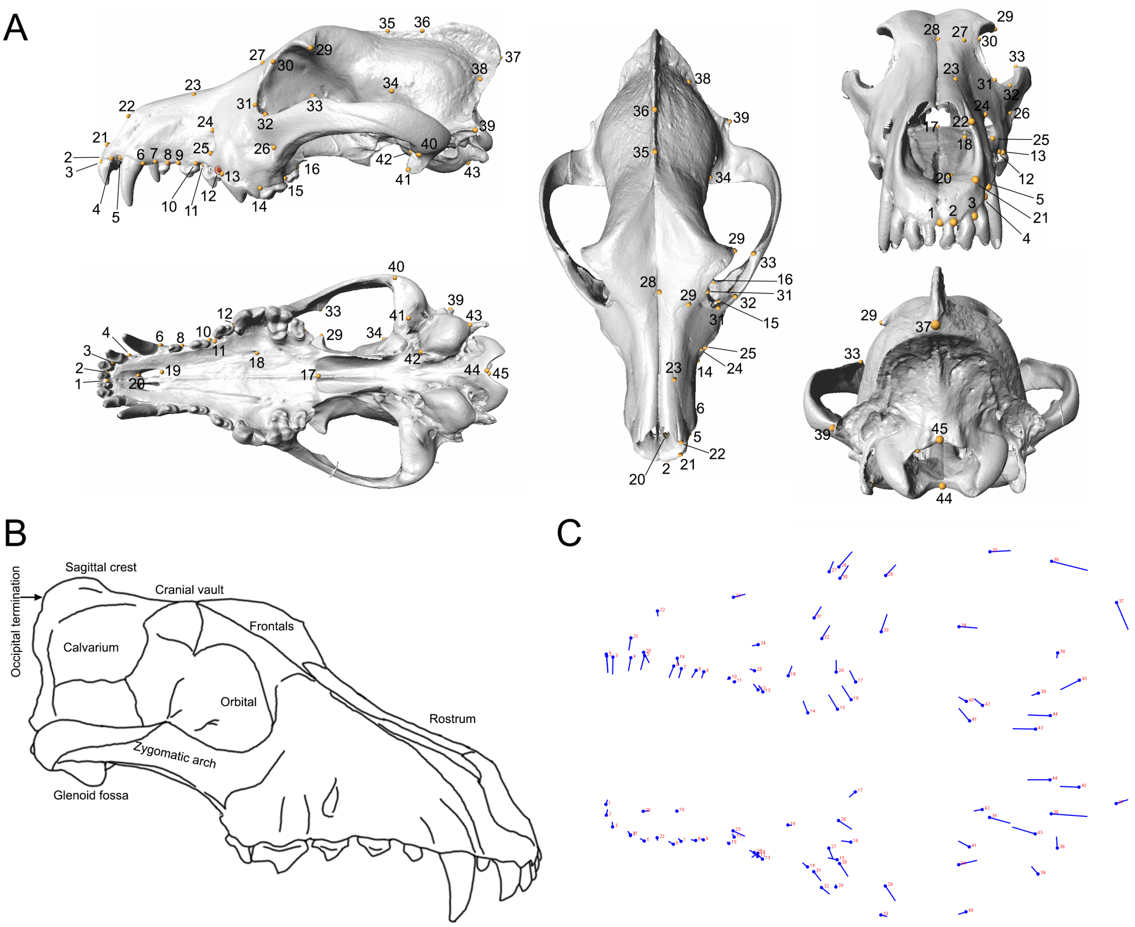

Supplementary Figure 11

Supplementary Figure 11 Legend: (A) Landmarks used in this study. (B) diagram of canid skull with basic anatomical features and regions referred to in-text. (C) Lollipop figures illustrating change in landmark positions along PC2 in lateral (upper) and dorsal (lower) views. The lollipop "head" represents the mean position, and the end of the "stick" represents its position with the highest PC1 score.

Supplementary Table 1. Alpine dingo SNVs and SVs summary by chromosome.

Chromosome	Alpine dingo SNV count vs		Alpine dingo SV count vs		Chromosome bp
	Desert dingo	CanFam4.0	Desert dingo	CanFam4.0	
**1**	77926	195865	1370	3086	123706252
**2**	55068	134823	1192	2074	85446638
**3**	63384	162076	939	2552	92885137
**4**	53989	145400	1001	2287	88750986
**5**	57509	137691	981	2181	89373516
**6**	49987	114964	1067	2510	77892171
**7**	61659	120334	1132	2074	82078758
**8**	51280	132310	1038	2138	74537974
**9**	48343	87955	1027	1566	63158580
**10**	42060	106101	754	1477	71288900
**11**	54954	119566	1100	1950	75713417
**12**	80643	119102	1661	2123	73055177
**13**	67246	96899	1076	1411	65408697
**14**	44691	97750	899	1913	61550799
**15**	56205	93452	1023	1627	65319253
**16**	51884	99759	1176	2021	62499104
**17**	35216	114685	754	1783	64752584
**18**	50255	93879	1160	1536	56713732
**19**	54419	94065	988	1311	55746904
**20**	40988	86977	703	1446	58849343
**21**	41147	96550	691	1293	51792689
**22**	33737	94950	636	1604	61776113
**23**	44240	87440	794	1274	53490029
**24**	32824	81112	491	1037	48051091
**25**	43100	97505	739	1413	51754766
**26**	38604	91768	757	1406	40258947
**27**	52772	76375	1075	1447	46564242
**28**	29616	81846	517	984	41881905
**29**	29642	75460	507	1121	42159209
**30**	22298	69332	486	1074	40431870
**31**	41002	82966	628	1015	40406310

**32**	26587	83791	614	1610	39322259
**33**	33909	57901	692	977	31731374
**34**	35988	89665	567	1843	42557588
**35**	24494	56238	383	683	26863133
**36**	19944	53165	393	796	31103624
**37**	31481	49270	789	935	31168689
**38**	26115	58654	416	780	25215458
**X**	24584	102071	582	2166	125292608

Supplementary Table 2. Distance matrix table showing SNVs above diagonal and Indels below. All possible pairwise alignments were generated using MUMmer4 [44] (v4.0.0 beta 2) and SNVs/indels numbers calculated using MUMmer4 'show-snp' script.

	Desert	Alpine	Basenji1 (China)	Basenji2 (Wags)	GSD1 (Nala)	GSD2 (Mischa)	Labrador	Boxer	Great Dane	Greenland Wolf
**Desert**	-	1934204	4379273	4058304	4157347	4099899	4266975	3956320	3858069	5039138
**Alpine**	3525802	-	4351866	4048746	4125867	4061800	4219881	3939675	3802100	4696525
**Basenji1**	6813866	6946862	-	2199194	3893739	3855744	3922731	3700555	3605316	5155027
**Basenji2**	6482039	6567647	4372616	-	3515180	3471928	3686649	3375257	3246911	4949577
**GSD1**	6290364	6362924	6237235	5742582	-	2064119	3477794	3101130	3007546	5078984
**GSD2**	6229282	6301226	6186934	5663021	3396958	-	3426212	3072348	2990169	5049776
**Labrador**	7072684	7122926	6758598	6529100	6029553	5975640	-	3174798	3162230	5252186
**Boxer**	6124985	6229361	6004983	5678411	5061694	5025968	5816010	-	2773431	4947075
**Great Dane**	6601081	6693070	6455860	6160610	5582788	5578574	6440463	5255792	-	4776954
**Greenland Wolf**	7273469	6925872	7606227	7343748	7339762	7266186	8130863	7190906	7642878	-

GSD is German shepherd dog

Supplementary Table 3. Mean and median PC2 scores for different sexes from dingo populations

.

	PC2 Mean	PC2 Median
Alpine F	-0.00203	-0.00481572
Alpine M	0.003143	0.001463463
Central Desert F	-0.00062	0.000931911
Central Desert M	-0.00131	-3.97883E-05
Western Desert F	-0.00226	-0.001064826
Western Desert M	0.00602	0.006917365

Supplementary Table 4. List and description of cranial landmarks used in this study. After Koungoulos [65].

Landmark #	*Description*
1	*Between left and right first incisors.*
2	*Between first and second incisor.*
3	*Between second and third incisor.*
4	*Posterior corner of third incisor alveolus.*
5	*Anterior corner of canine alveolus.*
6	*Posterior corner of canine alveolus.*
7	*Anterior corner of first premolar alveolus.*
8	*Posterior corner of first premolar alveolus.*
9	*Anterior corner of second premolar alveolus.*
10	*Posterior corner of second premolar alveolus.*
11	*Anterior corner of third premolar alveolus.*
12	*Posterior corner of third premolar alveolus.*
13	*Anterior corner of fourth premolar (carnassial) alveolus.*
14	*Posterior corner of fourth premolar (carnassial)/Anterior corner of first molar alveoli.*
15	*Posterior corner of first molar (carnassial)/Anterior corner of second molar alveoli.*
16	*Posterior edge of second molar alveolus.*
17	*Staphylion at edge of palate and choanal region.*
18	*Greater palatal foramen.*
19	*Proximal end of palatine fissure.*
20	*Distal end of palatine fissure.*
21	*Lower corner of distal incisive bone.*
22	*Upper corner of distal incisive bone.*
23	*Intersection of incisive, maxilla and nasal bones.*
24	*Upper end of infraorbital foramen ridge.*
25	*Lower end of infraorbital foramen ridge.*
26	*Lower limit of intersection of maxilla and zygomatic bones.*
27	*Upper limit of intersection of maxilla and frontal bones.*
28	*Upper limit of intersection of nasal and frontal bone.*
29	*Furthest extent of zygomatic process of frontal bone.*
30	*First "corner" of orbital rim.*
31	*Second "corner" of orbital rim.*
32	*Third "corner" of orbital rim.*
33	*Fourth "corner" of orbital rim.*
34	*Intersection of frontal, parietal and temporal bones.*
35	*Intersection of cranial midline with frontal-temporal boundary suture (bregma).*
36	*Intersection of the parietal and occipital bones on the sagittal crest.*
37	*Most proximal extent/tip of occipital protuberance (occiput).*
38	*Intersection of nuchal crest with parietal-temporal boundary suture.*
39	*Lower flare of nuchal crest.*
40	*Underside of mandibular fossa, where zygomatic process begins.*

41	*Underside of retroarticular process.*
42	*Anterior corner of auditory bulla.*
43	*Posterior corner of auditory bulla.*
44	*Lower central rim of foramen magnum, between occipital condyles.*
45	*Upper central rim of foramen magnum, between occipital condyles.*

Abbreviations

BLAST: Basic Local Alignment Search Tool**; BMG:** Bionano Genomics**; bp:** base pairs**; BUSCO:** Benchmarking Universal Single-Copy Orthologs**; CHD:** Canine hip dysplasia**; d.p.:** decimal point**; CNV:** Copy number variant**; gDNA:** genomic DNA**; GSD:** German Shepherd Dog**; HMM:** hidden Markov model**; HME:** High Molecular Weight**; ONT:** Oxford Nanopore Technologies**; ORF:** open reading frame**; PacBio:** Pacific Biosciences**; PCR:** polymerase chain reaction**; qPCR:** quantitative polymerase chain reaction**; RNA-seq:** RNA sequencing**; s.f.:** significant figure**; SMRT:** single-molecule real time; **SNV:** single-nucleotide variant; **SV**; structural variant

Ethics approval and consent to participate

All experimentation was performed under the approval of the University of New South Wales Ethics Committee (ACEC ID: 16/77B).

Competing interests

The authors declare that they have no competing interests.

Funding

This work was supported by an Australian Research Council Discovery award to J.W.O.B. (DP150102038). M.A.F. is funded by NHMRC APP5121190. M.A.F. is supported by a National Health and Medical Research Council fellowship (APP5121190). L.A.B.W. is supported by an Australian Research Council Future Fellowship (FT200100822). E.L.A. was supported by the Welch Foundation (Q-1866), a McNair Medical Institute Scholar Award, an NIH Encyclopedia of DNA Elements Mapping Center Award (UM1HG009375), a US-Israel Binational Science Foundation Award (2019276), the Behavioral Plasticity Research Institute

(NSF DBI-2021795), NSF Physics Frontiers Center Award (NSF PHY-2019745), and an NIH CEGS (RM1HG011016-01A1). Hi-C data were created by the DNA Zoo Consortium (www.dnazoo.org). DNA Zoo is supported by Illumina, Inc.; IBM; and the Pawsey Supercomputing Center. The Ramaciotti Centre for Genomics acknowledge infrastructure funding from the Australian Research Council (LE150100031), the Australian Government NCRIS scheme administered by Bioplatforms Australia, and the New South Wales Government RAAP scheme.

Author contributions

JWOB coordinated the project and wrote the initial draft. MAF performed variation analyses. BDR and RJE performed and assisted with the genome assembly, polishing and KAT analysis. LABW and LGK undertook cranial imaging and LGK collected cranial morphometric data. The DNA Zoo initiative, including OD, AO, EA performed and funded the Hi-C experiment. OD and ELA conducted the Hi-C analyses. BC performed the phylogenomic analyses. JK performed the GeMoMa analyses including gene order predictions. OB and KS performed and funded the whole genome bisulphite sequencing and analysis. Eva Chan and Vanessa Hayes collected the Bionano data and performed the analyses. Rob Zammit obtained the initial blood samples and extracted the brain. All authors edited and approved the final manuscript. All authors edited and approved the final manuscript.

References

1. Darwin C. On the origin of species. London: John Murray; 1858.
2. Darwin C. The variation of animals and plants under domestication. New York: Orange Judd & Co; 1868.
3. Ballard JWO and Wilson LAB. The Australian dingo: untamed or feral? Front Zool. 2019;16:19. doi:10.1186/s12983-019-0300-6.
4. Zhang SJ, Wang GD, Ma P, Zhang LL, Yin TT, Liu YH, et al. Genomic regions under selection in the feralization of the dingoes. Nat Comm. 2020;11:671. doi:10.1038/s41467-020-14515-6.
5. Vigne JD. The origins of animal domestication and husbandry: a major change in the history of humanity and the biosphere. C R Biol. 2011;334 3:171-81. doi:10.1016/j.crvi.2010.12.009.
6. Field MA, Yadav S, Dudchenko O, Esvaran M, Rosen BD, Skvortsova K, et al. The Australian dingo is an early offshoot of modern breed dogs. Sci Adv. 2022;8:eabm5944.
7. White J. Journal of a voyage to New South Wales : with sixty-five plates of non descript animals, birds, lizards, serpents, curious cones of trees and other natural productions. London: Debrett, J.; 1790.
8. Meyer FAA. Systematisch-summarische Uebersicht der neuesten zoologischen Entdeckungen in Neuholland und Afrika: nebst zwey andern zoologischen Abhandlungen. Leipzig: Dykische Buchhandlung; 1793.
9. Crowther MS, Fillios M, Colman N and Letnic M. An updated description of the Australian dingo (*Canis dingo* Meyer, 1793). J Zool. 2014;293 3:192-203. doi:10.1111/jzo.12134.
10. Smith BP, Cairns KM, Adams JW, Newsome TM, Fillios M, Deaux EC, et al. Taxonomic status of the Australian dingo: the case for *Canis dingo* Meyer, 1793. Zootaxa. 2019;4564:173-97. doi:10.11646/zootaxa.4564.1.6.
11. Jackson SM, Fleming PJS, Eldridge MDB, Archer M, Ingleby S, Johnson RN, et al. Taxonomy of the dingo: It's an ancient dog. Aust Zool. 2021;41 3:347-57.
12. Mayr E. Genetics and the origin of species. New York: Columbia University Press; 1942.
13. Jackson SM, Fleming PJS, Eldridge MDB, Ingleby S, Flannery T, Johnson RN, et al. The dogma of dingoes-taxonomic status of the dingo: a reply to Smith et al. Zootaxa. 2019;4564 1.
14. Jackson SM, Groves CP, Fleming PJS, Aplin KP, Eldridge MDB, Gonzalez A, et al. The wayward dog: Is the Australian native dog or dingo a distinct species? Zootaxa. 2017;4317 2:201-24. doi:10.11646/zootaxa.4317.2.1.
15. Corbett LK. The dingo in Australia and Asia. Sydney: University of New South Wales Press; 1995.
16. Corbett L. The conservation status of the dingo *Canis lupus dingo* in Australia, with particular reference to New South Wales: threats to pure dingoes and potential solutions. In: Dickman CR and Lunney D, editors. A Symposium on the Dingo Sydney: R Zool Soc NSW; 2001.
17. Corbet L. The Australian dingo. In: Merrick JR, Archer M, Hickey GM and Lee SY, editors. Evolution and biogeography of Australian vertebrates. Oatlands, NSW: Australian Scientific Publishing Ltd.; 2006.
18. Jones E. Hybridisation between the dingo, *Canis lupus dingo*, and the domestic dog, *Canis lupus familiaris*, in Victoria: a critical review. Aust Mammal. 2009;31:1-7.

19. Zhang M, Sun G, Ren L, Yuan H, Dong G, Zhang L, et al. Ancient DNA evidence from China reveals the expansion of Pacific dogs. Mol Biol Evol. 2020;37:1462-9. doi:10.1093/molbev/msz311.
20. Savolainen P, Leitner T, Wilton AN, Matisoo-Smith E and Lundeberg J. A detailed picture of the origin of the Australian dingo, obtained from the study of mitochondrial DNA. Proc Natl Acad Sci USA. 2004;101 33:12387-90. doi:10.1073/pnas.0401814101.
21. Gonzalez A, Clark G, O'Connor S and Matisoo-Smith L. A 3000 yeAr old dog burial in Timor-Leste. Aust Archaeol. 2013;76:13-9.
22. Cairns KM and Wilton AN. New insights on the history of canids in Oceania based on mitochondrial and nuclear data. Genetica. 2016;144 5:553-65. doi:10.1007/s10709-016-9924-z.
23. Cairns KM, Brown SK, Sacks BN and Ballard JWO. Conservation implications for dingoes from the maternal and paternal genome: multiple populations, dog introgression, and demography. Ecol Evol. 2017;7 22:9787-807. doi:10.1002/ece3.3487.
24. Cairns KM, Shannon LM, Koler-Matznick J, Ballard JWO and Boyko AR. Elucidating biogeographical patterns in Australian native canids using genome wide SNPs. PLoS One. 2018;13 6:e0198754. doi:10.1371/journal.pone.0198754.
25. Freedman AH and Wayne RK. Deciphering the origin of dogs: from fossils to genomes. Annu Rev Anim Biosci. 2017;5:281-307. doi:10.1146/annurev-animal-022114-110937.
26. Drake AG and Klingenberg CP. Large-scale diversification of skull shape in domestic dogs: disparity and modularity. Am Nat. 2010;175 3:289-301. doi:10.1086/650372.
27. Edwards RJ, Field MA, Ferguson JM, Dudchenko O, Keilwagen J, Rosen BD, et al. Chromosome-length genome assembly and structural variations of the primal Basenji dog (*Canis lupus familiaris*) genome. BMC Genom. 2021;22 1:188. doi:10.1186/s12864-021-07493-6.
28. Field MA, Rosen BD, Dudchenko O, Chan EKF, Minoche AE, Edwards RJ, et al. Canfam_GSD: De novo chromosome-length genome assembly of the German Shepherd Dog (*Canis lupus familiaris*) using a combination of long reads, optical mapping, and Hi-C. Gigascience. 2020;9 4:giaa027. doi:10.1093/gigascience/giaa027.
29. Ballard JWO, Gardner C, L. Ellem L, Yadav S and R.I. K. Eye-contact and sociability data suggest that Australian dingoes have never been domesticated. Curr Zool. 2021;68 4:423-32.
30. Sluys R. Attaching names to biological species: the use and value of type specimens in systematic zoology and Natural History collections
. Biol Theory. 2021;16:49-61.
31. Koren S, Walenz BP, Berlin K, Miller JR, Bergman NH and Phillippy AM. Canu: scalable and accurate long-read assembly via adaptive k-mer weighting and repeat separation. Genome Res. 2017;27 5:722-36. doi:10.1101/gr.215087.116.
32. PacificBiosciences and GenomicConsensus. https:// github.com/PacificBiosciences/gcpp.
33. Walker BJ, Abeel T, Shea T, Priest M, Abouelliel A, Sakthikumar S, et al. Pilon: an integrated tool for comprehensive microbial variant detection and genome assembly improvement. PLoS One. 2014;9 11:e112963. doi:10.1371/journal.pone.0112963.
34. Robinson JT, Turner D, Durand NC, Thorvaldsdottir H, Mesirov JP and Aiden EL. Juicebox.js provides a cloud-based visualization system for Hi-C data. Cell Syst. 2018;6 2:256-8 e1. doi:10.1016/j.cels.2018.01.001.
35. DNAZoo: Alpine dingo assembly at DNA Zoo. www.dnazoo.org/.

36. Wang C, Wallerman O, Arendt ML, Sundstrom E, Karlsson A, Nordin J, et al. A novel canine reference genome resolves genomic architecture and uncovers transcript complexity. Commun Biol. 2021;4 1:185. doi:10.1038/s42003-021-01698-x.
37. Simao FA, Waterhouse RM, Ioannidis P, Kriventseva EV and Zdobnov EM. BUSCO: assessing genome assembly and annotation completeness with single-copy orthologs. Bioinformatics. 2015;31:3210-2. doi:10.1093/bioinformatics/btv351.
38. Halo JV, Pendleton AL, Shen F, Doucet AJ, Derrien T, Hitte C, et al. Long-read assembly of a Great Dane genome highlights the contribution of GC-rich sequence and mobile elements to canine genomes. Proc Natl Acad Sci USA. 2021;118 11 doi:10.1073/pnas.2016274118.
39. Player RA, Forsyth ER, Verratti KJ, Mohr DW, Scott AF and Bradburne CE. A novel *Canis lupus familiaris* reference genome improves variant resolution for use in breed-specific GWAS. Life Sci Alliance. 2021;4 4 doi:10.26508/lsa.202000902.
40. Jagannathan V, Hitte C, Kidd JM, Masterson P, Murphy TD, Emery S, et al. Dog10K_Boxer_Tasha_1.0: A Long-Read Assembly of the Dog Reference Genome. Genes. 2021;12 6 doi:10.3390/genes12060847.
41. Sinding MS, Gopalakrishnan S, Raundrup K, Dalen L, Threlfall J, Darwin Tree of Life Barcoding c, et al. The genome sequence of the grey wolf, *Canis lupus* Linnaeus 1758. Wellcome Open Res. 2021:310. doi:10.12688/wellcomeopenres.17332.1.
42. Rhie A, Walenz BP, Koren S and Phillippy AM. Merqury: reference-free quality, completeness, and phasing assessment for genome assemblies. Genome Biol. 2020;21 1:245. doi:10.1186/s13059-020-02134-9.
43. Krzywinski M, Schein J, Birol I, Connors J, Gascoyne R, Horsman D, et al. Circos: an information aesthetic for comparative genomics. Genome Res. 2009;19:1639-45. doi:10.1101/gr.092759.109.
44. Marcais G, Delcher AL, Phillippy AM, Coston R, Salzberg SL and Zimin A. MUMmer4: A fast and versatile genome alignment system. PLoS Comput Biol. 2018;14 1:e1005944. doi:10.1371/journal.pcbi.1005944.
45. Sedlazeck FJ, Rescheneder P, Smolka M, Fang H, Nattestad M, von Haeseler A, et al. Accurate detection of complex structural variations using single-molecule sequencing. Nat Methods. 2018;15:461-8. doi:10.1038/s41592-018-0001-7.
46. Waardenberg AJ and Field MA. consensusDE: an R package for assessing consensus of multiple RNA-seq algorithms with RUV correction. PeerJ. 2019;7:e8206. doi:10.7717/peerj.8206.
47. Zhou ZW, Yu ZG, Huang XM, Liu JS, Guo YX, Chen LL, et al. GenomeSyn: A bioinformatics tool for visualizing genome synteny and structural variations. J Genet Genom. 2022; doi:10.1016/j.jgg.2022.03.013.
48. Keilwagen J, Hartung F and Grau J. GeMoMa: Homology-Based Gene Prediction Utilizing Intron Position Conservation and RNA-seq Data. Methods Mol Biol. 2019;1962:161-77. doi:10.1007/978-1-4939-9173-0_9.
49. Glazko G, Gordon A and Mushegian A. The choice of optimal distance measure in genome-wide datasets. Bioinformatics. 2005;21 Suppl 3:iii3-11. doi:10.1093/bioinformatics/bti1201.
50. Savolainen P, Arvestad L and Lundeberg J. mtDNA tandem repeats in domestic dogs and wolves: mutation mechanism studied by analysis of the sequence of imperfect repeats. Mol Biol Evol. 2000;17:474-88. doi:10.1093/oxfordjournals.molbev.a026328.
51. Marshall AS and Jones NS. Discovering cellular mitochondrial heteroplasmy heterogeneity with single cell RNA and ATAC sequencing. Biology (Basel). 2021;10 6 doi:10.3390/biology10060503.

52. Zuker M. Mfold web server for nucleic acid folding and hybridization prediction. Nuc Acids Res. 2003;31 13:3406-15. doi:10.1093/nar/gkg595.
53. Leigh JW and Bryant D. Popart: full-feature software for haplotype network construction. Methods Ecol Evol. 2015;6:1110-6.
54. Freedman AH, Gronau I, Schweizer RM, Ortega-Del Vecchyo D, Han E, Silva PM, et al. Genome sequencing highlights the dynamic early history of dogs. PLoS Genet. 2014;10 1:e1004016. doi:10.1371/journal.pgen.1004016.
55. Greig K, Gosling A, Collins CJ, Boocock J, McDonald K, Addison DJ, et al. Complex history of dog (*Canis familiaris*) origins and translocations in the Pacific revealed by ancient mitogenomes. Sci Rep. 2018;8 1:9130. doi:10.1038/s41598-018-27363-8.
56. Pang JF, Kluetsch C, Zou XJ, Zhang AB, Luo LY, Angleby H, et al. mtDNA data indicate a single origin for dogs south of Yangtze River, less than 16,300 years ago, from numerous wolves. Mol Biol Evol. 2009;26 12:2849-64. doi:10.1093/molbev/msp195.
57. Thalmann O, Shapiro B, Cui P, Schuenemann VJ, Sawyer SK, Greenfield DL, et al. Complete mitochondrial genomes of ancient canids suggest a European origin of domestic dogs. Science. 2013;342:871-4. doi:10.1126/science.1243650.
58. Urich MA, Nery JR, Lister R, Schmitz RJ and Ecker JR. MethylC-seq library preparation for base-resolution whole-genome bisulfite sequencing. Nat Protoc. 2015;10 3:475-83. doi:10.1038/nprot.2014.114.
59. Meissner A, Mikkelsen TS, Gu H, Wernig M, Hanna J, Sivachenko A, et al. Genome-scale DNA methylation maps of pluripotent and differentiated cells. Nature. 2008;454 7205:766-70. doi:10.1038/nature07107.
60. Bogdanovic O, Smits AH, de la Calle Mustienes E, Tena JJ, Ford E, Williams R, et al. Active DNA demethylation at enhancers during the vertebrate phylotypic period. Nat Genet. 2016;48 4:417-26. doi:10.1038/ng.3522.
61. Burger L, Gaidatzis D, Schubeler D and Stadler MB. Identification of active regulatory regions from DNA methylation data. Nucleic Acids Res. 2013;41 16:e155. doi:10.1093/nar/gkt599.
62. Stadler MB, Murr R, Burger L, Ivanek R, Lienert F, Scholer A, et al. DNA-binding factors shape the mouse methylome at distal regulatory regions. Nature. 2011;480 7378:490-5. doi:10.1038/nature10716.
63. Mo A, Mukamel EA, Davis FP, Luo C, Henry GL, Picard S, et al. Epigenomic signatures of neuronal diversity in the mammalian brain. Neuron. 2015;86 6:1369-84. doi:10.1016/j.neuron.2015.05.018.
64. Gollan K. *Prehistoric dingo*. Australian National University, Canberra, 1982.
65. Koungoulos.K. Old dogs, new tricks: 3D geometric analysis of cranial morphology supports ancient population substructure in the Australian dingo. Zoomorphology. 2020;139:263-75.
66. Fedorov A, Beichel R, Kalpathy-Cramer J, Finet J, Fillion-Robin JC, Pujol S, et al. 3D Slicer as an image computing platform for the quantitative imaging network. Magn Reson Imaging. 2012;30 9:1323-41. doi:10.1016/j.mri.2012.05.001.
67. Hager ER, Harringmeyer OS, Wooldridge TB, Theingi S, Gable JT, McFadden S, et al. A chromosomal inversion contributes to divergence in multiple traits between deer mouse ecotypes. Science. 2022;377 6604:399-405.
68. Forman OP, Hitti RJ, Pettitt L, Jenkins CA, O'Brien DP, Shelton GD, et al. An inversion disrupting FAM134B Is associated with sensory neuropathy in the Border Collie dog breed. G3. 2016;6 9:2687-92. doi:10.1534/g3.116.027896.

69. Tan S, Cardoso-Moreira M, Shi W, Zhang D, Huang J, Mao Y, et al. LTR-mediated retroposition as a mechanism of RNA-based duplication in metazoans. Genome Res. 2016;26:1663-75. doi:10.1101/gr.204925.116.
70. Pajic P, Pavlidis P, Dean K, Neznanova L, Romano RA, Garneau D, et al. Independent amylase gene copy number bursts correlate with dietary preferences in mammals. Elife. 2019;8 doi:10.7554/eLife.44628.
71. Arendt M, Cairns KM, Ballard JWO, Savolainen P and Axelsson E. Diet adaptation in dog reflects spread of prehistoric agriculture. Heredity. 2016;117 5:301-6. doi:10.1038/hdy.2016.48.
72. Vicoso B and Charlesworth B. Evolution on the X chromosome: unusual patterns and processes. Nat Rev Genet. 2006;7 8:645-53. doi:10.1038/nrg1914.
73. Mank JE, Vicoso B, Berlin S and Charlesworth B. Effective population size and the faster-X effect: empirical results and their interpretation. Evolution. 2010;64 3:663-74. doi:10.1111/j.1558-5646.2009.00853.x.
74. Plassais J, Rimbault M, Williams FJ, Davis BW, Schoenebeck JJ and Ostrander EA. Analysis of large versus small dogs reveals three genes on the canine X chromosome associated with body weight, muscling and back fat thickness. PLoS Genet. 2017;13 3:e1006661. doi:10.1371/journal.pgen.1006661.
75. Basu U, Bostwick AM, Das K, Dittenhafer-Reed KE and Patel SS. Structure, mechanism, and regulation of mitochondrial DNA transcription initiation. J Biol Chem. 2020;295 52:18406-25. doi:10.1074/jbc.REV120.011202.
76. Bjornerfeldt S, Webster MT and Vila C. Relaxation of selective constraint on dog mitochondrial DNA following domestication. Genome Res. 2006;16 8:990-4. doi:10.1101/gr.5117706.
77. Milham PT, P. Relative antiquity of human occupation and extinct fauna at Madura Cave, Southeastern Western Australia. Mankind. 1976;10:175-80.
78. Schubeler D. Function and information content of DNA methylation. Nature. 2015;517 7534:321-6. doi:10.1038/nature14192.
79. Wewer Albrechtsen NJ, Kuhre RE, Pedersen J, Knop FK and Holst JJ. The biology of glucagon and the consequences of hyperglucagonemia. Biomark Med. 2016;10 11:1141-51. doi:10.2217/bmm-2016-0090.
80. Insuela DBR, Azevedo CT, Coutinho DS, Magalhaes NS, Ferrero MR, Ferreira TPT, et al. Glucagon reduces airway hyperreactivity, inflammation, and remodeling induced by ovalbumin. Sci Rep. 2019;9 1:6478. doi:10.1038/s41598-019-42981-6.
81. Yang Q, Tang J, Pei R, Gao X, Guo J, Xu C, et al. Host HDAC4 regulates the antiviral response by inhibiting the phosphorylation of IRF3. J Mol Cell Biol. 2019;11:158-69. doi:10.1093/jmcb/mjy035.
82. Cui H, Moore J, Ashimi SS, Mason BL, Drawbridge JN, Han S, et al. Eating disorder predisposition is associated with ESRRA and HDAC4 mutations. J Clin Invest. 2013;123 11:4706-13. doi:10.1172/JCI71400.
83. Radford CG, Letnic M, Fillios M and Crowther MS. An assessment of the taxonomic status of wild canids in south-eastern New South Wales: phenotypic variation in dingoes. Aust J Zool. 2012;60:73-80.
84. Stephens D, Wilton AN, Fleming PJ and Berry O. Death by sex in an Australian icon: a continent-wide survey reveals extensive hybridization between dingoes and domestic dogs. Mol Ecol. 2015;24 22:5643-56. doi:10.1111/mec.13416.
85. Cairns KM, Crother MS, Nesbit B and Letnik M. The myth of wild dogs in Australia: are there any out there? Aust Mamm. 2020;44:67-75.

86. Geiger M, Evin A, Sanchez-Villagra MR, Gascho D, Mainini C and Zollikofer CPE. Neomorphosis and heterochrony of skull shape in dog domestication. Sci Rep. 2017;7 1:13443. doi:10.1038/s41598-017-12582-2.
87. Balcarcel AM, Geiger M, Clauss M and Sanchez-Villagra MR. The mammalian brain under domestication: discovering patterns after a century of old and new analyses. J Exp Zool B Mol Dev Evol. 2022;338 8:460-83. doi:10.1002/jez.b.23105.
88. Klatt B. Über die veränderung der schädelkapazität in der somestikation. Sitzungsbericht der Gesellschaft naturforschender Freunde. 1912:3.
89. Röhrs M and Ebinger P. Die Berteilung von Hirngrossenunterschieden. Journal of Zoological Systematics and Evolutionary Research. 1978;16:1-14.
90. Kruska D. Mammalian domestication and its effect on brain structure and behavior. In: Jerison H, J, and Jerison I, editors. Intelligence and Evolutionary Biology. New York: Academic Press; 1988.
91. Brusini I, Carneiro M, Wang C, Rubin CJ, Ring H, Afonso S, et al. Changes in brain architecture are consistent with altered fear processing in domestic rabbits. Proc Natl Acad Sci USA. 2018;115 28:7380-5. doi:10.1073/pnas.1801024115.
92. Kruska DC. On the evolutionary significance of encephalization in some eutherian mammals: effects of adaptive radiation, domestication, and feralization. Brain Behav Evol. 2005;65 2:73-108. doi:10.1159/000082979.
93. Barrickman NL, Bastian ML, Isler K and van Schaik CP. Life history costs and benefits of encephalization: a comparative test using data from long-term studies of primates in the wild. J Hum Evol. 2008;54 5:568-90. doi:10.1016/j.jhevol.2007.08.012.
94. Rohrs M and Ebinger P. Wild is not really wild: brain weight of wild domestic mammals. Berl Munch Tierarztl Wochenschr. 1999;112 6-7:234-8.
95. Kruska D and M. R. Comparative-quantitative investigations on brains of feral pigs from the Galapagos Islands and of European domestic pigs. Z Anat Entwicklungsgesch. 1974;144:61–73.
96. Lord KA, Larson G and Karlsson EK. Brain size does not rescue domestication syndrome. Trends Ecol Evol. 2020;35 12:1061-2. doi:10.1016/j.tree.2020.10.004.
97. Liu YH, Wang L, Xu T, Guo X, Li Y, Yin TT, et al. Whole-genome sequencing of African dogs provides Insights into adaptations against tropical parasites. Mol Biol Evol. 2018;35 2:287-98. doi:10.1093/molbev/msx258.
98. Erin NI, Benesh DP, Henrich T, Samonte IE, Jakobsen PJ and Kalbe M. Examining the role of parasites in limiting unidirectional gene flow between lake and river sticklebacks. J Anim Ecol. 2019;88 12:1986-97. doi:10.1111/1365-2656.13080.
99. Bradley C. Venomous bites and stings in Australia to 2005. In: Welfare AIoHa, (ed.). Canberra: Australian Government, 2014, p. 119.
100. Gulevich RG and et al. Effect of selection for behavior on pituitary-adrenal axis and proopiomelanocortin gene expression in silver foxes (Vulpes vulpes). Physiol Behav. 2004;82 2-3:513-8. doi:10.1016/j.physbeh.2004.04.062.
101. Heyne HO, Lautenschläger S, Nelson R, Besnier F, Rotival M, Cagan A, et al. Genetic influences on brain gene expression in rats selected for tameness and aggression. Genetics. 2014;198 3:1277-90. doi:10.1534/genetics.114.168948.
102. Matsumoto Y, Nagayama.H., Nakaoka H, Toyoda A, Goto T and Koide T. Combined change of behavioral traits for domestication and gene-networks in mice selectively bred for active tameness. Genes Brain Behav. 2021;20:e12721. doi:10.1111/gbb.12721.

103. Albert FW and et al. A comparison of brain gene expression levels in domesticated and wild animals. PLoS Genet. 2012;8 9:e1002962. doi:10.1371/journal.pgen.1002962.

104. Wilton AN. DNA methods of assessing dingo purity. . Sydney: R. Zool. Soc. N.S.W.; 2001.

105. Deaux EC, Allen AP, Clarke JA and Charrier I. Concatenation of 'alert' and 'identity' segments in dingoes' alarm calls. Sci Rep. 2016;6:30556. doi:10.1038/srep30556.

106. Rao SS, Huntley MH, Durand NC, Stamenova EK, Bochkov ID, Robinson JT, et al. A 3D map of the human genome at kilobase resolution reveals principles of chromatin looping. Cell. 2014;159 7:1665-80. doi:10.1016/j.cell.2014.11.021.

107. Yeo S, Coombe L, Warren RL, Chu J and Birol I. ARCS: scaffolding genome drafts with linked reads. Bioinformatics. 2018;34:725-31. doi:10.1093/bioinformatics/btx675.

108. Chromium X: 10X Genomics linked-read alignment,variant calling, phasing, and structural variant calling https://support.10xgenomics.com/genome-exome/software/pipelines/latest/what-is-long-ranger (2020). Accessed 2020.

109. Li H. Minimap2: pairwise alignment for nucleotide sequences. Bioinformatics. 2018;34 18:3094-100. doi:10.1093/bioinformatics/bty191.

110. Vaser R, Sovic I, Nagarajan N and Sikic M. Fast and accurate de novo genome assembly from long uncorrected reads. Genome Res. 2017;27 5:737-46. doi:10.1101/gr.214270.116.

111. Durand NC, Robinson JT, Shamim MS, Machol I, Mesirov JP, Lander ES, et al. Juicebox provides a visualization system for Hi-C contact maps with unlimited zoom. Cell Syst. 2016;3 1:99-101. doi:10.1016/j.cels.2015.07.012.

112. Dudchenko O, Batra SS, Omer AD, Nyquist SK, Hoeger M, Durand NC, et al. *De novo* assembly of the *Aedes aegypti* genome using Hi-C yields chromosome-length scaffolds. Science. 2017;356 6333:92-5. doi:10.1126/science.aal3327.

113. Dudchenko O, Shamim MS, Batra SS, Durand NC, Musial NT, Mostofa R, et al. The Juicebox Assembly Tools module facilitates *de novo* assembly of mammalian genomes with chromosome-length scaffolds for under $1000. bioRxiv. 2018:254797. doi:10.1101/254797.

114. English AC, Richards S, Han Y, Wang M, Vee V, Qu J, et al. Mind the gap: upgrading genomes with Pacific Biosciences RS long-read sequencing technology. PLoS One. 2012;7 11:e47768. doi:10.1371/journal.pone.0047768.

115. Altschul SF, Gish W, Miller W, Myers EW and Lipman DJ. Basic local alignment search tool. J Mol Biol. 1990;215 3:403-10. doi:10.1016/S0022-2836(05)80360-2.

116. Finn RD, Clements J and Eddy SR. HMMER web server: interactive sequence similarity searching. Nucleic Acids Res. 2011;39 Web Server issue:W29-37. doi:10.1093/nar/gkr367.

117. Levy KE, Mirdita M and Soding J. MetaEuk-sensitive, high-throughput gene discovery, and annotation for large-scale eukaryotic metagenomics. Microbiome. 2020;8 1:48. doi:10.1186/s40168-020-00808-x.

118. Hoeppner MP, Lundquist A, Pirun M, Meadows JR, Zamani N, Johnson J, et al. An improved canine genome and a comprehensive catalogue of coding genes and non-coding transcripts. PLoS One. 2014;9 3:e91172. doi:10.1371/journal.pone.0091172.

119. Edwards R: PAFScaff biotools. https://bio.tools/PAFScaff_Pairwise_mApping_Format_reference-based_scaffold_anchoring_and_super-scaffolding. (2020). Accessed Nov 1, 2019.

120. Chakraborty M, Emerson JJ, Macdonald SJ and Long AD. Structural variants exhibit widespread allelic heterogeneity and shape variation in complex traits. Nat Commun. 2019;10 1:4872. doi:10.1038/s41467-019-12884-1.
121. Schliep K, Potts AJ, Morrison DA and Grimm GW. Intertwining phylogenetic trees and networks. Methods Ecol Evol. 2017;8 10:1212-20.
122. Hammer O, Harper DAT and PD. R. PAST: Paleontological software package for education and data ananlysis. Palaeontol Electron. 2001;4:9pp.
123. Davey NE, Shields DC and Edwards RJ. SLiMDisc: short, linear motif discovery, correcting for common evolutionary descent. Nuc Acids Res. 2006;34 12:3546-54. doi:10.1093/nar/gkl486.
124. Li H and Durbin R. Fast and accurate short read alignment with Burrows-Wheeler transform. Bioinformatics. 2009;25 14:1754-60. doi:10.1093/bioinformatics/btp324.
125. Kundu R, Casey J and Sung W-K. HyPo: Super fast & accurate polisher for long read genome assemblies. bioRxiv. 2019:doi: 10.1101/2019.12.19.882506. doi:10.1101/2019.12.19.882506.
126. Donath A, Juhling F, Al-Arab M, Bernhart SH, Reinhardt F, Stadler PF, et al. Improved annotation of protein-coding genes boundaries in metazoan mitochondrial genomes. Nucleic Acids Res. 2019;47 20:10543-52. doi:10.1093/nar/gkz833.
127. Urich MA, Nery JR, Lister R, Schmitz RJ and Ecker JR. MethylC-seq library preparation for base-resolution whole-genome bisulfite sequencing. Nat Protoc. 2015;10 3:475-83. doi:10.1038/nprot.2014.114.
128. Lautenschlager S. Reconstructing the past: methods and techniques for the digital restoration of fossils. R Soc Open Sci. 2016;3 10:160342. doi:10.1098/rsos.160342.
129. Klingenberg CP. MorphoJ: an integrated software package for geometric morphometrics. Mol Ecol Resour. 2011;11 2:353-7. doi:10.1111/j.1755-0998.2010.02924.x.
130. Rohlf F and Slice D. Extensions of the procrustes method for the optimal superimposition of landmarks. Syst Zool. 1990;39.
