# Supplementary figures and images for: The Australasian dingo archetype: de novo chromosome-length genome assembly, DNA methylome, and cranial morphology
Source: Gigascience. 2023 Mar 28;12:giad018. doi: 10.1093/gigascience/giad018 (PMC10353722; doi:10.1093/gigascience/giad018)

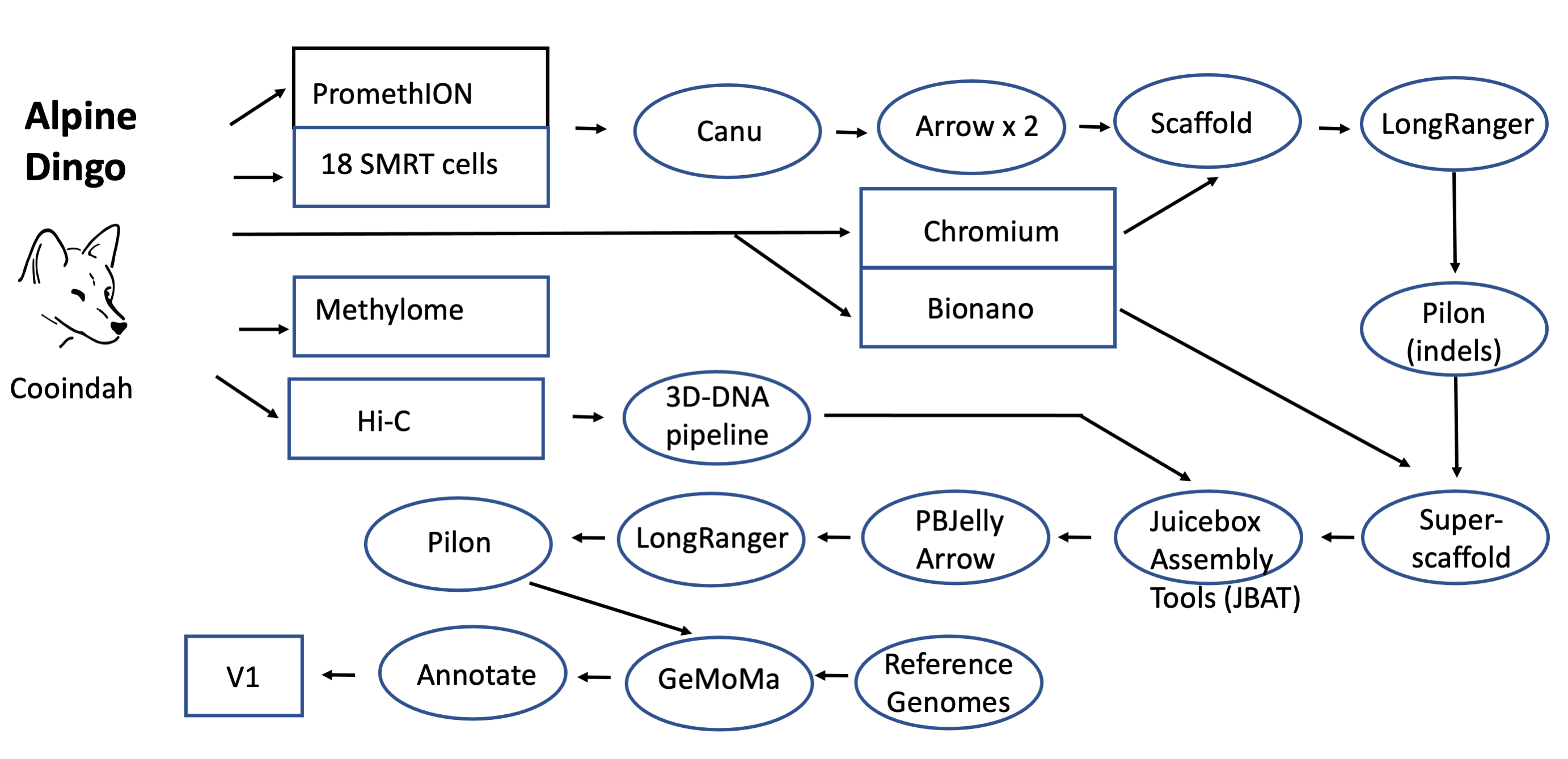

Supplement: giad018_Supplemental_Figures [file giad018_supplemental_figures.zip › Suppl Figure 1.tiff]

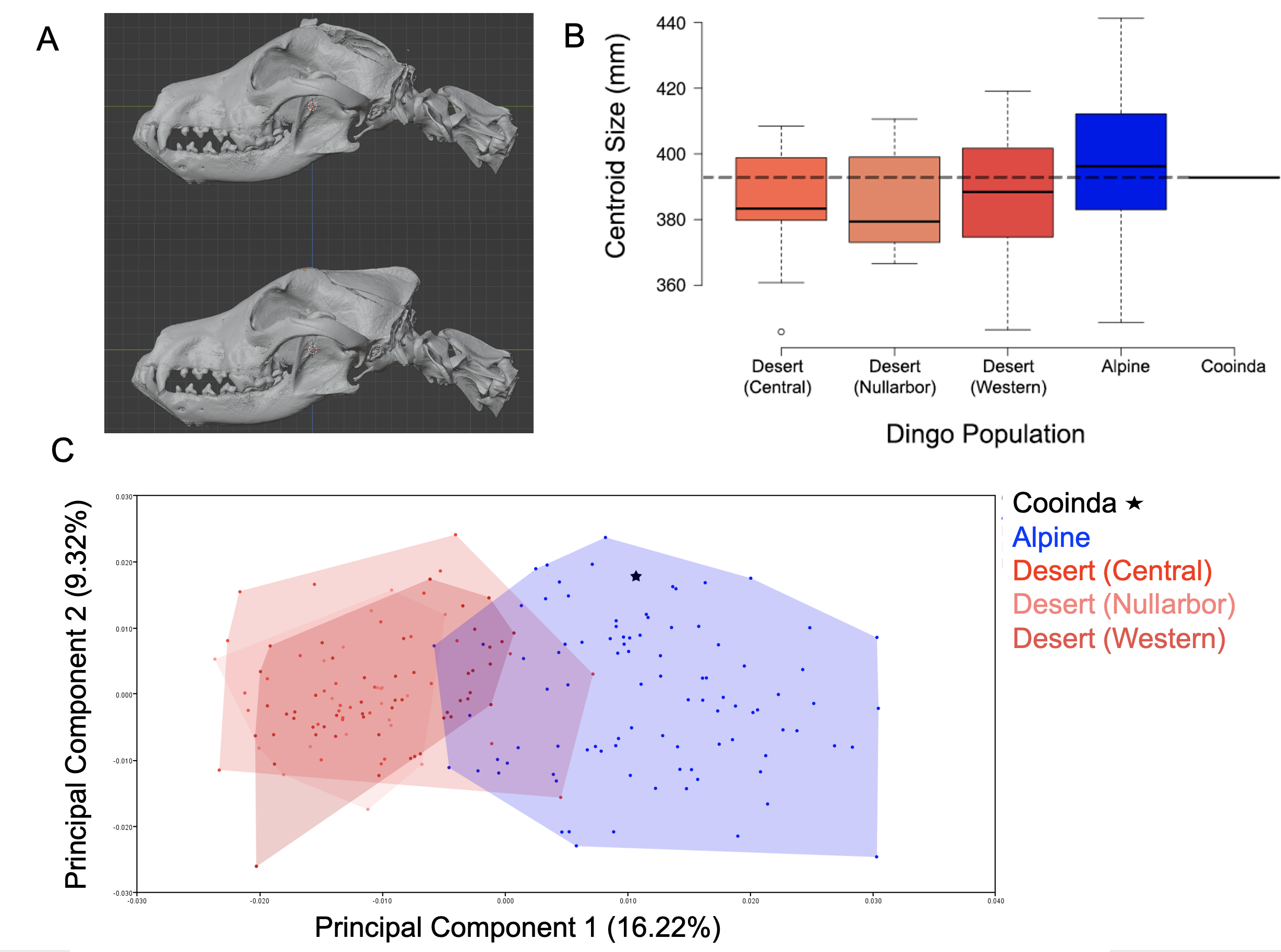

Supplement: giad018_Supplemental_Figures [file giad018_supplemental_figures.zip › Suppl Figure 10.tiff]

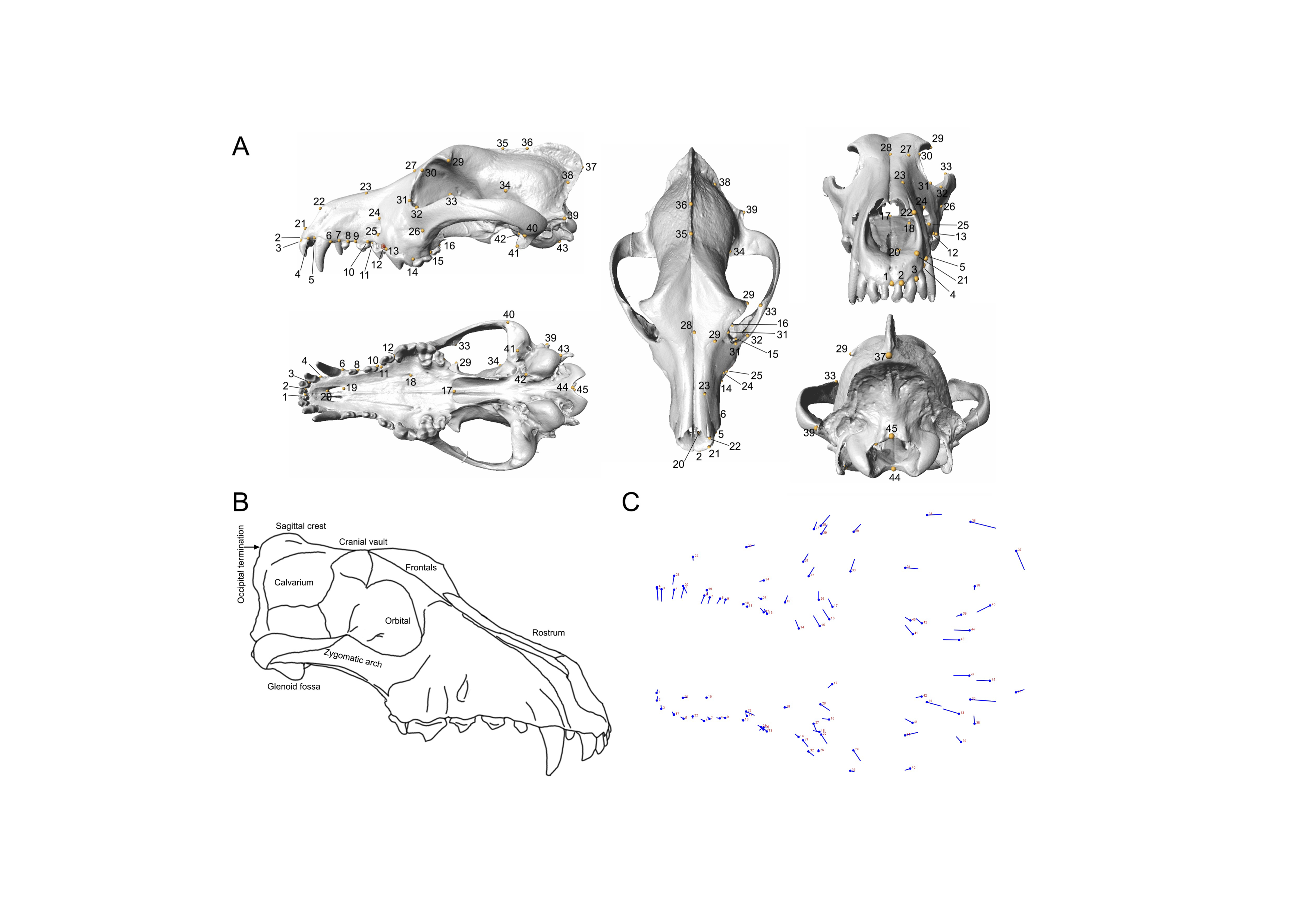

Supplement: giad018_Supplemental_Figures [file giad018_supplemental_figures.zip › Suppl Figure 11.tiff]

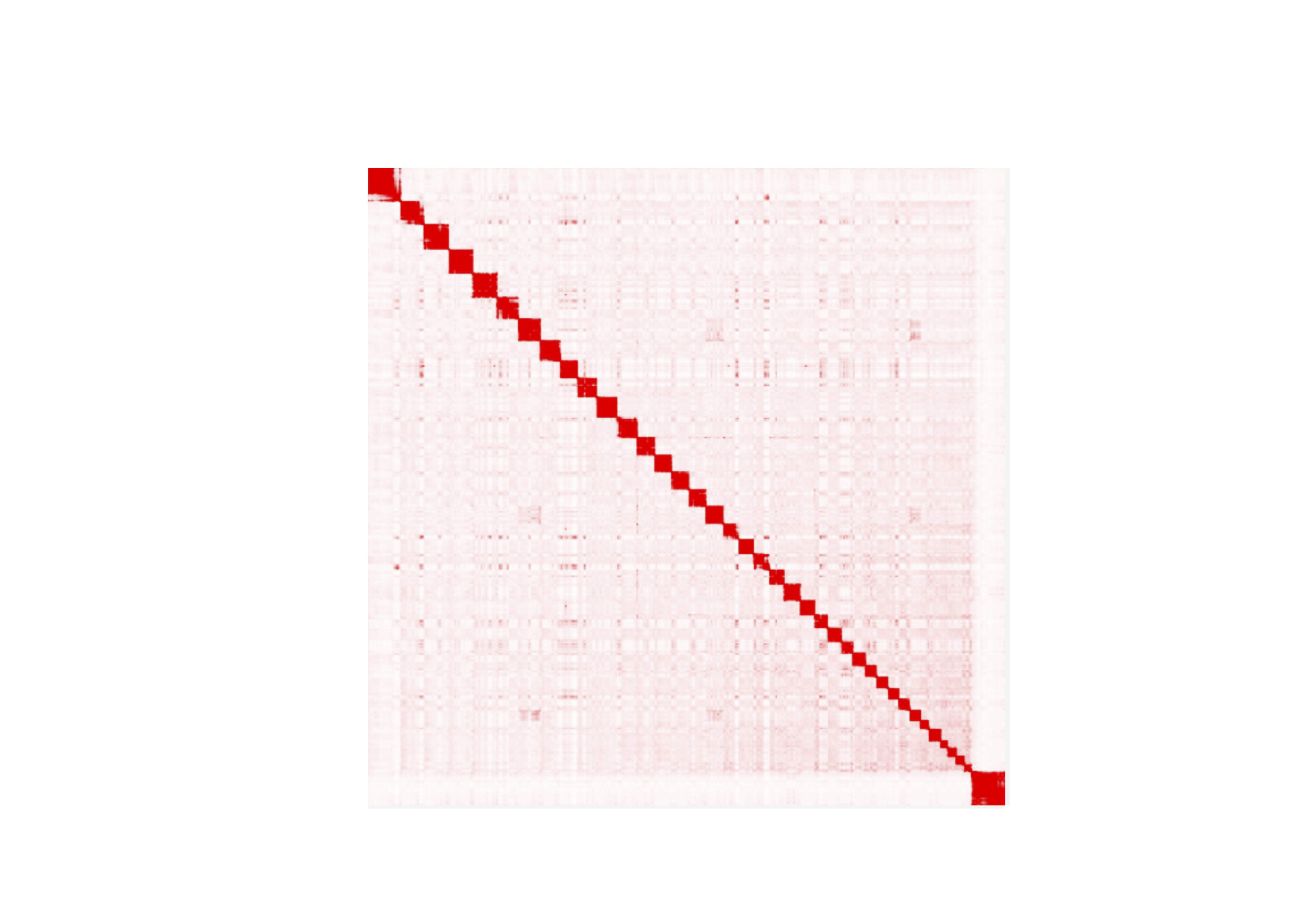

Supplement: giad018_Supplemental_Figures [file giad018_supplemental_figures.zip › Suppl Figure 2.tiff]

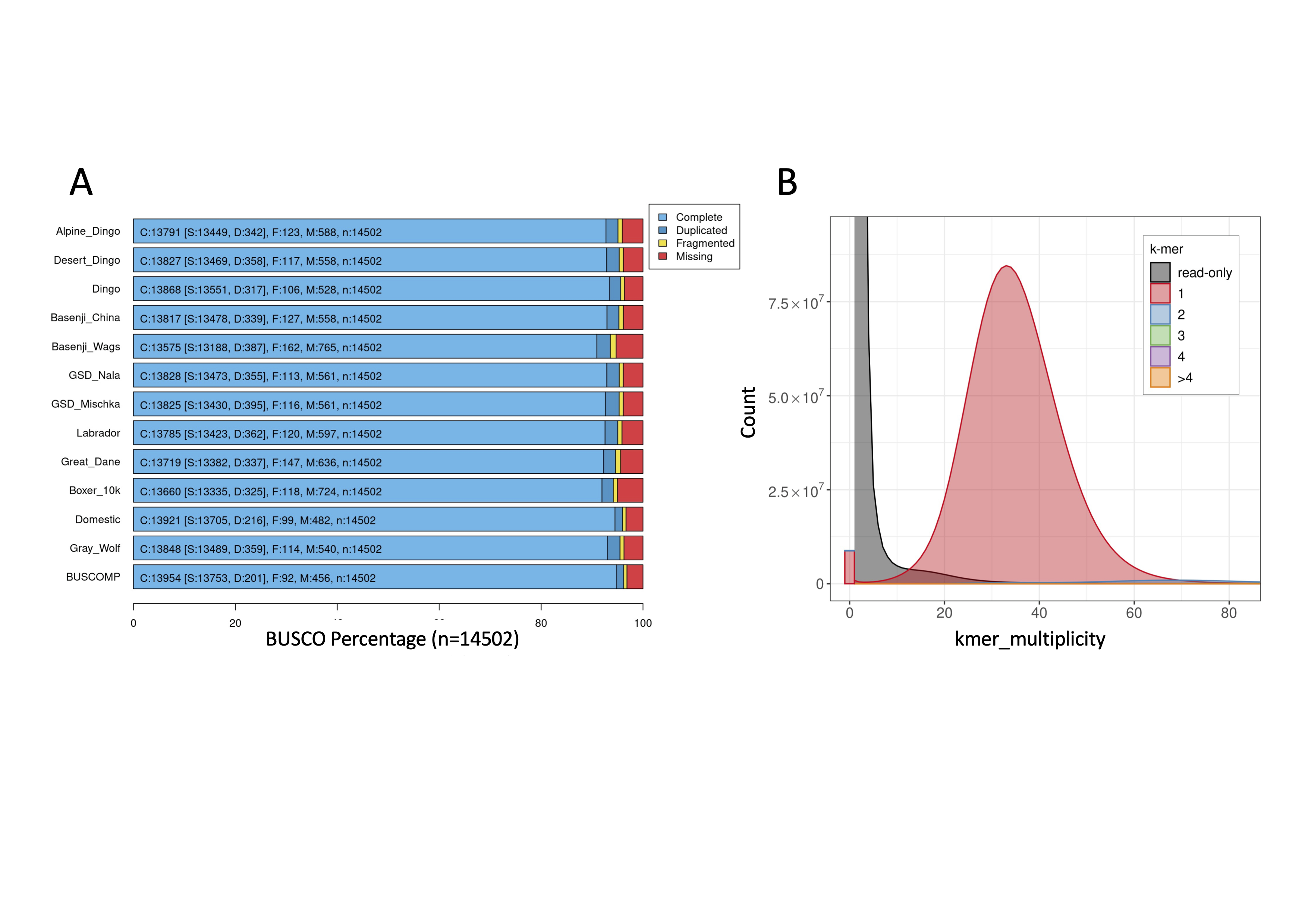

Supplement: giad018_Supplemental_Figures [file giad018_supplemental_figures.zip › Suppl Figure 3.tiff]

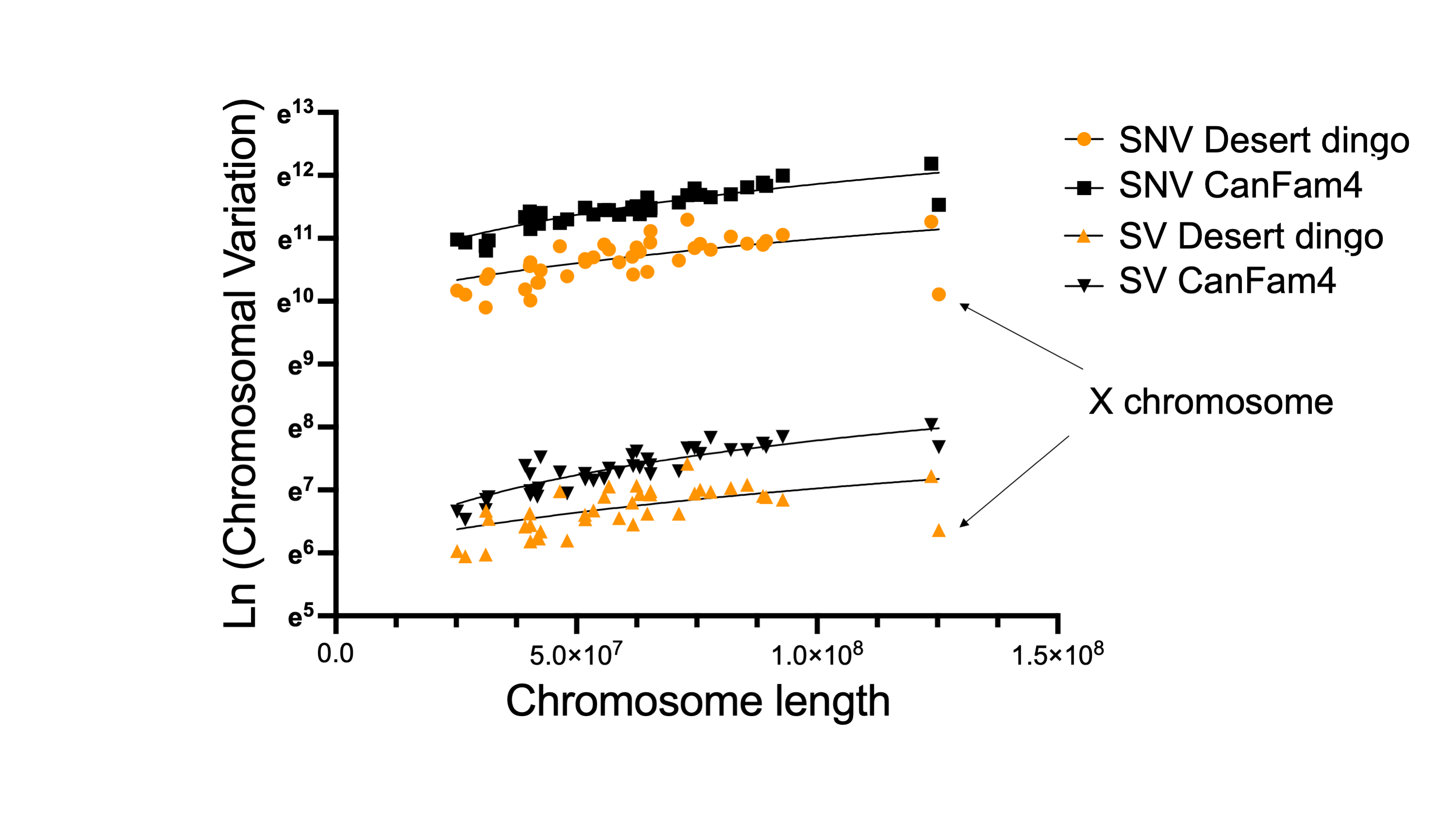

Supplement: giad018_Supplemental_Figures [file giad018_supplemental_figures.zip › Suppl Figure 4.tiff]

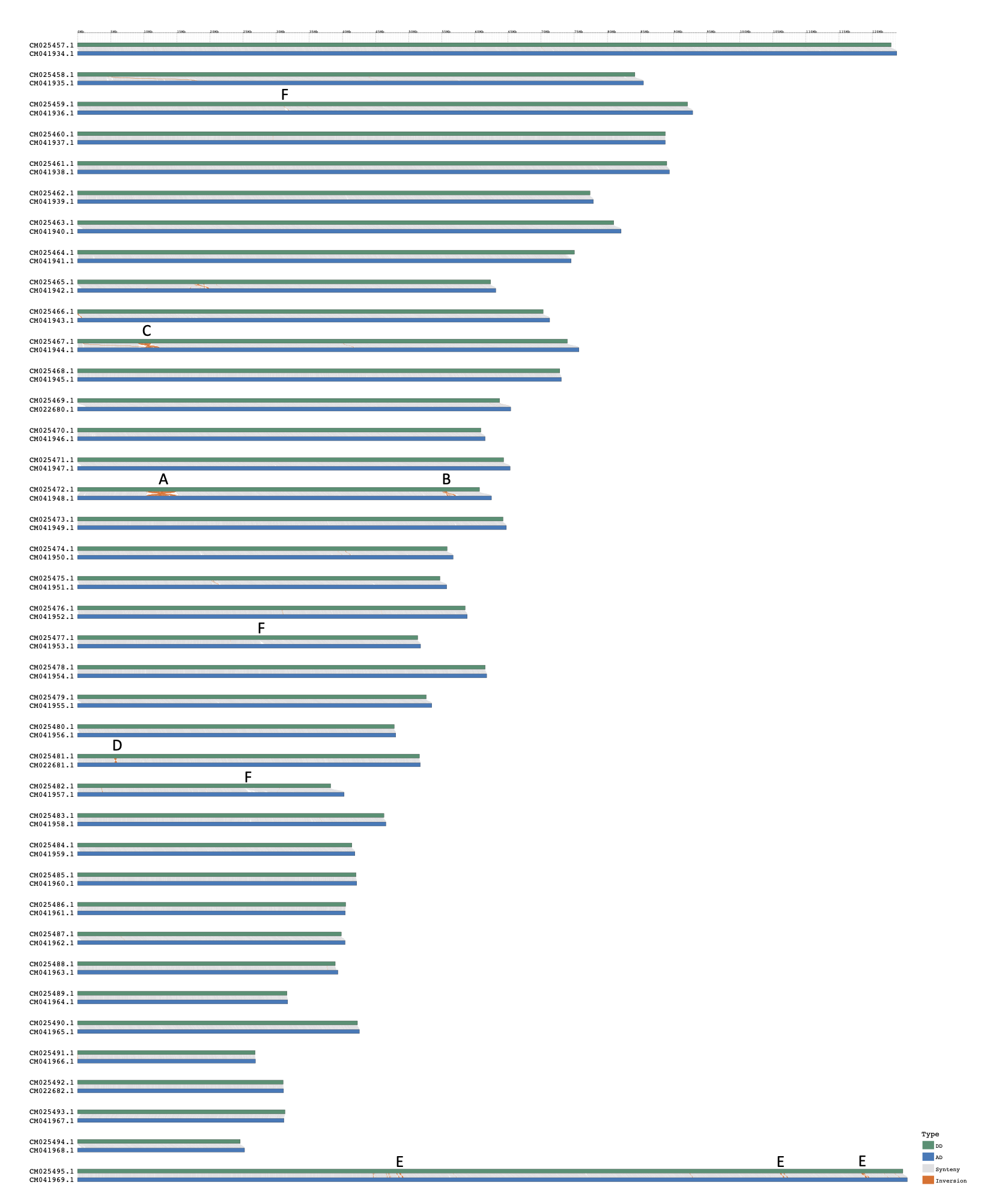

Supplement: giad018_Supplemental_Figures [file giad018_supplemental_figures.zip › Suppl Figure 5.tiff]

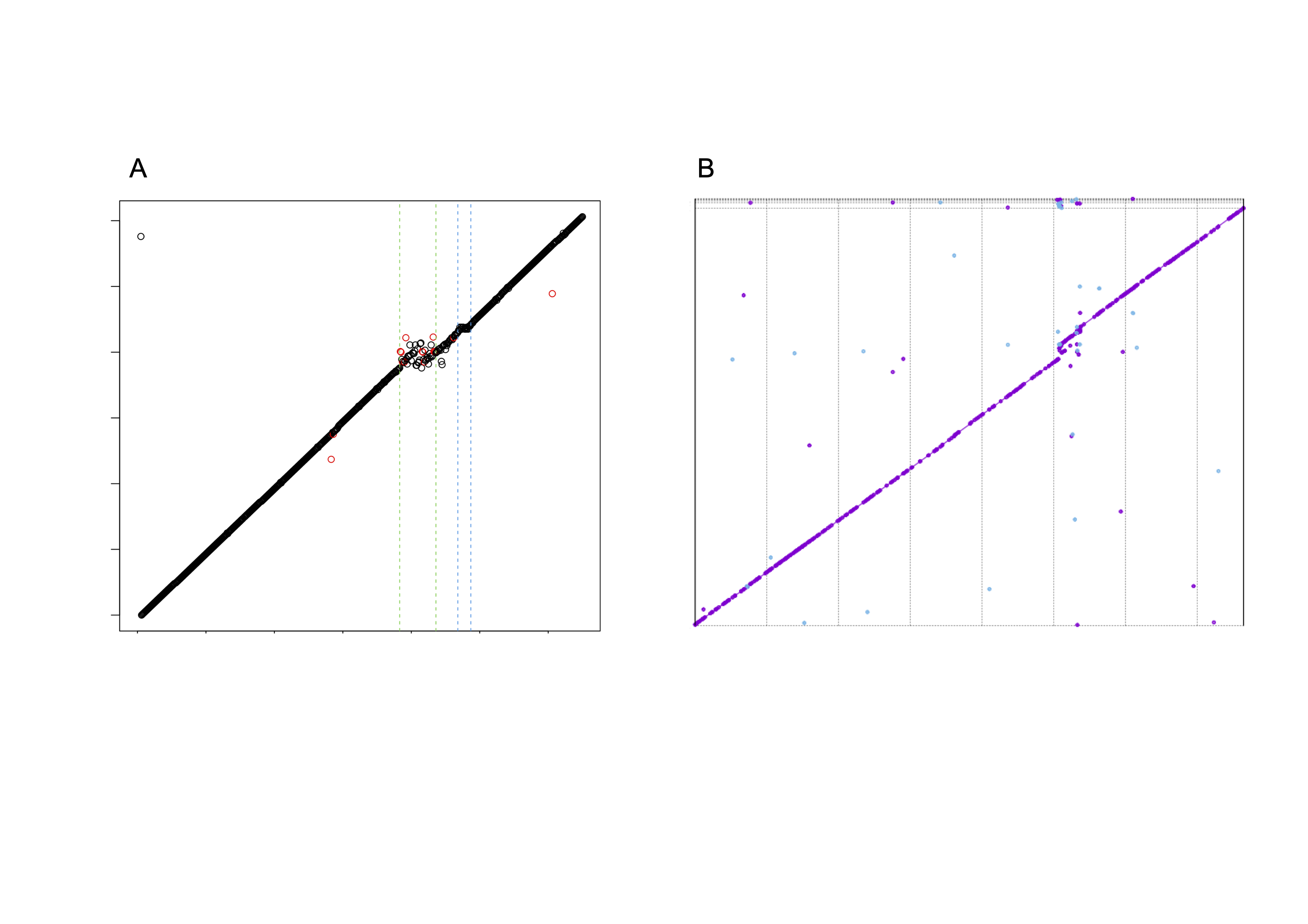

Supplement: giad018_Supplemental_Figures [file giad018_supplemental_figures.zip › Suppl Figure 6.tiff]

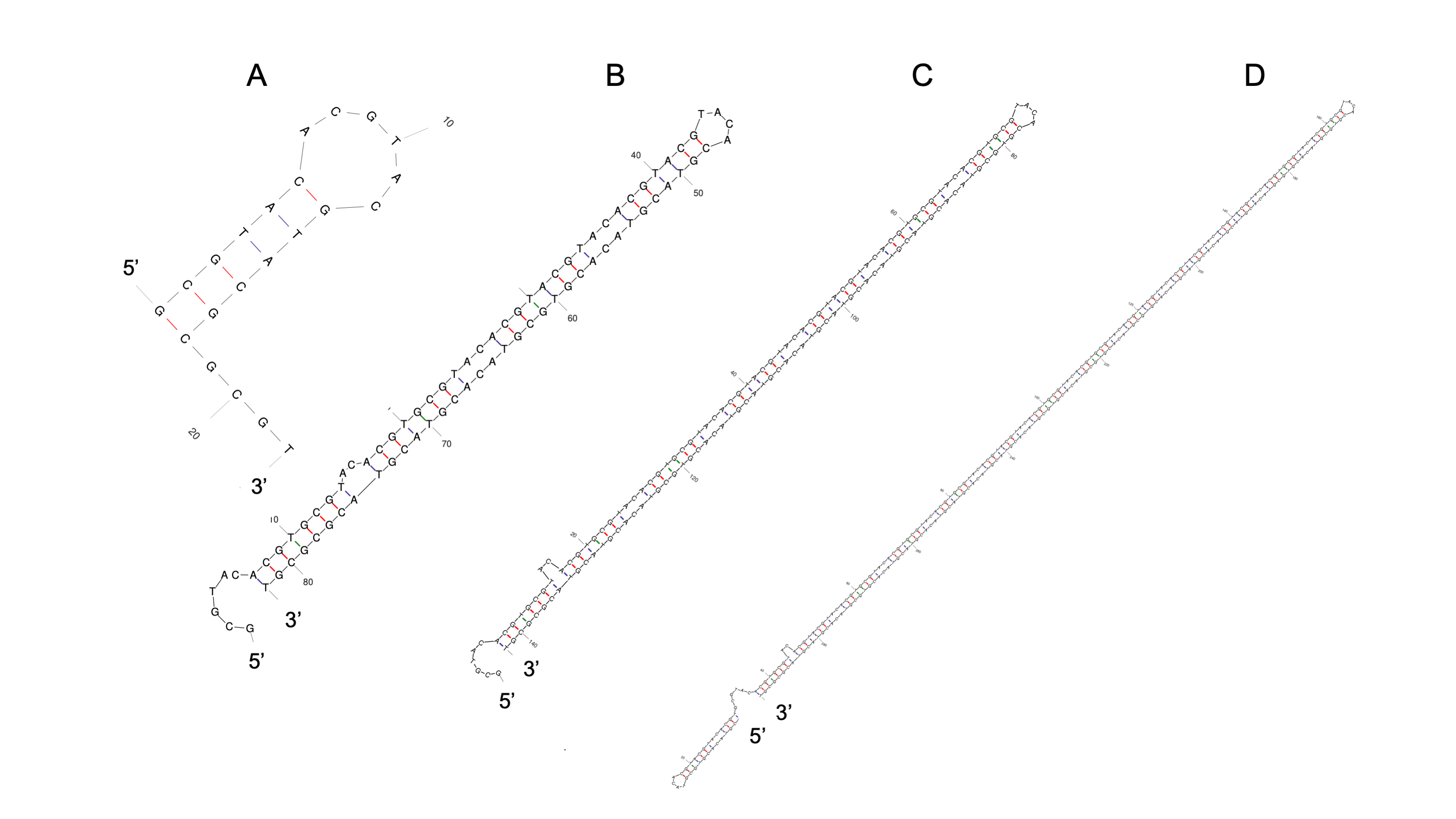

Supplement: giad018_Supplemental_Figures [file giad018_supplemental_figures.zip › Suppl Figure 7.tiff]

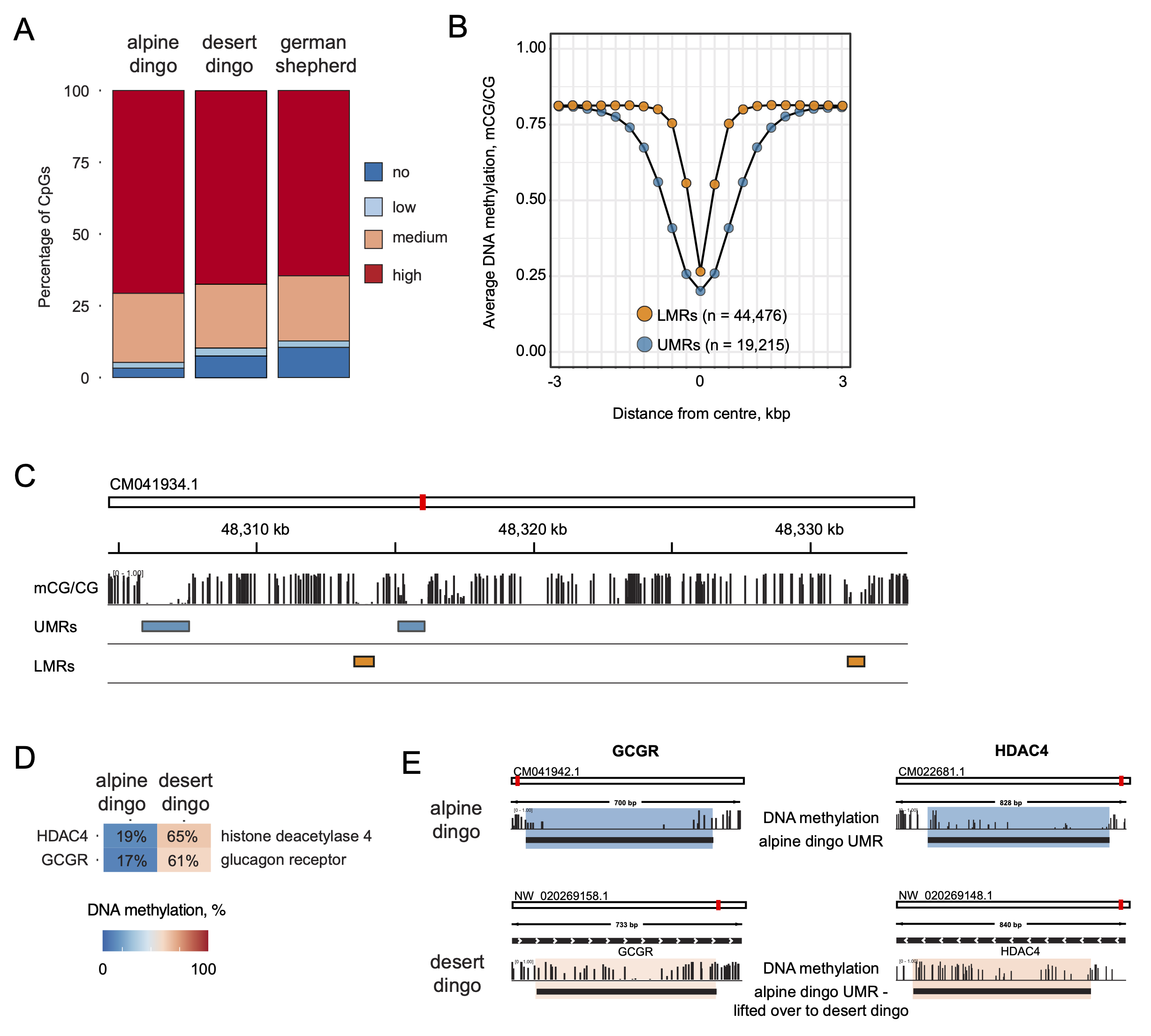

Supplement: giad018_Supplemental_Figures [file giad018_supplemental_figures.zip › Suppl Figure 8.tiff]

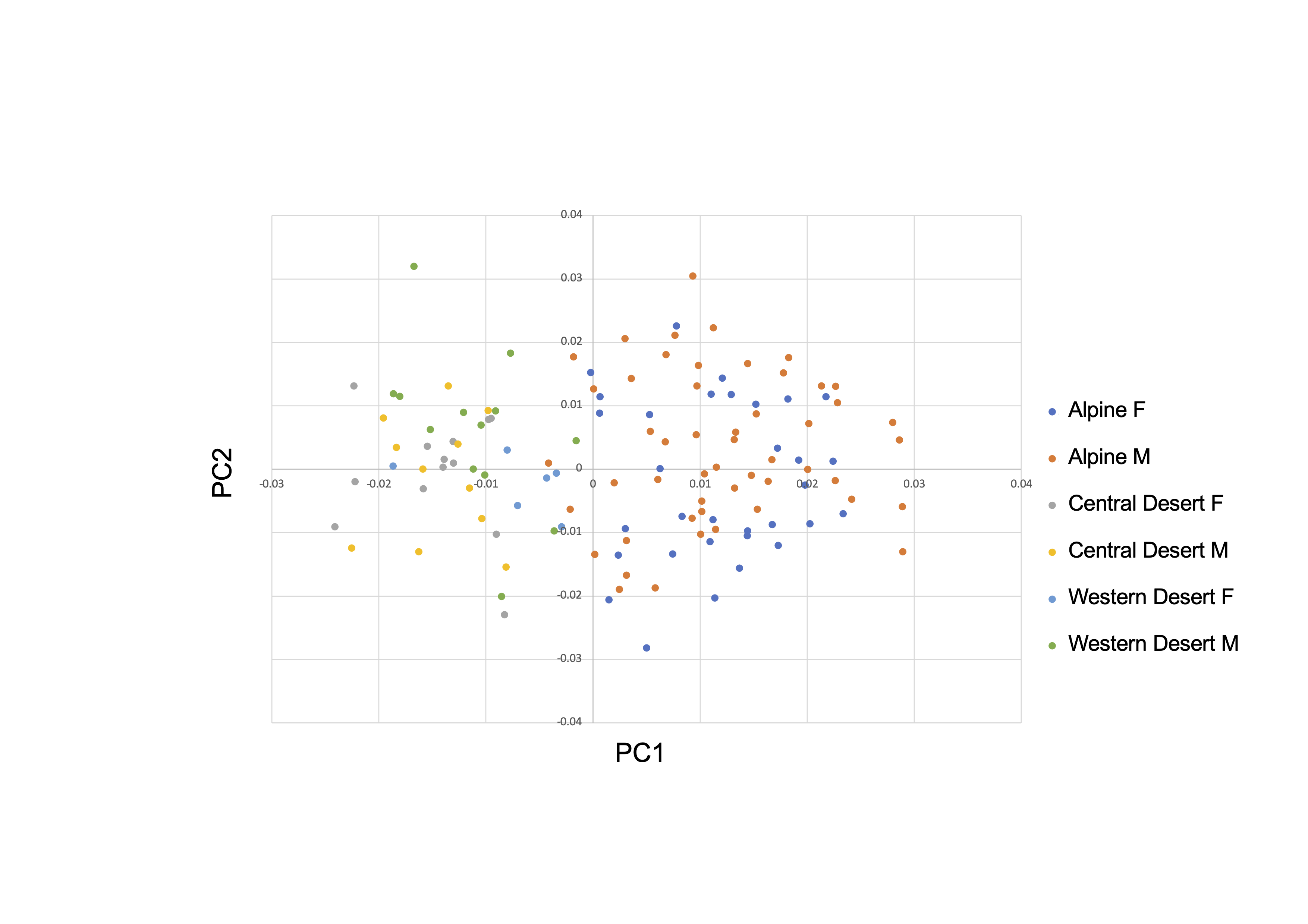

Supplement: giad018_Supplemental_Figures [file giad018_supplemental_figures.zip › Suppl Figure 9.tiff]
