# Supplementary material for: The Australasian dingo archetype: de novo chromosome-length genome assembly, DNA methylome, and cranial morphology
Source: Gigascience. 2023 Mar 28;12:giad018. doi: 10.1093/gigascience/giad018 (PMC10353722; doi:10.1093/gigascience/giad018)
Supplement: giad018_Response_to_Reviewer_Comments_Original_Submission [file giad018_response_to_reviewer_comments_original_submission.pdf]

Author's Response To Reviewer Comments

Close

Reviewer reports:
Reviewer #1: The article "The Australasian dingo archetype: De novo chromosome-length genome assembly, DNA methylome, and cranial morphology" is well written and interesting study examining the evolutionary relationships between the focal species, the dingo, and related canids (both domestic and wild). This study uses an impressive amount of state-of- the-art genomic data and resources to produce a (chromosome-length) de novo assembly of the dingo genome. The approach for assembling the genome are all adequate. The comparisons of chromosomal structural variation and methylation patterns with another dingo ecotype and other canids show interesting patterns of divergence that are potentially important regions of adaptive differences. I have two major concerns and some minor concerns for this paper.

Major concerns: I believe this paper would be stronger if it contained analytical methods that addressed admixture between dingos and domestic dogs more explicitly. The authors state that admixture between dingos and domestic dogs (Line 453) is one of a few hypotheses that may explain phenotypic differences between the two dingo ecotypes. To evaluate this hypothesis with the genomic data, they rely primarily on phylogenetic analyses to explore the evolutionary relationships between the dingo, wolf, and domestic dog lineages. They show that the dingo lineages are outside of the domestic dog clade and that wolves are outside of the dog/dingo clade. Although it is probably true that dingos are a unique evolutionary lineage, phylogenetic analyses are not the strongest tool for assessing admixture and the contribution of genomic variation from different ancestral source populations. I would recommend using methods that would test admixture hypothesis more explicitly. D-statistic tests (ABBA BABA test) and related tests would seem appropriate for this kind of data and sampling scheme.
REPLY: Here we suggest that inclusion of D-statistic tests is beyond the scope of the present study for three reasons.
1. We do acknowledge there are a multitude of downstream analyses that can be completed using the data presented in this study. D-statistic tests are certainly one. Assessment of the divergence time of the lineages is a second. Both these types of studies are best done with multiple individuals within each population. We plan to collect whole genome data from 12 dingoes of each ecotype and complete these analyses in a subsequent manuscript.
2. It is not the goal of the study to differentiate dingoes from dogs.
3. From a stylistic perspective, we are wary to include these population genetic analyses as it has the potential to shift the balance of the study, which has been carefully considered throughout.

I also have concerns about the interpretations of brain size differences between dogs, dingos, and wolves. Although I am intrigued by the idea that domestication may have driven reductions in brain size and shape variation, I find it hard to not consider natural selection pressures in the case of dingos and wolves. The best scenario for testing the domestication- driven hypothesis would be if dogs, dingos, and wolves evolved in a common environment and domestication practices were the most notable differences between them. However, given that wolves and dingos in Australia evolved with different prey and habitats on different continents, it seems hard to me to not consider environmental adaptations as another important factor in the evolution of brain-size and shape variation.
REPLY: We agree that there may be additional, but difficult to test, factors that could impact brain-size evolution. Although there's a robust amount of data across many domesticates (reviewed in Balcarcel et al. 2022) that document brain-size reductions associated with domestication, you're right that environment is an important consideration for each domestication case. We've now noted this in the Discussion.

Minor concerns:
Introduction: It took me awhile reading deeper into the manuscript to understand what was meant by the name Cooinda. For awhile, I thought it was the name for a dingo subspecies or ecotype. I would suggest

including a brief section in the introduction stating that the genomic and morphological data in this study is based off of a single individual named Cooinda and that there are questions about it's ancestry and placement as one of the dingo ecotypes.
REPLY: Added comments to the Figure 1 title and legend.

Line 172: ")," should be replaced with ")."
REPLY: Sentence replaced.

Line 371: I don't think it is necessary to say "The passing of Cooinda the dingo"
REPLY: Paragraph restructured.

Line 463: more is needed to finish the point of "will illuminate"
REPLY: now says "illuminate genetic history."

Line 494-497: The role of venomous animals as barriers to gene flow is conceptually not clear and is not supported by the citations from what I can readily tell.
REPLY: This is now reworded to improve clarity.

Line 540: dewclaws?
REPLY: Corrected

Line 541: "Regrettably" isn't necessary to include
REPLY: Removed

Reviewer #2: My evaluation of the manuscript was restricted to the geometric morphometrics (GM) section. The authors seem to have followed a standard GM procedure in their analysis of cranial shape differences among dingo skull samples. My only suggestion is that additional detail be provided in the landmark data collection for the GM analyses:
REPLY: Added as Supplementary Table 4.

Reference 58 was cited as the source of the landmarks used in this study, but no other details are provided. A list of landmarks that forms the basis of the geometric morphometric analyses should be presented in order for the reader to fully interpret the PCA plots.
REPLY: This has been now addressed in the supplementary info (Supplementary Figure 11a, and Supplementary Table 4.)

Reviewer #3: The manuscript " The Australasian dingo archetype: De novo chromosome- length genome assembly, DNA methylome, and cranial morphology" does describe a de novo genome assembly of the Alpine dingo based on PacBio, ONT, 10X Genomics Chromium, BioNano, and Hi-C. Furthermore, it describes cranial morphometrics and methylation patterns to describe an Alpine dingo "archetype". The methods used seemed overall sound, yet, the writing was often confusing and unclear, so it was difficult to understand what was done and why. The writing, in general, is my biggest criticism of the manuscript, so much so that I was wondering if the authors, by accident, uploaded an earlier version of it. Throughout the manuscript, multiple writing styles and skill levels are evident, and I am sorry to say that it seemed as if the manuscript was copied together from different sources written by the different co-authors rather than a coherent manuscript. Some of the Figure Captions have superscript numbers to highlight individuals and at the same time proper labels that make them obsolete. I first thought they were remnants of footnotes in a previous version. Unfortunately, the methods section, for me one of the most important sections, needs serious improvements. I am not a big fan of having the methods at the end of the manuscript (I know that is how GIGAScience likes it), especially when some methodology is mentioned in the results in a way that you must look up the details in the methods section to understand it. Unfortunately, that is the case with this manuscript.
As there are so many paragraphs in this manuscript that need some improvements, I can only focus on some of them in this review but encourage the authors to have a careful look at the whole manuscript before resubmission, as in the current state, I would not recommend it for publication.
REPLY: This is a large and interdisciplinary m/s. Certainly different sections were written by different authors. The whole manuscript was then pulled together by JWOB before recirculating. We do appreciate

the comments made and have made an effort to improve readability. We now feel the text of the manuscript is improved.

Detailed Comments:
Abstract:
The abstract is overall too long and needs to be much more concise, e.g., the discussion on taxonomic designation (L75-78) should be part of the discussion section but not the background paragraph of the abstract.
L91 "this female" which female?
REPLY: 75-79 removed.
REPLY: Now says "the dingo Cooinda".

Introduction:
I am missing a short review of the taxonomy of the dingo, mostly with respect to the dog or wolf. I know you do not want to draw taxonomic conclusions from this study, but a short review of what others proposed would be helpful. Also, even though everybody knows what a dog is, scientific taxon names are a requirement in scientific writing and should be added at the first mention of any taxon (e.g., dog, gray wolf, dingo, etc.).
REPLY: Section now added to the Introduction. Introduction of this section necessitated restructuring the Introduction. We think this revised structure works well.
REPLY: Scientific names added.

L113: intermediate in what sense? Morphological, behavioral, ecologically?
REPLY: added "evolutionarily intermediate."

L123: Please explain in more detail why a type specimen is needed, especially, as I would argue, that a population-level genomic, morphological and behavioral study would be better to answer if dingoes are feral dogs or an intermediate form instead of a single individual type specimen.
Added: "because we do not have a single reference point that links the scientific name to a specific specimen [30]."

L139: reevaluation
REPLY: Corrected

L142: this can be more concise, e.g., "Zhang et al. (17) found evidence for a separation of Australian dingoes into a northwestern group and a southeastern group, clustering with New Guinea Singing dogs"
REPLY: Corrected as suggested

L150: remove "?"
REPLY: Corrected as suggested

L151-154: This belongs in the discussion.
REPLY: Corrected as suggested

L153: "... being characterized. However, we suggest ...."
REPLY: Removed as suggested above.

Results:
L161: Please consider changing it to chromosome-scale or chromosome-level genome assembly, which is much more common.
REPY: We used this terminology to distinguish it from the mitochondrial genome. However, have now changed the title following some debate.

L162-170: This whole paragraph is a short summary of the methods and does not include a single result. I know it sometimes is nice to recap the methods but in this case I do not see the need for it. Or at least it can be shortened even more to something like: "The final assembly after hybrid long-read assembly, polishing, and scaffolding has a total length of 2,398,209,015 bp ...."

REPLY: Here we disagree with the Reviewer. In the GigaScience format we suggest that an overall summary is very useful at the beginning. In the re-write of this paragraph we do include the Results of the final round of polishing.

L163: I'll highlight it again in the methods, but Supplementary Figure 1 shows 18 pacbio SMRT cells were used, but the methods say 15.
REPLY: 18 SMRT cells.

L164: please be more precise were they pacbio CCS or CLR reads?
REPLY: Clarified.

L167: Please provide Supplementary Figure 2 with a better contrast allowing us to see the high and low contact density on the centre of the scaffold squares.
REPLY: Contrast has now been increased by 25%. You can interactively explore the chromosome-length contact map at multiple resolutions using Juicebox.js (Robinson et al., 2018) following the link given in the legend to Supplementary Figure 2 (https://tinyurl.com/ycbkezf4).

L172: ungapped is not a term I would use; instead, I prefer to refer to the assembly as scaffolded or scaffold-level if it is in scaffolds with gaps and contig-level if the scaffolds are split up into contigs for statistics or analyses. In this case, I would only state the total scaffolded length and maybe the amount of N's or gaps. Also, the second sentence would be better combined with the first e.g., "The final assembly had a total length of 2,398,209,015 bp in 477 scaffolds and a scaffold and contig N50 of 64.8 Mb and 23.1 Mb, respectively." REPLY: Ungapped is an NCBI term and we follow their lead.
REPLY: Sentence corrected as suggested.

L174: What does full-length mean?
REPLY: Changed to Chromosome-level scaffolds

L175: please reference the dog genome properly with the accession number and reference if available.
REPLY: Reference added.

L176: Please rewrite. There is something not quite right with the bracket and the following remaining sentence.
REPLY: Yes, missing a bracket. In response to Reviewer 3 the section has been split into two.
In the Results, simplified to:
Evaluation by Benchmarking Universal Single-Copy Orthologs (BUSCO v5.2.2 [37]) against Carnivora_odb10 data set (n=14,502) indicated that 95.1 % of the conserved single-copy genes were complete (Table 1, Supplementary Fig. 3A). Only 3 of 13,791 complete (single-copy or duplicated) BUSCO genes were not on the 39 nuclear chromosome scaffolds.
In Materials and Methods:
"Assembly completeness was evaluated using BUSCO v5.2.2 [37] short mode against the Carnivora_ob10 data set (n=14,502) implementing BLAST+ v2.11.0 [116], HMMer v3.3 [117], and Metaeuk v20200908 [118]."

L178: Carnivora_odb10
REPLY: Corrected

L182: Please check the manuscript and the supplementary data for consistent spelling of Cooinda (or Cooindah).
REPLY: Cooinda

L184: what does "were full-length by BUSCOMP" mean? Please give more details here on which basis this is determined and what it means that the two other genomes hat a few more. Also, I am not sure if you have to repeat the list with canine assemblies if you have them properly listed in the methods. Again, that's why I prefer to have the methods before the results.
REPLY: Reworded Results: Of the 13,722 genes, 13,711 were found in the Alpine dingo Cooinda assembly using BUSCOMP v1.0.1. Only Sandy the Desert Dingo v2.2 (13,715 genes) and China the Basenji v1.2

(13,712 genes) had more.
REPLY: Reworded Materials and Methods “Complete” BUSCO genes with available sequences were compiled across Alpine dingo Cooinda and nine canid genomes (Desert dingo [6], two Basenji’s (China and Wags) [27], two German shepherd dogs (Nala and Mischa) [28, 36], Great Dane [38], Labrador [39], Dog10K Boxer [40], and Greenland Wolf [41]) using BUSCOMP v1.0.1.
REPLY: We include a list of the assemblies in the Results for ease of reading in the GigaScience format.

Table1: Again "ungapped" sounds not right. Please consider changing it to be clearer. As a general side note, when you want to compare two assemblies of different assembly length, it would be better to compare NG50 instead of N50. I doubt that in this case, with only a 40- 50Mb difference, it would change the results much but consider adding NG50 values. Number of gaps is also not very clear, as the gaps can be of different sizes and can be of a determined length or a standard number of N's as a placeholder for a gap of unknown length.
REPLY: We have followed the standard formatting of NCBI for eukaryotic genome assembles. https://www.ncbi.nlm.nih.gov/assembly/GCA_012295265.2.

L198: "to align Alpine dingo long reads to the Desert dingo assembly" seems not to fit here. Please check the sentence structure and rephrase.
REPLY: Reworded to improve clarity.

L199: "These plots show low variation on the X chromosome" More context is needed. low compared to? Why are the results only so briefly mentioned after multiple lines of "methods". This is an issue I see throughout the results. There are barely any results and mostly method summaries.
REPLY: Added “In comparison to the autosomes,”.
REPLY: We have aimed to keep the manuscript as succinct as possible.
REPLY: This is most generally a function of having the Results before the Materials and Methods. We have tried to balance the manuscript more in the direction that the reviewer considers suitable while maintaining the GigaScience flow.

Figure 2: Explain what the plot shows. I am, in general, not a big fan of these multi-layer circus plots as each individual plot is way too small to show much. However in this case the lower amount of SVs on the X chr is visible enough, but the caption needs more details.
REPLY: We have added “The plot shows the low variation on the X chromosome compared to the autosomes.”

L211: Why list a reference for something that is a results of this study?
REPLY: Reference deleted.

Supplementary Fig. 5: Each chromosome is too small and the resolution too low to see details of the SVs.
REPLY: This is a Supplementary Figure and aimed at giving the reader a general impression of the differences. We expand upon the major SV’s in the text.

L217: So why is that important to mention? If there is no further reason I would remove it, it does not add to the story.
REPLY: These lines expand upon the SV’s (that are difficult to see in Supplementary Fig. 5).

L226: "In addition, however, we also found ..." à change to "In addition, we found... " or "We also found ..."
REPLY: Changed to “We also found”.

L227: Consider joining the two sentences: "We also found two structural events on Chromosome 26 (SFig. 6) containing mostly short genes..."
REPLY: Changed as suggested.

L227: What does perfectly conserved mean?
REPLY: It appears the position of these genes has changed over time. Again, this is important detail and is necessary to mention.

L228-229: Why not show it?
REPLY: MUMMER PLOT added to Supplementary Fig. 6.

L230-232: This can be more concise and easier to read for example: "The Alpine and Desert dingo both have a single copy pancreatic amylase gene (AMY2B) on Chr 6. However, only the copy in the Desert dingo includes a 6.4kb long LINE." I am not sure why reference 10 is cited twice here in the results. Is this a result already known before? If so, this belongs in the discussion.
REPLY: Reworded.

L233: Again, the whole section is a short methodological summary, and there are absolutely no results.
REPLY: This section is included to guide the reader as the Materials and Methods are after the Discussion. The reviewer has stated s/he does not like the format.

Figure 3: The figure caption needs to be rephrased completely. Not sure how this ended up here, but "NOTE: A and C as well as B and D are similar plots. However, A and B use SNVs while C and D use indels." really does not belong in a proper caption, especially as each plot is listed before stating if it is based on SNVs or indels. Bootstrapping usually does not need to be explained in a figure caption. Instead, it would be more important to mention what type of phylogenetic tree it is and on how many SNVs it is based. There is also no scale on the trees, does that mean these are pure cladograms? For B and D, please explain what an ordination analysis is and change the axis labels to something meaningful. Labeling the x and y axis "Axis 1" and "Axis 2" is absolutely pointless. I am quite surprised that this passed the final ok from all co-authors. .
REPLY: Some legend moved to text. Bootstrapping explanation removed and now in Materials and Methods.
REPLY: Added to legend - Branch length proportional to the number of changes and bootstrapping percentage in circles.
REPLY: For B and D we follow the axis labelling convention used in Field et al., (2022). Axis 1 and Axis 2 are similar in concept to PC1 and PC2 which is the classical way to denote Principal Component axis labels (e.g., Supplementary Fig. 9).

L260: please use Desert dingo and not Sandy.
REPLY: Changed to Sandy Desert Dingo to parallel Cooinda Alpine dingo.

L263-265: Not sure why this is important here if it is not discussed later.
REPLY: We include this here as a Result that can be built upon.

Figure 4: Again, the figure caption needs a complete rewrite. For example, L281 "dingo Sandy is in this clade" is very unclear and confusing; "In this figure," à remove!; What are the superscript numbers for? Please remove them, they look like they belong to some footnotes from an earlier manuscript version, which are now missing. Instead, important info such as the type of network, the meaning of the small lines, and the scale are missing.
REPLY: Superscripts deleted. Type of network analyses now included, and the small cross hatched lines defined.

Methylome:
L295: I would remove "the" before MethylSeek and a period is missing before UMRs. REPLY: Corrected as suggested.

L297-299: I think here it would be very nice to not just mention that there are other studies but give some examples and comparisons. "These analyses" could either refer to the MethylSeek analyses or the analyses of reference 55, please rephrase to be clearer. Also, it is unclear what previously reported numbers mean, again give more details ("... in line with previously reported numbers of promoters and enhancers in, e.g., humans (promoters xxxx, enhancers xxxx), mouse (xxxx), and rat (xxxx).")
REPLY: We thank the reviewer for this suggestion. To provide more clarity and give extra details, this sentence now reads as: “MethylSeekR analysis identified ~ 19,000 UMRs and ~44,000 LMRs in line with previously reported numbers of promoters and enhancers (e.g human: ~18,000-20,000 UMRs and 40,000-

70,000 LMRs; mouse: ~17,000-19,000 UMRs and 55,000-90,000 LMRs)"

L301: what does "we lifted over the former UMRs to the latter genome" Please rephrase, it is very unclear to me what you mean.
REPLY: We have now re-phrased the sentence and added extra information to the methods section (page 37). It now reads as: "To establish whether proximal gene regulatory regions in the dingo Cooinda genome display different methylation states in the dingo Sandy genome, we converted Cooinda UMR coordinates from Cooinda to Sandy genome assembly using LiftOver (see Methods)".
Methods (page 37): Cooinda UMR coordinates were converted from Cooinda dingo to Sandy dingo genome assembly using LiftOver following genomewiki.ucsc.edu pipeline (http://genomewiki.ucsc.edu/index.php?title=Minimal_Steps_For_LiftOver). Briefly, the query (Sandy) genome build was split into individual scaffolds using faSplit (i). The we performed pairwise sequence alignment of query sequences from (i) against the Cooinda genome build using BLAT, Then, coordinates of .psl files were changed to parent coordinate system using liftUp and alignments were chained together using axtChain. Chain files were combined and sorted using chainMergeSort; alignment nets were made using chainNet. Finally, liftOver chain file was created using netChainSubset. Cooinda UMRs in .bed format were lifted over to Sandy genome assembly using created liftOver chain file. Average methylation was calculated for Cooinda UMRs and compared to that of corresponding lifted- over regions in Sandy genome. Cooinda UMRs with >50% methylation increase in Sandy's genome were considered as hypermethylated in Sandy's genome.

L302: why was average DNA methylation calculated for UMRs. Should they not be unmethylated by definition?
REPLY: We apologise for the confusion. Indeed, UMRs are hypomethylated by definition, however while the majority of UMRs possess 0-10% mean methylation levels (Figure 1b, Burger et al Nucleic Acids Research 2013), there exists some level of fluctuation of UMR methylation levels (up to 50%, a user-defined cut-off, please see below a paragraph on MethylSeekR parameters). Therefore, in order to determine whether UMRs identified in Cooinda genome possess differential methylation in Sandy genome, we compared average methylation levels of Cooinda UMRs and their corresponding coordinates in Sandy genome (lifted-over regions). Lifted-over regions with >50% methylation gain were deemed hypermethylated in Sandy's genome. We have now added more information to the Methods section (page 37) to clarify the analysis.
MethylSeekR first identifies hypomethylated regions as stretches of CpG sites with methylation levels below a user-defined cut-off meth.cutoff (set to 50% as per original publication Burger et al Nucleic Acids Research 2013, please refer to Methods section MethylC-seq data analysis, page 37: meth.cutoff=0.5) containing a minimal number of CpGs nCpG.cutoff=5. Then, separation of UMRs and LMRs (low-methylated regions, which correspond to distal regulatory regions) is performed based on the number of CpG sites (UMRs are defined as hypomethylated regions with n330 CpG sites) rather than methylation levels.

L306: Why have a sentence about that a gene is highly conserved but not perfect and then give the percentage of identity instead of just stating that it is 99.8% identical? I have now mentioned quite a few examples where the manuscript could be much more concise. I cannot list them all but would encourage you to read through the manuscript again and make it more concise.
REPLY: We thank the reviewer for this suggestion. We have now made it more succinct to read as: "GCGR is on chromosome 9 and has a single transcript. This transcript is 99.8% identical at the amino acid level between the dingoes. HDAC4 occurs on chromosome 25 and has 12 transcripts with all 12 transcripts being 100% identical at the amino acid level."

Morphology:
My knowledge of morphological analyses is limited, but, despite the unfamiliar terminology, this section of the manuscript is easy to read and focuses in a more concise way on the actual results. My only suggestion would be to label Supplementary figure 9a with the different morphological features mentioned or adding an additional schematic to the supplementary, so non-morphologists can easier follow.
REPLY: There is now Supplementary Figure 10b for this purpose. 9a was not really suited to pointing out the features in a visually clear manner.

L353-354: I would suggest adding the sizes after you mention the individual to avoid repeating dingo and dog brain. For example: "... the dingo brain (75.25cm3) was 20% larger than the dog brain (59.53 cm3)

(Figure 5B)."
REPLY: Corrected as suggested.

Figure 5: Please add an explanation of what the polygons in 5A represent. Also, consider changing the labels in 5B. I assume LHS and RHS are short for the left-hand side and the right-hand side. This, for me, is usually used to describe positions in unlabeled figures. I would suggest changing it to Cooinda dingo (CD) and domestic dog (DD).
REPLY: Done. We have re-labelled Fig 5B as suggested using CD and DD.

Discussion
L375: It is not clear why Cooinda should be considered the archetype at the beginning of the discussion. I would place this in the conclusions and base it on the results and the discussion. REPLY: Corrected as suggested.

L394-395: Please rephrase and shorten, e.g.,: "There is a single copy of AMY2B in both dingo genomes; however, they differ by a 6.3 kb retrotransposon insertion present in the Desert dingo."
REPLY: Corrected as suggested.

L394-405: I would like to see a more in-depth discussion on the differences between wolf, dingo, and dog. If there is no LINE in the wolf but both in the dingo and the dogs, when did the transposition happen? Could be two independent events in the dog and dingo lineages or one in the ancestral lineage. Are the LINEs in dog and dingo at the same position in the gene region? Could it be the same insertion that was reduced in length in the dog lineage, and what does that mean for the evolution of dogs and dingoes?
REPLY: Added, As the retrotransposon is absent in the Greenland wolf and Alpine dingo (and they are in different positions) it would seem likely that the retrotransposon has inserted into the Desert dingo and domestic dog lineages independently.

L431/432: please use Alpine and Desert dingo instead of the individuals' names.
REPLY: We have changed Sandy to Desert dingo, but retain Cooinda for consistency.

L471-473: Not sure if a single sample (Cooinda) is sufficient to come to this conclusion, also how does it compare to the wolf? She could just have been a dingo with an exceptionally large brain. I think a more in-depth discussion is needed.
REPLY: added – “An alternative hypothesis is that differences in brain size is due to environmental adaptation or perhaps Cooinda was an anomaly.”

Methods:
Overall, the methods need to be more concise but at the same time clear and complete. L530-531: Why is solving the puzzle-box experiment important? Does that not suggest an exceptionally intelligent dingo if she was the only one, and could that not potentially explain the large brain size? How does brain size and intelligence or the potential to solve the puzzle- box correlate?
REPLY: Phrase concerning puzzle box deleted.

L532: her brothers
REPLY: Corrected as suggested.
L533: What is the importance of the ginger color? As it is stated here, it is a bit out of context. Why is it important?.
REPLY: Here we are giving a context to Cooinda, Not all dingoes are ginger so a future study looking at coat colour will be interested in this detail.

L535-542: Why is this detailed report on her appearance of importance? I am often missing logical connections in the manuscript.
REPLY: As we are attempting to suggest Cooinda become the archetype morphological descriptions are important.

L541: I would usually not expect to read such a statement with an emotional connotation in a scientific manuscript.

REPLY: Now says “Cooinda died in 2019 at 10 years of age.”

L545ff: When were the samples taken? What type of samples were taken? How were they preserved? As it is stated that fresh blood was used, I assume Cooinda was still alive at that point. Are there any sampling and ethics permits to be mentioned?
REPLY: As stated, DNA was extracted from fresh blood. An ethics approval statement is included before competing interests.

L552-56. This whole section about the pulse-field electrophoresis can be much shorter without losing any information, e.g., "Molecular integrity was assessed by pulse-field gel- electrophoresis using the PippinPulse (Sage Science) with a 0.75% KBB gel, Invitrogen 1kb Extension DNA ladder (cat ....) and 150 ng of DNA on the 9hr 10-48kb (80V) program."
REPLY: Modified as suggested.

L556: What libraries? You have not explained how the libraries were prepared. CLR or CCS?
REPLY: Included “SMRTbell libraries with 20kb insert size were CLR sequenced on Sequel I machines with 2.0 chemistry.”

L557: Which Sequel platform was used? Sequel I, II or, IIe?
REPLY: As above, SMRTbell libraries with 20kb insert size were CLR sequenced on Sequel I machines with 2.0 chemistry.

L558: remove the hours of movies, that does not matter unless you used a custom sequencing program.
REPLY: OK

L559: AS 15 SMRT cells were used, I assume the sequencing was performed on the Sequel I.
REPLY: OK

L561: I usually avoid starting a section or paragraph with "for". Please consider rephrasing as you start most paragraphs that way.
REPLY: Generally, we agree. In this case, we have done this intentionally so that the method is right at the front of the sentence. However, it does look a little repetitive so we have modified slightly.

L564: 119 ng of library, especially for long DNA-molecules, seems very low for a decent ONT run. I have mostly used the MinION, and I would usually only load a library with so little DNA if I only needed a few reads. I am just curious how well that worked on the larger PromethION flow cell.
REPLY: Thank you. This protocol was optimised at the Garvan in Sydney.

L573: In some sections, this manuscript reads like an early draft that was accidentally submitted.
"User Guide, manual part number CG00043 Rev B." Please rephrase.
REPLY: (Document # CG00043 revision B).

L575: For me personally, it does not matter where Qubit measurements were taken, but if you include that info, please try not to repeat it as you did in L578.
REPLY: Multiple reference to the Kinghorn centre deleted.

L576-577: Please shorten the two sentences about sequencing to one, e.g., "Sequencing was performed in 150bp paired-end sequencing mode on a single lane on the Illumina HiSeq X Ten platform with a version 2 patterned flowcell."
REPLY: Corrected as suggested.

L581-582: Does reference 8 use the same protocol version? If so, I would remove the brackets. If not, Is there no version number of the protocol available?
REPLY: Corrected as suggested (section in brackets removed).

L594-601: Why is this a mixture of insufficiently described methods and results? Please give additional information about trimming and assembly using canu. Why mention the number of sequences, bubbles,

and unassembled sequences in the methods?
REPLY: The canu parameters used for assembly have been added. Details about the canu assembly stages (correction/trimming/assembly) can be found in the canu manuscript referenced. Details about the number of sequences have been moved to results.

L597: How were the reads aligned to the assembly? I have not used Arrow but if the pipeline uses mapping tools, please mention them.
REPLY: Additional details about the Arrow pipeline have been added as suggested.

L598-601: These are results and should not be part of the methods.
REPLY: Moved as suggested.

L614: what does finishing mean? In the literature, it is more common to write "manually curation of" or scaffolds were "manually curated" or "manually edited".
REPLY: Corrected as suggested.

L615: Again, these are results. I would not place them in the methods.
REPLY: Moved as suggested.

L621: Was gap-closing performed only once? Were pacbio and ONT reads combined on one iteration of gap-closing? Maybe PBJelly suggests only using it once, but in my experience, gap-closing can be performed iteratively to further improve the contiguity.
REPLY: Only one round of gap filling is suggested by the PBJelly documentation. PBJelly was run with the combined alignments of ONT and pacbio read.

L622-623: Again, results in the methods.
REPLY: Moved as suggested.

L634: Why is it important when the chromosome mapping was completed? You did not specify when the sequencing was performed or when the samples were taken.
REPLY: We suggest that this is important as it underpins why we mapped to CanFam3.1 and not 4.1.

L635: Please add accession number and, if available the reference.
REPLY: Accession number = GCF_000002285.3.
Citation: Hoeppner MP, Lundquist A, Pirun M, Meadows JRS, Zamani N, Johnson J, et al. (2014) An Improved Canine Genome and a Comprehensive Catalogue of Coding Genes and Non-Coding Transcripts. PLoS ONE 9(3):
e91172. https://doi.org/10.1371/journal.pone.0091172

L644: Circos is a tool for plotting data in a circular plot. How were the SNV, and indels identified?
REPLY: Added - SNV and indel numbers were calculated using MUMmer4 'show-snp' script following pairwise alignments [37] (v4.0.0 beta 2).

L647: X chromosome
REPLY: Corrected as suggested.

L652: I usually use GeMoMo for homology-based gene prediction. I would like to see a short description of the method rather than [43] just linking to a previous publication.
REPLY: currently: We also used [41] GeMoMa to further investigate whole chromosomal events. Here we mapped genes onto the Alpine Dingo assembly following previously described protocols [8] and then determined the gene order.
new: We also used [41] GeMoMa to further investigate whole chromosomal events. Here we mapped genes onto the Alpine Dingo assembly following previously described protocols [8]. Subsequently, we checked the syntheny of the genes in the reference genome and the target genome using the module GeMoMa module SynthenyChecker. This module uses the GeMoMa annotation with information for reference gene and alternative to determine the best homolog of each transcript. Comparing the order of genes in the reference and the target genome, it allows to determine breakpoints of chromosomal events.

L658: "processes that produce differences" is not very precise, please give some more info here. I would usually remove Indels from phylogenetic datasets due to the uncertainty of their mutational history. How were they coded and how were they analysed?
REPLY: We now simply say "SNV's and indels were analysed separately"
REPLY: Different people have different approaches, so we analyzed both SNP's and indels, but kept them separate.
REPLY: Methods of analyses are presented in the two following paragraphs.

L659: What is WA distance? Reference?
REPLY: Its simply called WA distance. Reference 43 added.

L660: The Glazko et al reference is quite out of context here. Better phylogenetic properties than what?
REPLY: than other coefficients.

Why use distance-based phylogeny? How many SNVs were used? How were they filtered
REPLY: We used distance-based methods so the data could be analysed within weeks. REPLY: SNV's presented in Supplementary Table 2. As described, they were calculated using MUMmer4 'show-snp' script following pairwise aligments.

L662: Maximum parsimony is not frequently used anymore for phylogenetic reconstruction. Why not use a Maximum-likelihood or Bayesian approach?
REPLY: We used both distance and parsimony based methods. No method is perfect.

L664: It is mentioned that the wolf should be the outgroup, but the dataset itself is not mentioned in the methods. List all samples that were included in the phylogeny. If the dingo is assumed to be an intermediate between wolf and dog why did you not use a different canine as outgroup to avoid bias?
REPLY: We used all de novo canine genomes currently available.

L665: include version and the URL of the tool if there is no paper to cite.
REPLY: (github.com/lmc2179/bayesian_bootstrap)

I cannot judge the methods for Methylation and Morphology, as this is not my expertise, but these method sections read very well and seem clear to me.
REPLY: Thank you.

Availability of supporting data:
There are quite some broken sentences and misplaced periods in this section. Please check the text again and make sure that the links to your datasets are functioning.
REPLY: Figshare morphology datasets are live, and the links have been tested – please see:
https://figshare.com/articles/dataset/Dicom_data_MRI_Alpine_dingo_and_domestic_dog_bra in/20514693
https://figshare.com/articles/dataset/Cooinda_Alpine_Dingo_3D_Cranial_Landmarks/205238 04

Overall, the presented data are interesting and a valuable resource, but the manuscript itself needs some major improvements to make the interesting results available to the reader in an easier-to-follow and more understandable form. It is obvious that multiple authors with different expertise worked on different sections of the manuscript. The challenge during the revision is to bring it together into a concise and easy-to-read manuscript with a consistent writing style. I hope that my comments, questions, and suggestions can help in that process. Please take my writing suggestions as such, feel free to adjust and change it in a different way as long as the result is more reader-friendly and more concise.
REPLY: Thank you. By-and-large we followed the Reviewers suggestions. We have also made several changes to improve readability. The comments of the four reviewers are acknowledged.

Reviewer #4: This well written study integrates different approaches and methodologies to tackle the still obscure nature and origin of the dingo and its sub-populations by thoroughly characterising and comparing an "archetype" dingo specimen.
I have read and commented on the abstract and the introduction, as well as the morphology related parts

of the methods, the results and the discussion.
The methods of morphological comparison, as well as their description and the reporting of the results are sound. However, in some sections it is difficult to comprehend the results and their interpretations, as well as the significance and nature of the suggested "archetype" specimen Cooinda. I therefore made some suggestions for additions and edits to the text and the figures, which hopefully help to increase comprehensibility and consistence of the text (see my comments below).
I could not check and comment on the raw data because the links to the supplement given in the manuscript (figshare) do not work. Sorry if I'm stating the obvious here, but to be able to access the raw data is particularly important if the described dingo should act as a reference archetype.

L. 74: Add «of the dingo» after "ecotypes": "[...] compare the Alpine and Desert ecotypes of the dingo [...]". Otherwise it's not really clear what this is about.
REPLY: Corrected as suggested.

L. 91: It's unclear to me what you mean by "this female". I would suggest to exchange this expression with the previously used name of the animal.
REPLY: replaced with "the dingo Cooinda".

L. 94 ff.: The conclusions do not really fit to the rest of the abstract, specifically the aims as stated in the beginning. What I read from the "Background" section is that this work is about defining a "dingo archetype" via different approaches (genetic and morphological). The conclusion, however, is centred around the individual Cooinda. I would suggest to open up this section, to also make conclusions concerning the previously stated aims of the paper.
REPLY: Corrected to follow Reviewer 3.

L. 105 ff. and L. 369 and L. 508: A very nice opening! However, I feel that there is a somewhat misleading interpretation of the domestication process as a discrete trichotomy: wild > tamed > domesticated, when in fact domestication is a continuum with various stages in between the two extremes of the "wild" and the "intensively bred". There are various forms - even today - of "half-domesticated" populations, such as e.g., many of the Asian domestic bovids, or the reindeer. Thus, I would strongly argue that the dingo - although special due to the almost complete lack of human influence on its evolution in the last millennia - is not the only link between the "wild" and the "domesticated". See e.g.: Vigne, Jean-Denis. "The origins of animal domestication and husbandry: a major change in the history of humanity and the biosphere." Comptes rendus biologies 334.3 (2011): 171-181
REPLY: Yes, agreed, we have added further acknowledgment of the alternative hypothesis that domestication represents an intensification of the relationship between humans and animal/plant and have cited Vigne (2011) now in the introduction and discussion in relation to this comment. Lines 113, 385, 535.

L. 117: How do you define "large carnivore"? And: Are dogs more numerous than cats? I don't know the tallies overall, but in many parts of the world domestic cats are more frequent than dogs.
REPLY: To avoid confusion we've deleted 'large carnivore'

L. 120 - 121: I think this sentence does not contribute to the manuscript and I would suggest to delete it. I also think that these are not the usual characteristics to discern the wolf from other canids.
REPLY: Corrected as suggested.

L123 - 125: I do not understand this distinction. In my opinion, the dingo could well be both, a tamed intermediate between wolf and domestic dog AND a feral canid. If I understand the current view of dingo evolution correctly, the dingo most probably constitutes an early domestic stage of the dog, which became feral.
REPLY: We attribute these terms following their use by Darwin. We now say "One alternate hypothesis is that the process of domestication is continual and does not proceed in a stepwise manner [4]."

L. 150: I do not understand the reference to Figure 1 at this point. If you want to keep the figure reference at this place, I would recommend to extend the legend in order to be more descriptive about the significance of this individual dingo. Also: Is the question mark on purpose?
REPLY: Legend to figure 1 now expanded.

Intro and Results in general: Cooinda is central for the research question and the paper. However, I do not really understand her position and significance right away from the text. Maybe this is just a matter of sequence of the paragraphs (some information is given at the beginning of the methods section at the end of the manuscript), but I think it would be crucial to introduce and explain Cooinda and her role (as kind of a reference "archetype") for the aims thoroughly already early on, preferably in the Introduction. This would e.g. also include: why of all the dingoes in Australia is Cooinda an appropriate choice to function as the "archetype". Further, it would be helpful to maybe have a figure showing the geographical distribution of the compared populations (alpine and desert, as well as Cooindas origin) to better understand the setting.
REPLY: We suggest the short expansion of the Legend covers the majority of these points. The selection of Cooinda is presented in the Materials and Methods.

L. 320 ff. and Figure 5: Would it be possible to add a visualisation of the shape changes described in the text into the figure? It is otherwise impossible to evaluate these shape changes.
REPLY: Added – in Supplementary Figure 10.

L. 328 - 345: It would be interesting to pursue the variation along PC2 further: Do you maybe have information from the raw-data if specimens of both the alpine and the desert group that were found to have particularly low or high values for PC2 are especially young and female, or old and male? In other words, do you find evidence in the dataset that there is an actual age and/or sex gradient along PC2? And what age was Cooinda when she died?
REPLY: Thanks, we've added an additional supplementary figure and table to show sex- related trends in PC space, and have tabulated PC2 scores by sex and population. Please see new supplementary figure 11. Data on sex were only available for most of the alpine sample, some of the central desert and a small number of western desert dingoes. Despite a lot of overlap, PC2 scores tends to be lower in females than males in the alpine population and in the western desert. Although central desert males seem to have slightly larger centroid size on average, they score a lower PC2 than females. This is a very gracile population where sexually dimorphic differences are at their most minimal as far as dingoes are considered (Koungoulos 2022:138). We've added additional text to line 350-354 directing readers to the supplementary supporting information. All sampled dingoes were adults ≥ 1 y.o., but no further specificity regarding age was available (pulp-cavity X-rays are generally required for this purpose. As such the role of age in PC2 if any cannot be tested with the present dataset, though it could be (for example) that the male samples include more younger individuals than the female samples. Cooinda as an older individual certainly scores low in PC2.
REPLY: Cooinda was 10 when she died (now added to text).

L. 347: As also pointed out below, it would be important to note somewhere if these two specimens died at about the same time and/or were similarly treated (because of brain shrinkage in specimens that were frozen or otherwise fixed for a long time).
REPLY: Added to the Materials and Methods that "These animals died within 2 weeks of each other."

L. 472: I would suggest to rewrite as: "Cooinda's brain was 20% larger than that of a similarity sized domestic dog [...]". Further, I do not agree with the rest of the statement in this sentence. One of the hallmark characteristics of domestication is brain size reduction, which might be the result of selection for tameness (which you also describe later on). However, selection for tameness (an evolutionary process within a population) is not the same as taming (on the level of the individual). I would therefore suggest to re-write this sentence.
REPLY: Now says: Cooinda's brain was 20% larger than the domestic dog, which is consistent with the hypothesis that she was tamed but not domesticated [3].

Further and in general concerning the brain size part of this study: It would greatly increase the significance of this part of the work if you would compare the dingo brain size not only to one domestic dog, but set it into a larger context. There are plenty of published references for wolf, domestic dog, and dingo brain size estimates and it would be enlightening to compare your findings with those. Of course, there are methodological issues, but maybe a meaningful comparison is possible for some of them. For this I could recommend this review article: Balcarcel, A. M., et al. "The mammalian brain under domestication:

Discovering patterns after a century of old and new analyses." Journal of Experimental Zoology Part B: Molecular and Developmental Evolution (2021).
REPLY: thanks, we've added direct comparison of our brain size data to previous published data on wolf-domestic dog brain size comparisons and we have also cited Balcarcel et al. Please see lines 490-496.

L. 483: Many of the surviving populations of re-introduced (i.e., feral) domestics were part of a fauna that did not correspond to the one of their wild relatives, but was somehow characterised by reduced predation or competition. This was certainly the case for the dingo (few other large predators in Australia) and for some island populations. Maybe you should double-check if this is really the case for the provided examples, but maybe it would be better to write that brain size reduction persists in feral populations at least under certain circumstances.
REPLY: Corrected as suggested.

L. 527: Why is it important that the reference dingo is a female? Please explain.
REPLY: It is not really so we changed to "individual". It does make sequencing simpler as there is no "Y" chromosome to assemble.

L. 535 ff.: Please explain the significance of these special characteristics. Why and how are they special and important for the current study? Also: I'm not a native speaker, but I have the impression that some of the sentences in this section are a bit unusual. Please double-check the grammar.
REPLY: These sentences give a morphological description of Cooinda as a frame of reference.

L. 739: What do you mean by "below" in the brackets?
REPLY: Removed "(below)".

L. 741: Is this the right figure reference? I do not find this figure. Do you mean supplementary Figure 9a?
REPLY: Apologies. Yes, Figure S9a

744 - 745: Could you briefly explain in one sentence the nature and number etc. of landmarks used in this reference study? (For those who cannot check the referenced work.) This would be quite important to be able to interpret the results.
REPLY: A sentence has been added to address this.

L. 744: Delete "earlier".
REPLY: Removed

L. 755: Could you briefly explain here if these were freshly dead specimens, or if they were already older (e.g. frozen, stored in a liquid etc.) please? This has some implications on brain morphology and size.
REPLY: Agreed, thanks. Brains were imaged freshly after the animal died in each case, so they were not stored for long periods of time in fixative. Added "These animals died within 2 weeks of each other".

L. 784 ff.: The figshare-links don't work.
REPLY: Thanks, both Figshare links were live since article submission, we're not sure why this didn't seem to work, please see:
https://figshare.com/articles/dataset/Dicom_data_MRI_Alpine_dingo_and_domestic_dog_bra in/20514693
https://figshare.com/articles/dataset/Cooinda_Alpine_Dingo_3D_Cranial_Landmarks/205238 04

L. 884: I would suggest to re-write the sentence like this: "This was required because the brain was removed immediately after death, which caused some damage to the braincase." Supplementary Figure 9c: It's hard to match the reds of the convex hulls with the reds of the legend. Would it be possible to write down the names right next to the corresponding convex hulls?
REPLY: Corrected as suggested (manuscript text changed as requested and convex hulls labels added to BOTH the regular form PCA in the main text and in the size-adjusted PCA in the supplementary material.)
L. 895: Position remains the same relative to which other analysis? Maybe make a reference to text and/or figure (I guess Fig. 5) here.
REPLY: Corrected as suggested. The reference is to Fig. 5a.

Close
