# Supplementary material for: The Australasian dingo archetype: de novo chromosome-length genome assembly, DNA methylome, and cranial morphology
Source: Gigascience. 2023 Mar 28;12:giad018. doi: 10.1093/gigascience/giad018 (PMC10353722; doi:10.1093/gigascience/giad018)
Supplement: giad018_Response_to_Reviewer_Comments_Revision_1 [file giad018_response_to_reviewer_comments_revision_1.pdf]

Author's Response To Reviewer Comments

Close

Responses to Reviewer

Reviewer reports:
Reviewer #1: The authors have addressed my concerns. I still think it would be valuable to perform admixture tests, but I understand the author's argument that they are saving these analyses for future work.
REPLY: Thank you

Reviewer #3: I would like to congratulate the authors to the greatly improved manuscript and thank them for the detailed responses to my previous comments and suggestions.
REPLY: Thank you

There are only some minor changes that I would suggest to the authors:

The Supplementary Figures are often very small or have labels that are not displayed properly.

I would therefore ask the authors to:

- remove or adjust the labels in Figure S2
REPLY: Removed

- increase the size of Figure S3 (especially S3A) as there are no size constrains in the supplementary material you can fill the page is necessary.
REPLY: Figure will become more visible with increased resolution and ability to explode TIFF files. But tried to fill page.

- increase the size of Figure S6 (mostly the MUMmer plot) and try to improve readability of the labels
REPLY: Corrected

- increase size of Figure S7. Details cannot be seen in C and D
REPLY: Figure will become more visible with increased resolution and ability to explode TIFF files. But tried to fill page.

- increase size of Figure S8 (mostly S8 E)
REPLY: Figure will become more visible with increased resolution and ability to explode TIFF files. But tried to fill page.

- In Figure 4 the red circle indicating the position of Cooinda is not properly centered over the original black circle.
REPLY: Corrected

There are also some minor writing suggestions:

L151- 152: I am missing an article (a/the) before "high resolution ... assembly", and "mitochondrial DNA"
REPLY: Added "plus"

L153:remove one "data" from "computed tomography data from brain data" --> "computed tomography data of the brain" or "computed brain tomography data"
REPLY: Corrected

L173/175 and throughout the manuscript: It seems to me to be more common to refer to SMRT sequence data as PacBio data and in this case PacBio CLR data as it could otherwise the confused with PacBio CCS (HIFI) data.
REPLY: Corrected as suggested throughout manuscript.

L849: "at GCA_012295265.2" The assembly accession is not a location. I would suggest changing it to "is available at NCBI GenBank under the Accession Number GCA_012295265.2" or similar
Is the read data also publicly available? Please add accessions for the data.
REPLY: Added to the availability of supporting material "The read data is available upon request.”.

I would have liked to suggest acceptance of the manuscript but the missing information on the data availability statement did not allow for that as I cannot accept any manuscript without publicly available data.
However, I am happy for the editor to accept the manuscript if the missing information is added.
REPLY: Thank you

Close
