# Supplementary material for: The Australasian dingo archetype: de novo chromosome-length genome assembly, DNA methylome, and cranial morphology
Source: Gigascience. 2023 Mar 28;12:giad018. doi: 10.1093/gigascience/giad018 (PMC10353722; doi:10.1093/gigascience/giad018)
Supplement: giad018_Reviewer_1_Report_Original_Submission — Andreas Chavez -- 12/2/2022 Reviewed [file giad018_reviewer_1_report_original_submission.pdf]

Reviewer Report

Title: The Australasian dingo archetype: De novo chromosome-length genome assembly, DNA methylome, and cranial morphology

Version: Original Submission **Date:** 12/2/2022

Reviewer name: Andreas Chavez

Reviewer Comments to Author:

The article "The Australasian dingo archetype: De novo chromosome-length genome assembly, DNA methylome, and cranial morphology" is well written and interesting study examining the evolutionary relationships between the focal species, the dingo, and related canids (both domestic and wild). This study uses an impressive amount of state-of-the-art genomic data and resources to produce a (chromosome-length) de novo assembly of the dingo genome. The approach for assembling the genome are all adequate. The comparisons of chromosomal structural variation and methylation patterns with another dingo ecotype and other canids show interesting patterns of divergence that are potentially important regions of adaptive differences. I have two major concerns and some minor concerns for this paper.

Major concerns: I believe this paper would be stronger if it contained analytical methods that addressed admixture between dingos and domestic dogs more explicitly. The authors state that admixture between dingos and domestic dogs (Line 453) is one of a few hypotheses that may explain phenotypic differences between the two dingo ecotypes. To evaluate this hypothesis with the genomic data, they rely primarily on phylogenetic analyses to explore the evolutionary relationships between the dingo, wolf, and domestic dog lineages. They show that the dingo lineages are outside of the domestic dog clade and that wolves are outside of the dog/dingo clade. Although it is probably true that dingos are a unique evolutionary lineage, phylogenetic analyses are not the strongest tool for assessing admixture and the contribution of genomic variation from different ancestral source populations. I would recommend using methods that would test admixture hypothesis more explicitly. D-statistic tests (ABBA BABA test) and related tests would seem appropriate for this kind of data and sampling scheme.

I also have concerns about the interpretations of brain size differences between dogs, dingos, and wolves. Although I am intrigued by the idea that domestication may have driven reductions in brain size and shape variation, I find it hard to not consider natural selection pressures in the case of dingos and wolves. The best scenario for testing the domestication-driven hypothesis would be if dogs, dingos, and wolves evolved in a common environment and domestication practices were the most notable differences between them. However, given that wolves and dingos in Australia evolved with different prey and habitats on different continents, it seems hard to me to not consider environmental adaptations as another important factor in the evolution of brain-size and shape variation.

Minor concerns:

Introduction: It took me awhile reading deeper into the manuscript to understand what was meant by the name Cooinda. For awhile, I thought it was the name for a dingo subspecies or ecotype. I would suggest including a brief section in the introduction stating that the genomic and morphological data in

this study is based off of a single individual named Cooinda and that there are questions about it's ancestry and placement as one of the dingo ecotypes.
Line 172: ")," should be replaced with ")."
Line 371: I don't think it is necessary to say "The passing of Cooinda the dingo"
Line 463: more is needed to finish the point of "will illuminate"
Line 494-497: The role of venomous animals as barriers to gene flow is conceptually not clear and is not supported by the citations from what I can readily tell.
Line 540: dewclaws?
Line 541: "Regrettably" isn't necessary to include

Methods

Are the methods appropriate to the aims of the study, are they well described, and are necessary controls included? Choose an item.

Conclusions

Are the conclusions adequately supported by the data shown? Choose an item.

Reporting Standards

Does the manuscript adhere to the journal's guidelines on [minimum standards of reporting?]() Choose an item.

Choose an item.

Statistics

Are you able to assess all statistics in the manuscript, including the appropriateness of statistical tests used? Choose an item.

Quality of Written English

Please indicate the quality of language in the manuscript: Choose an item.

Declaration of Competing Interests

Please complete a declaration of competing interests, considering the following questions:

- Have you in the past five years received reimbursements, fees, funding, or salary from an organisation that may in any way gain or lose financially from the publication of this manuscript, either now or in the future?
- Do you hold any stocks or shares in an organisation that may in any way gain or lose financially from the publication of this manuscript, either now or in the future?

- Do you hold or are you currently applying for any patents relating to the content of the manuscript?
- Have you received reimbursements, fees, funding, or salary from an organization that holds or has applied for patents relating to the content of the manuscript?
- Do you have any other financial competing interests?
- Do you have any non-financial competing interests in relation to this paper?

If you can answer no to all of the above, write 'I declare that I have no competing interests' below. If your reply is yes to any, please give details below.

I declare that I have no competing interests

I agree to the open peer review policy of the journal. I understand that my name will be included on my report to the authors and, if the manuscript is accepted for publication, my named report including any attachments I upload will be posted on the website along with the authors' responses. I agree for my report to be made available under an Open Access Creative Commons CC-BY license (http://creativecommons.org/licenses/by/4.0/). I understand that any comments which I do not wish to be included in my named report can be included as confidential comments to the editors, which will not be published.

Choose an item.

To further support our reviewers, we have joined with Publons, where you can gain additional credit to further highlight your hard work (see: https://publons.com/journal/530/gigascience). On publication of this paper, your review will be automatically added to Publons, you can then choose whether or not to claim your Publons credit. I understand this statement.

Yes Choose an item.
