# Supplementary material for: The Australasian dingo archetype: de novo chromosome-length genome assembly, DNA methylome, and cranial morphology
Source: Gigascience. 2023 Mar 28;12:giad018. doi: 10.1093/gigascience/giad018 (PMC10353722; doi:10.1093/gigascience/giad018)
Supplement: giad018_Reviewer_3_Report_Original_Submission — Sven Winter -- 12/9/2022 Reviewed [file giad018_reviewer_3_report_original_submission.pdf]

Reviewer Report

Title: The Australasian dingo archetype: De novo chromosome-length genome assembly, DNA methylome, and cranial morphology

Version: Original Submission **Date:** 12/9/2022

Reviewer name: Sven Winter

Reviewer Comments to Author:

The manuscript " The Australasian dingo archetype: De novo chromosome-length genome assembly, DNA methylome, and cranial morphology" does describe a de novo genome assembly of the Alpine dingo based on PacBio, ONT, 10X Genomics Chromium, BioNano, and Hi-C. Furthermore, it describes cranial morphometrics and methylation patterns to describe an Alpine dingo "archetype".
The methods used seemed overall sound, yet, the writing was often confusing and unclear, so it was difficult to understand what was done and why.
The writing, in general, is my biggest criticism of the manuscript, so much so that I was wondering if the authors, by accident, uploaded an earlier version of it.
Throughout the manuscript, multiple writing styles and skill levels are evident, and I am sorry to say that it seemed as if the manuscript was copied together from different sources written by the different co-authors rather than a coherent manuscript. Some of the Figure Captions have superscript numbers to highlight individuals and at the same time proper labels that make them obsolete. I first thought they were remnants of footnotes in a previous version. Unfortunately, the methods section, for me one of the most important sections, needs serious improvements. I am not a big fan of having the methods at the end of the manuscript (I know that is how GIGAScience likes it), especially when some methodology is mentioned in the results in a way that you must look up the details in the methods section to understand it. Unfortunately, that is the case with this manuscript.
As there are so many paragraphs in this manuscript that need some improvements, I can only focus on some of them in this review but encourage the authors to have a careful look at the whole manuscript before resubmission, as in the current state, I would not recommend it for publication.
Detailed Comments:
Abstract:
The abstract is overall too long and needs to be much more concise, e.g., the discussion on taxonomic designation (L75-78) should be part of the discussion section but not the background paragraph of the abstract.
L91 "this female" which female?
Introduction:
I am missing a short review of the taxonomy of the dingo, mostly with respect to the dog or wolf. I know you do not want to draw taxonomic conclusions from this study, but a short review of what others proposed would be helpful. Also, even though everybody knows what a dog is, scientific taxon names are a requirement in scientific writing and should be added at the first mention of any taxon (e.g., dog, gray wolf, dingo, etc.).

L113: intermediate in what sense? Morphological, behavioral, ecologically?
L123: Please explain in more detail why a type specimen is needed, especially, as I would argue, that a population-level genomic, morphological and behavioral study would be better to answer if dingoes are feral dogs or an intermediate form instead of a single individual type specimen.
L139: reevaluation
L142: this can be more concise, e.g., "Zhang et al. (17) found evidence for a separation of Australian dingoes into a northwestern group and a southeastern group, clustering with New Guinea Singing dogs"
L150: remove "?"
L151-154: This belongs in the discussion.
L153: "… being characterized. However, we suggest …."
Results:
L161: Please consider changing it to chromosome-scale or chromosome-level genome assembly, which is much more common.
L162-170: This whole paragraph is a short summary of the methods and does not include a single result. I know it sometimes is nice to recap the methods but in this case I do not see the need for it. Or at least it can be shortened even more to something like: "The final assembly after hybrid long-read assembly, polishing, and scaffolding has a total length of 2,398,209,015 bp …."
L163: I'll highlight it again in the methods, but Supplementary Figure 1 shows 18 pacbio SMRT cells were used, but the methods say 15.
L164: please be more precise were they pacbio CCS or CLR reads?
L167: Please provide Supplementary Figure 2 with a better contrast allowing us to see the high and low contact density on the centre of the scaffold squares.
L172: ungapped is not a term I would use; instead, I prefer to refer to the assembly as scaffolded or scaffold-level if it is in scaffolds with gaps and contig-level if the scaffolds are split up into contigs for statistics or analyses. In this case, I would only state the total scaffolded length and maybe the amount of N's or gaps. Also, the second sentence would be better combined with the first e.g., "The final assembly had a total length of 2,398,209,015 bp in 477 scaffolds and a scaffold and contig N50 of 64.8 Mb and 23.1 Mb, respectively."
L174: What does full-length mean?
L175: please reference the dog genome properly with the accession number and reference if available.
L176: Please rewrite. There is something not quite right with the bracket and the following remaining sentence.
L178: Carnivora_odb10
L182: Please check the manuscript and the supplementary data for consistent spelling of Cooinda (or Cooindah).
L184: what does "were full-length by BUSCOMP" mean? Please give more details here on which basis this is determined and what it means that the two other genomes hat a few more. Also, I am not sure if you have to repeat the list with canine assemblies if you have them properly listed in the methods. Again, that's why I prefer to have the methods before the results.
Table1: Again "ungapped" sounds not right. Please consider changing it to be clearer. As a general side note, when you want to compare two assemblies of different assembly length, it would be better to compare NG50 instead of N50. I doubt that in this case, with only a 40-50Mb difference, it would change

the results much but consider adding NG50 values. Number of gaps is also not very clear, as the gaps can be of different sizes and can be of a determined length or a standard number of N's as a placeholder for a gap of unknown length.
L198: "to align Alpine dingo long reads to the Desert dingo assembly" seems not to fit here. Please check the sentence structure and rephrase.
L199: "These plots show low variation on the X chromosome" More context is needed. low compared to? Why are the results only so briefly mentioned after multiple lines of "methods". This is an issue I see throughout the results. There are barely any results and mostly method summaries.
Figure 2: Explain what the plot shows. I am, in general, not a big fan of these multi-layer circus plots as each individual plot is way too small to show much. However in this case the lower amount of SVs on the X chr is visible enough, but the caption needs more details.
L211: Why list a reference for something that is a results of this study?
Supplementary Fig. 5: Each chromosome is too small and the resolution too low to see details of the SVs.
L217: So why is that important to mention? If there is no further reason I would remove it, it does not add to the story.
L226: "In addition, however, we also found …" ïƒ change to "In addition, we found… " or "We also found …"
L227: Consider joining the two sentences: "We also found two structural events on Chromosome 26 (SFig. 6) containing mostly short genes…"
L227: What does perfectly conserved mean?
L228-229: Why not show it?
L230-232: This can be more concise and easier to read for example: "The Alpine and Desert dingo both have a single copy pancreatic amylase gene (AMY2B) on Chr 6. However, only the copy in the Desert dingo includes a 6.4kb long LINE." I am not sure why reference 10 is cited twice here in the results. Is this a result already known before? If so, this belongs in the discussion.
L233: Again, the whole section is a short methodological summary, and there are absolutely no results.
Figure 3: The figure caption needs to be rephrased completely. Not sure how this ended up here, but "NOTE: A and C as well as B and D are similar plots. However, A and B use SNVs while C and D use indels." really does not belong in a proper caption, especially as each plot is listed before stating if it is based on SNVs or indels. Bootstrapping usually does not need to be explained in a figure caption. Instead, it would be more important to mention what type of phylogenetic tree it is and on how many SNVs it is based. There is also no scale on the trees, does that mean these are pure cladograms? For B and D, please explain what an ordination analysis is and change the axis labels to something meaningful. Labeling the x and y axis "Axis 1" and "Axis 2" is absolutely pointless. I am quite surprised that this passed the final ok from all co-authors.
L260: please use Desert dingo and not Sandy.
L263-265: Not sure why this is important here if it is not discussed later.
Figure 4: Again, the figure caption needs a complete rewrite. For example, L281 "dingo Sandy is in this clade" is very unclear and confusing; "In this figure," ïƒ remove!; What are the superscript numbers for? Please remove them, they look like they belong to some footnotes from an earlier manuscript version, which are now missing. Instead, important info such as the type of network, the meaning of the small

lines, and the scale are missing.
Methylome:
L295: I would remove "the" before MethylSeek and a period is missing before UMRs.
L297-299: I think here it would be very nice to not just mention that there are other studies but give some examples and comparisons. "These analyses" could either refer to the MethylSeek analyses or the analyses of reference 55, please rephrase to be clearer. Also, it is unclear what previously reported numbers mean, again give more details ("… in line with previously reported numbers of promoters and enhancers in, e.g., humans (promoters xxxx, enhancers xxxx), mouse (xxxx), and rat (xxxx).")
L301: what does "we lifted over the former UMRs to the latter genome" Please rephrase, it is very unclear to me what you mean.
L302: why was average DNA methylation calculated for UMRs. Should they not be unmethylated by definition?
L306: Why have a sentence about that a gene is highly conserved but not perfect and then give the percentage of identity instead of just stating that it is 99.8% identical? I have now mentioned quite a few examples where the manuscript could be much more concise. I cannot list them all but would encourage you to read through the manuscript again and make it more concise.
Morphology:
My knowledge of morphological analyses is limited, but, despite the unfamiliar terminology, this section of the manuscript is easy to read and focuses in a more concise way on the actual results. My only suggestion would be to label Supplementary figure 9a with the different morphological features mentioned or adding an additional schematic to the supplementary, so non-morphologists can easier follow.
L353-354: I would suggest adding the sizes after you mention the individual to avoid repeating dingo and dog brain. For example: "… the dingo brain (75.25cm3) was 20% larger than the dog brain (59.53 cm3) (Figure 5B)."
Figure 5: Please add an explanation of what the polygons in 5A represent. Also, consider changing the labels in 5B. I assume LHS and RHS are short for the left-hand side and the right-hand side. This, for me, is usually used to describe positions in unlabeled figures. I would suggest changing it to Cooinda dingo (CD) and domestic dog (DD).
Discussion
L375: It is not clear why Cooinda should be considered the archetype at the beginning of the discussion. I would place this in the conclusions and base it on the results and the discussion.
L394-395: Please rephrase and shorten, e.g.,: "There is a single copy of AMY2B in both dingo genomes; however, they differ by a 6.3 kb retrotransposon insertion present in the Desert dingo."
L394-405: I would like to see a more in-depth discussion on the differences between wolf, dingo, and dog. If there is no LINE in the wolf but both in the dingo and the dogs, when did the transposition happen? Could be two independent events in the dog and dingo lineages or one in the ancestral lineage. Are the LINEs in dog and dingo at the same position in the gene region? Could it be the same insertion that was reduced in length in the dog lineage, and what does that mean for the evolution of dogs and dingoes?
L431/432: please use Alpine and Desert dingo instead of the individuals' names.
L471-473: Not sure if a single sample (Cooinda) is sufficient to come to this conclusion, also how does it

compare to the wolf? She could just have been a dingo with an exceptionally large brain. I think a more in-depth discussion is needed.
Methods:
Overall, the methods need to be more concise but at the same time clear and complete.
L530-531: Why is solving the puzzle-box experiment important? Does that not suggest an exceptionally intelligent dingo if she was the only one, and could that not potentially explain the large brain size? How does brain size and intelligence or the potential to solve the puzzle-box correlate?
L532: her brothers
L533: What is the importance of the ginger color? As it is stated here, it is a bit out of context. Why is it important?
L535-542: Why is this detailed report on her appearance of importance? I am often missing logical connections in the manuscript.
L541: I would usually not expect to read such a statement with an emotional connotation in a scientific manuscript.
L545ff: When were the samples taken? What type of samples were taken? How were they preserved? As it is stated that fresh blood was used, I assume Cooinda was still alive at that point. Are there any sampling and ethics permits to be mentioned?
L552-56. This whole section about the pulse-field electrophoresis can be much shorter without losing any information, e.g., "Molecular integrity was assessed by pulse-field gel-electrophoresis using the PippinPulse (Sage Science) with a 0.75% KBB gel, Invitrogen 1kb Extension DNA ladder (cat ….) and 150 ng of DNA on the 9hr 10-48kb (80V) program."
L556: What libraries? You have not explained how the libraries were prepared. CLR or CCS?
L557: Which Sequel platform was used? Sequel I, II or, IIe?
L558: remove the hours of movies, that does not matter unless you used a custom sequencing program.
L559: AS 15 SMRT cells were used, I assume the sequencing was performed on the Sequel I.
L561: I usually avoid starting a section or paragraph with "for". Please consider rephrasing as you start most paragraphs that way.
L564: 119 ng of library, especially for long DNA-molecules, seems very low for a decent ONT run. I have mostly used the MinION, and I would usually only load a library with so little DNA if I only needed a few reads. I am just curious how well that worked on the larger PromethION flow cell.
L573: In some sections, this manuscript reads like an early draft that was accidentally submitted.
"User Guide, manual part number CG00043 Rev B." Please rephrase.
L575: For me personally, it does not matter where Qubit measurements were taken, but if you include that info, please try not to repeat it as you did in L578.
L576-577: Please shorten the two sentences about sequencing to one, e.g., "Sequencing was performed in 150bp paired-end sequencing mode on a single lane on the Illumina HiSeq X Ten platform with a version 2 patterned flowcell."
L581-582: Does reference 8 use the same protocol version? If so, I would remove the brackets. If not, Is there no version number of the protocol available?
L594-601: Why is this a mixture of insufficiently described methods and results? Please give additional information about trimming and assembly using canu. Why mention the number of sequences, bubbles, and unassembled sequences in the methods?

L597: How were the reads aligned to the assembly? I have not used Arrow but if the pipeline uses mapping tools, please mention them.
L598-601: These are results and should not be part of the methods.
L614: what does finishing mean? In the literature, it is more common to write "manually curation of" or scaffolds were "manually curated" or "manually edited".
L615ff: Again, these are results. I would not place them in the methods.
L621: Was gap-closing performed only once? Were pacbio and ONT reads combined on one iteration of gap-closing? Maybe PBJelly suggests only using it once, but in my experience, gap-closing can be performed iteratively to further improve the contiguity.
L622-623: Again, results in the methods.
L634: Why is it important when the chromosome mapping was completed? You did not specify when the sequencing was performed or when the samples were taken.
L635: Please add accession number and, if available the reference.
L644: Circos is a tool for plotting data in a circular plot. How were the SNV, and indels identified?
L647: X chromosome
L652: I usually use GeMoMo for homology-based gene prediction. I would like to see a short description of the method rather than just linking to a previous publication.
L658: "processes that produce differences" is not very precise, please give some more info here. I would usually remove Indels from phylogenetic datasets due to the uncertainty of their mutational history. How were they coded and how were they analysed?
L659: What is WA distance? Reference?
L660: The Glazko et al reference is quite out of context here. Better phylogenetic properties than what? Why use distance-based phylogeny? How many SNVs were used? How ere they filtered
L662: Maximum parsimony is not frequently used anymore for phylogenetic reconstruction. Why not use a Maximum-likelihood or Bayesian approach?
L664: It is mentioned that the wolf should be the outgroup, but the dataset itself is not mentioned in the methods. List all samples that were included in the phylogeny. If the dingo is assumed to be an intermediate between wolf and dog why did you not use a different canine as outgroup to avoid bias?
L665: include version and the URL of the tool if there is no paper to cite.
I cannot judge the methods for Methylation and Morphology, as this is not my expertise, but these method sections read very well and seem clear to me.
Availability of supporting data:
There are quite some broken sentences and misplaced periods in this section. Please check the text again and make sure that the links to your datasets are functioning.
Overall, the presented data are interesting and a valuable resource, but the manuscript itself needs some major improvements to make the interesting results available to the reader in an easier-to-follow and more understandable form. It is obvious that multiple authors with different expertise worked on different sections of the manuscript. The challenge during the revision is to bring it together into a concise and easy-to-read manuscript with a consistent writing style. I hope that my comments, questions, and suggestions can help in that process. Please take my writing suggestions as such, feel free to adjust and change it in a different way as long as the result is more reader-friendly and more concise.

Methods

Are the methods appropriate to the aims of the study, are they well described, and are necessary controls included? Choose an item.

Conclusions

Are the conclusions adequately supported by the data shown? Choose an item.

Reporting Standards

Does the manuscript adhere to the journal's guidelines on minimum standards of reporting? Choose an item.

Choose an item.

Statistics

Are you able to assess all statistics in the manuscript, including the appropriateness of statistical tests used? Choose an item.

Quality of Written English

Please indicate the quality of language in the manuscript: Choose an item.

Declaration of Competing Interests

Please complete a declaration of competing interests, considering the following questions:

- Have you in the past five years received reimbursements, fees, funding, or salary from an organisation that may in any way gain or lose financially from the publication of this manuscript, either now or in the future?
- Do you hold any stocks or shares in an organisation that may in any way gain or lose financially from the publication of this manuscript, either now or in the future?
- Do you hold or are you currently applying for any patents relating to the content of the manuscript?
- Have you received reimbursements, fees, funding, or salary from an organization that holds or has applied for patents relating to the content of the manuscript?
- Do you have any other financial competing interests?
- Do you have any non-financial competing interests in relation to this paper?

If you can answer no to all of the above, write 'I declare that I have no competing interests' below. If your reply is yes to any, please give details below.

I declare that I have no competing interests

I agree to the open peer review policy of the journal. I understand that my name will be included on my report to the authors and, if the manuscript is accepted for publication, my named report including any attachments I upload will be posted on the website along with the authors' responses. I agree for my report to be made available under an Open Access Creative Commons CC-BY license (http://creativecommons.org/licenses/by/4.0/). I understand that any comments which I do not wish to be included in my named report can be included as confidential comments to the editors, which will not be published.

Choose an item.

To further support our reviewers, we have joined with Publons, where you can gain additional credit to further highlight your hard work (see: https://publons.com/journal/530/gigascience). On publication of this paper, your review will be automatically added to Publons, you can then choose whether or not to claim your Publons credit. I understand this statement.

Yes Choose an item.
