# Supplementary material for: The Australasian dingo archetype: de novo chromosome-length genome assembly, DNA methylome, and cranial morphology
Source: Gigascience. 2023 Mar 28;12:giad018. doi: 10.1093/gigascience/giad018 (PMC10353722; doi:10.1093/gigascience/giad018)
Supplement: giad018_Reviewer_3_Report_Revision_1 — Sven Winter -- 1/19/2023 Reviewed [file giad018_reviewer_3_report_revision_1.pdf]

Reviewer Report

Title: The Australasian dingo archetype: De novo chromosome-length genome assembly, DNA methylome, and cranial morphology

Version: Revision 1 **Date:** 1/19/2023

Reviewer name: Sven Winter

Reviewer Comments to Author:

I would like to congratulate the authors to the greatly improved manuscript and thank them for the detailed responses to my previous comments and suggestions.
There are only some minor changes that I would suggest to the authors:
The Supplementary Figures are often very small or have labels that are not displayed properly.
I would therefore ask the authors to:
- remove or adjust the labels in Figure S2
- increase the size of Figure S3 (especially S3A) as there are no size constrains in the supplementary material you can fill the page is necessary.
- increase the size of Figure S6 (mostly the MUMmer plot) and try to improve readability of the labels
- increase size of Figure S7. Details cannot be seen in C and D
- increase size of Figure S8 (mostly S8 E)
- In Figure 4 the red circle indicating the position of Cooinda is not properly centered over the original black circle.

There are also some minor writing suggestions:
L151- 152: I am missing an article (a/the) before "high resolution ... assembly", and "mitochondrial DNA"
L153:remove one "data" from "computed tomography data from brain data" --> "computed tomography data of the brain" or "computed brain tomography data"
L173/175 and throughout the manuscript: It seems to me to be more common to refer to SMRT sequence data as PacBio data and in this case PacBio CLR data as it could otherwise the confused with PacBio CCS (HIFI) data.
L849: "at GCA_012295265.2" The assembly accession is not a location. I would suggest changing it to "is available at NCBI GenBank under the Accession Number GCA_012295265.2" or similar
Is the read data also publicly available? Please add accessions for the data.
I would have liked to suggest acceptance of the manuscript but the missing information on the data availability statement did not allow for that as I cannot accept any manuscript without publicly available data.
However, I am happy for the editor to accept the manuscript if the missing information is added.

Methods

Are the methods appropriate to the aims of the study, are they well described, and are necessary controls included? Choose an item.

Conclusions

Are the conclusions adequately supported by the data shown? Choose an item.

Reporting Standards

Does the manuscript adhere to the journal's guidelines on minimum standards of reporting? Choose an item.

Choose an item.

Statistics

Are you able to assess all statistics in the manuscript, including the appropriateness of statistical tests used? Choose an item.

Quality of Written English

Please indicate the quality of language in the manuscript: Choose an item.

Declaration of Competing Interests

Please complete a declaration of competing interests, considering the following questions:

- Have you in the past five years received reimbursements, fees, funding, or salary from an organisation that may in any way gain or lose financially from the publication of this manuscript, either now or in the future?
- Do you hold any stocks or shares in an organisation that may in any way gain or lose financially from the publication of this manuscript, either now or in the future?
- Do you hold or are you currently applying for any patents relating to the content of the manuscript?
- Have you received reimbursements, fees, funding, or salary from an organization that holds or has applied for patents relating to the content of the manuscript?
- Do you have any other financial competing interests?
- Do you have any non-financial competing interests in relation to this paper?

If you can answer no to all of the above, write 'I declare that I have no competing interests' below. If your reply is yes to any, please give details below.

I declare that I have no competing interests

I agree to the open peer review policy of the journal. I understand that my name will be included on my report to the authors and, if the manuscript is accepted for publication, my named report including any attachments I upload will be posted on the website along with the authors' responses. I agree for my

report to be made available under an Open Access Creative Commons CC-BY license (http://creativecommons.org/licenses/by/4.0/). I understand that any comments which I do not wish to be included in my named report can be included as confidential comments to the editors, which will not be published.

Choose an item.

To further support our reviewers, we have joined with Publons, where you can gain additional credit to further highlight your hard work (see: https://publons.com/journal/530/gigascience). On publication of this paper, your review will be automatically added to Publons, you can then choose whether or not to claim your Publons credit. I understand this statement.

Yes Choose an item.
