# Supplementary material for: The Australasian dingo archetype: de novo chromosome-length genome assembly, DNA methylome, and cranial morphology
Source: Gigascience. 2023 Mar 28;12:giad018. doi: 10.1093/gigascience/giad018 (PMC10353722; doi:10.1093/gigascience/giad018)
Supplement: giad018_Reviewer_4_Report_Original_Submission — Madeleine Geiger -- 12/14/2022 Reviewed [file giad018_reviewer_4_report_original_submission.pdf]

Reviewer Report

Title: The Australasian dingo archetype: De novo chromosome-length genome assembly, DNA methylome, and cranial morphology

Version: Original Submission **Date:** 12/14/2022

Reviewer name: Madeleine Geiger

Reviewer Comments to Author:

This well written study integrates different approaches and methodologies to tackle the still obscure nature and origin of the dingo and its sub-populations by thoroughly characterising and comparing an "archetype" dingo specimen.
I have read and commented on the abstract and the introduction, as well as the morphology related parts of the methods, the results and the discussion.
The methods of morphological comparison, as well as their description and the reporting of the results are sound. However, in some sections it is difficult to comprehend the results and their interpretations, as well as the significance and nature of the suggested "archetype" specimen Cooinda. I therefore made some suggestions for additions and edits to the text and the figures, which hopefully help to increase comprehensibility and consistence of the text (see my comments below).
I could not check and comment on the raw data because the links to the supplement given in the manuscript (figshare) do not work. Sorry if I'm stating the obvious here, but to be able to access the raw data is particularly important if the described dingo should act as a reference archetype.
L. 74: Add Â«of the dingoÂ» after "ecotypes": "[…] compare the Alpine and Desert ecotypes of the dingo […]". Otherwise it's not really clear what this is about.
L. 91: It's unclear to me what you mean by "this female". I would suggest to exchange this expression with the previously used name of the animal.
L. 94 ff.: The conclusions do not really fit to the rest of the abstract, specifically the aims as stated in the beginning. What I read from the "Background" section is that this work is about defining a "dingo archetype" via different approaches (genetic and morphological). The conclusion, however, is centred around the individual Cooinda. I would suggest to open up this section, to also make conclusions concerning the previously stated aims of the paper.
L. 105 ff. and L. 369 and L. 508: A very nice opening! However, I feel that there is a somewhat misleading interpretation of the domestication process as a discrete trichotomy: wild > tamed > domesticated, when in fact domestication is a continuum with various stages in between the two extremes of the "wild" and the "intensively bred". There are various forms - even today - of "half-domesticated" populations, such as e.g., many of the Asian domestic bovids, or the reindeer. Thus, I would strongly argue that the dingo - although special due to the almost complete lack of human influence on its evolution in the last millennia - is not the only link between the "wild" and the "domesticated". See e.g.: Vigne, Jean-Denis. "The origins of animal domestication and husbandry: a major change in the history of humanity and the biosphere." Comptes rendus biologies 334.3 (2011): 171-181
L. 117: How do you define "large carnivore"? And: Are dogs more numerous than cats? I don't know the

tallies overall, but in many parts of the world domestic cats are more frequent than dogs.
L. 120 - 121: I think this sentence does not contribute to the manuscript and I would suggest to delete it. I also think that these are not the usual characteristics to discern the wolf from other canids.
123 - 125: I do not understand this distinction. In my opinion, the dingo could well be both, a tamed intermediate between wolf and domestic dog AND a feral canid. If I understand the current view of dingo evolution correctly, the dingo most probably constitutes an early domestic stage of the dog, which became feral.
L. 150: I do not understand the reference to Figure 1 at this point. If you want to keep the figure reference at this place, I would recommend to extend the legend in order to be more descriptive about the significance of this individual dingo. Also: Is the question mark on purpose?
Intro and Results in general: Cooinda is central for the research question and the paper. However, I do not really understand her position and significance right away from the text. Maybe this is just a matter of sequence of the paragraphs (some information is given at the beginning of the methods section at the end of the manuscript), but I think it would be crucial to introduce and explain Cooinda and her role (as kind of a reference "archetype") for the aims thoroughly already early on, preferably in the Introduction. This would e.g. also include: why of all the dingoes in Australia is Cooinda an appropriate choice to function as the "archetype". Further, it would be helpful to maybe have a figure showing the geographical distribution of the compared populations (alpine and desert, as well as Cooindas origin) to better understand the setting.
L. 320 ff. and Figure 5: Would it be possible to add a visualisation of the shape changes described in the text into the figure? It is otherwise impossible to evaluate these shape changes.
L. 328 - 345: It would be interesting to pursue the variation along PC2 further: Do you maybe have information from the raw-data if specimens of both the alpine and the desert group that were found to have particularly low or high values for PC2 are especially young and female, or old and male? In other words, do you find evidence in the dataset that there is an actual age and/or sex gradient along PC2? And what age was Cooinda when she died?
L. 347: As also pointed out below, it would be important to note somewhere if these two specimens died at about the same time and/or were similarly treated (because of brain shrinkage in specimens that were frozen or otherwise fixed for a long time).
L. 472: I would suggest to rewrite as: "Cooinda's brain was 20% larger than that of a similarity sized domestic dog […]". Further, I do not agree with the rest of the statement in this sentence. One of the hallmark characteristics of domestication is brain size reduction, which might be the result of selection for tameness (which you also describe later on). However, selection for tameness (an evolutionary process within a population) is not the same as taming (on the level of the individual). I would therefore suggest to re-write this sentence.
Further and in general concerning the brain size part of this study: It would greatly increase the significance of this part of the work if you would compare the dingo brain size not only to one domestic dog, but set it into a larger context. There are plenty of published references for wolf, domestic dog, and dingo brain size estimates and it would be enlightening to compare your findings with those. Of course, there are methodological issues, but maybe a meaningful comparison is possible for some of them. For this I could recommend this review article: Balcarcel, A. M., et al. "The mammalian brain under domestication: Discovering patterns after a century of old and new analyses." Journal of Experimental

Zoology Part B: Molecular and Developmental Evolution (2021).
L. 483: Many of the surviving populations of re-introduced (i.e., feral) domestics were part of a fauna that did not correspond to the one of their wild relatives, but was somehow characterised by reduced predation or competition. This was certainly the case for the dingo (few other large predators in Australia) and for some island populations. Maybe you should double-check if this is really the case for the provided examples, but maybe it would be better to write that brain size reduction persists in feral populations at least under certain circumstances.
L. 527: Why is it important that the reference dingo is a female? Please explain.
L. 535 ff.: Please explain the significance of these special characteristics. Why and how are they special and important for the current study? Also: I'm not a native speaker, but I have the impression that some of the sentences in this section are a bit unusual. Please double-check the grammar.
L. 739: What do you mean by "below" in the brackets?
L. 741: Is this the right figure reference? I do not find this figure. Do you mean supplementary Figure 9a?
744 - 745: Could you briefly explain in one sentence the nature and number etc. of landmarks used in this reference study? (For those who cannot check the referenced work.) This would be quite important to be able to interpret the results.
L. 744: Delete "earlier".
L. 755: Could you briefly explain here if these were freshly dead specimens, or if they were already older (e.g. frozen, stored in a liquid etc.) please? This has some implications on brain morphology and size.
L. 784 ff.: The figshare-links don't work.
L. 884: I would suggest to re-write the sentence like this: "This was required because the brain was removed immediately after death, which caused some damage to the braincase."
Supplementary Figure 9c: It's hard to match the reds of the convex hulls with the reds of the legend. Would it be possible to write down the names right next to the corresponding convex hulls?
L. 895: Position remains the same relative to which other analysis? Maybe make a reference to text and/or figure (I guess Fig. 5) here.

Methods

Are the methods appropriate to the aims of the study, are they well described, and are necessary controls included? Choose an item.

Conclusions

Are the conclusions adequately supported by the data shown? Choose an item.

Reporting Standards

Does the manuscript adhere to the journal’s guidelines on minimum standards of reporting? Choose an item.

Choose an item.

Statistics

Are you able to assess all statistics in the manuscript, including the appropriateness of statistical tests used? Choose an item.

Quality of Written English

Please indicate the quality of language in the manuscript: Choose an item.

Declaration of Competing Interests

Please complete a declaration of competing interests, considering the following questions:

- Have you in the past five years received reimbursements, fees, funding, or salary from an organisation that may in any way gain or lose financially from the publication of this manuscript, either now or in the future?
- Do you hold any stocks or shares in an organisation that may in any way gain or lose financially from the publication of this manuscript, either now or in the future?
- Do you hold or are you currently applying for any patents relating to the content of the manuscript?
- Have you received reimbursements, fees, funding, or salary from an organization that holds or has applied for patents relating to the content of the manuscript?
- Do you have any other financial competing interests?
- Do you have any non-financial competing interests in relation to this paper?

If you can answer no to all of the above, write 'I declare that I have no competing interests' below. If your reply is yes to any, please give details below.

I declare that I have no competing interests

I agree to the open peer review policy of the journal. I understand that my name will be included on my report to the authors and, if the manuscript is accepted for publication, my named report including any attachments I upload will be posted on the website along with the authors' responses. I agree for my report to be made available under an Open Access Creative Commons CC-BY license (http://creativecommons.org/licenses/by/4.0/). I understand that any comments which I do not wish to be included in my named report can be included as confidential comments to the editors, which will not be published.

Choose an item.

To further support our reviewers, we have joined with Publons, where you can gain additional credit to further highlight your hard work (see: https://publons.com/journal/530/gigascience). On publication of this paper, your review will be automatically added to Publons, you can then choose whether or not to claim your Publons credit. I understand this statement.

Yes Choose an item.
